# User-Centred Design of a Final Results Report for Participants in Multi-Sensor Personal Air Pollution Exposure Monitoring Campaigns

**DOI:** 10.3390/ijerph182312544

**Published:** 2021-11-28

**Authors:** Johanna Amalia Robinson, Rok Novak, Tjaša Kanduč, Thomas Maggos, Demetra Pardali, Asimina Stamatelopoulou, Dikaia Saraga, Danielle Vienneau, Benjamin Flückiger, Ondřej Mikeš, Céline Degrendele, Ondřej Sáňka, Saul García Dos Santos-Alves, Jaideep Visave, Alberto Gotti, Marco Giovanni Persico, Dimitris Chapizanis, Ioannis Petridis, Spyros Karakitsios, Dimosthenis A. Sarigiannis, David Kocman

**Affiliations:** 1 Department of Environmental Sciences, Jožef Stefan Institute, 1000 Ljubljana, Slovenia; rok.novak@ijs.si (R.N.); tjasa.kanduc@ijs.si (T.K.); david.kocman@ijs.si (D.K.); 2 Jožef Stefan International Postgraduate School, 1000 Ljubljana, Slovenia; 3 Atmospheric Chemistry and Innovative Technologies Laboratory, NCSR Demokritos, 15310 Athens, Greece; tmaggos@ipta.demokritos.gr (T.M.); demetra.pard@gmail.com (D.P.); mina.stam@ipta.demokritos.gr (A.S.); dsaraga@ipta.demokritos.gr (D.S.); 4 Swiss Tropical and Public Health Institute (Swiss TPH), CH-4051 Basel, Switzerland; danielle.vienneau@swisstph.ch (D.V.); benjamin.flueckiger@swisstph.ch (B.F.); 5 University of Basel, CH-4001 Basel, Switzerland; 6 RECETOX, Faculty of Science, Masaryk University, 62500 Brno, Czech Republic; ondrej.mikes@recetox.muni.cz (O.M.); celine.DEGRENDELE@univ-amu.fr (C.D.); ondrej.sanka@recetox.muni.cz (O.S.); 7 Laboratory of Chemistry and Environment, Aix Marseille University, 13003 Marseille, France; 8 Institute of Health Carlos III (ISCIII), National Environmental Health Centre, Department of Atmospheric Pollution, 28220 Madrid, Spain; sgarcia@isciii.es; 9 Department of Science, Technology and Society, University School for Advanced Study IUSS, 27100 Pavia, Italy; jaideep.visave@eucentre.it (J.V.); marco.persico@iusspavia.it (M.G.P.); sarigiannis@auth.gr (D.A.S.); 10 EUCENTRE, European Centre for Training and Research in Earthquake Engineering, 27100 Pavia, Italy; alberto.gotti@eucentre.it; 11 Environmental Engineering Laboratory, Department of Chemical Engineering, Aristotle University of Thessaloniki, 54124 Thessaloniki, Greece; dimitris.chapizanis@gmail.com (D.C.); ioannis.petridis89@gmail.com (I.P.); spyros.karakitsios@gmail.com (S.K.); 12 HERACLES Research Center on the Exposome and Health, Center for Interdisciplinary Research and Innovation, 57001 Thessaloniki, Greece

**Keywords:** user-centred design, air pollution exposure campaign, report to participants, communication, focus group, design thinking

## Abstract

Using low-cost portable air quality (AQ) monitoring devices is a growing trend in personal exposure studies, enabling a higher spatio-temporal resolution and identifying acute exposure to high concentrations. Comprehension of the results by participants is not guaranteed in exposure studies. However, information on personal exposure is multiplex, which calls for participant involvement in information design to maximise communication output and comprehension. This study describes and proposes a model of a user-centred design (UCD) approach for preparing a final report for participants involved in a multi-sensor personal exposure monitoring study performed in seven cities within the EU Horizon 2020 ICARUS project. Using a combination of human-centred design (HCD), human–information interaction (HII) and design thinking approaches, we iteratively included participants in the framing and design of the final report. User needs were mapped using a survey (*n* = 82), and feedback on the draft report was obtained from a focus group (*n* = 5). User requirements were assessed and validated using a post-campaign survey (*n* = 31). The UCD research was conducted amongst participants in Ljubljana, Slovenia, and the results report was distributed among the participating cities across Europe. The feedback made it clear that the final report was well-received and helped participants better understand the influence of individual behaviours on personal exposure to air pollution.

## 1. Introduction

The rise of low-cost personal air monitoring devices has democratised environmental health decision making, enabling scientists to involve the public in air quality (AQ) monitoring programmes. The small size of these devices, their low cost, high temporal resolution for data capture and internet connectivity for remote access facilitate their use in large-scale studies of multiple stressors [1,2]. It is known that personal exposure to air pollution depends on individual trajectories and activities [3], and exposure studies have demonstrated the need for data with a high spatio-temporal resolution in order to obtain a rigorous personal exposure assessment [4,5]. Exposure to air pollution is a serious threat to human health, as even low-level exposure to pollutants is linked to acute systemic inflammation and cardiovascular mortality and morbidity [6,7,8].

Reporting the final results of personal exposure campaigns to the study participants has not always been the practice [9,10], and even if they are, there is no guarantee that the report is comprehensible to the non-specialist. We should also not take for granted the participants’ desire to see final data [10,11]. Rather, it is every individual’s right to know or not to know [12,13,14].

The rise of low-cost personal air monitoring devices brings both opportunities and challenges in communicating results. In addition to the challenges brought by the quality of the data [15,16], it is challenging to communicate the significance of specific parameters that do not have established health guidelines or when health effects are uncertain [17,18]. However, risk communication principles can help to frame the messages [19].

Public understanding of science and environmental health literacy is partially acquired through formal schooling, but life-long learning of science topics is also inspired by free-choice learning due to personal interest, need or curiosity [20]. Participatory projects offer a playground for the public to pursue individual learning opportunities and feed their curiosity [21]. Mundane events, such as exposure to low concentrations of air pollution, can, according to Wolff et al. [22], be easily ignored or underestimated. This phenomenon is called probability neglect, which is a bias on people’s perception of risk vs. its probability. For this reason, there is a need to involve the public in disputes over inclusive risk governance communication on issues that affect their everyday lives [23]. Involvement will create heightened conceptual awareness, which can help participants make informed decisions [24], and creating communication material that prompts emotional response can shape participants’ perceptions [25].

According to Hubbell [26] and Keune [27], challenges in data interpretation highlight the need for a more inclusive two-way (risk) communication between the scientists running AQ exposure campaigns and the public. In order to improve the comprehension of reports and overcome the uncertainties related to communicating AQ information, a human-centred design (HCD) approach can further help to meet user needs and expectations [28,29]. 

Scientists should not be the only ones deciding what information is provided since the way in which participants interpret information and build contextual awareness, as well as their priorities, skills and needs, can differ from the scientists [25,30]. Co-designing the communication output with the participants can lead to improved environmental health literacy, increasing the message’s effectiveness and ultimately influencing behavioural change [31,32,33,34]. Public participation in scientific research can also enhance a participant’s awareness and knowledge of the subject [35,36,37,38].

Using a case study example, in this paper we aimed to develop a user-centred design (UCD) model for information design as a reference for environmental health scientists to effectively communicate results to participants involved in citizen science. Furthermore, we aimed to meet the participants’ needs and increase the participants’ environmental health literacy by involving them in three iterative cycles of the design process through surveys and focus group discussion, where their feedback was used to frame and evaluate the content of a final individual results report.

## 2. Methods

In this section, we discuss the study settings, participants, data collection framework and analysis.

### 2.1. Study Setting, the ICARUS Campaign

The UCD study described in this manuscript is based on data collected during the EU Horizon 2020 ICARUS project multi-sensor personal air pollution exposure monitoring campaigns. The overarching goals of the ICARUS campaigns were to collect data on external environmental exposure of individuals and exposure determinants by combining location, activity and air pollution data in different microenvironments. The resulting individual exposure was then to be communicated back to the participants at the end of the campaigns. The participants were purposely not shown the data during the campaign because the ICARUS project aimed to apply agent-based modelling [39], and seeing real-time data could affect a participant’s behaviour. The project included winter (heating) and summer (non-heating) sampling campaigns, where participants from seven European cities (Athens, Basel, Brno, Ljubljana, Madrid, Milan, Thessaloniki) carried three personal monitoring devices: a portable sensor for particulate matter (PPM), a smart activity tracker (Garmin Vivosmart 3) and a silicone wristband as a passive sampling for organic pollutants. In addition, participants had one static indoor air quality (IAQ) unit (uHoo) for collecting the following indoor air quality parameters: carbon monoxide (CO), carbon dioxide (CO_2_), volatile organic compounds (VOCs), particulate matter (PM_2.5_), ozone (O_3_), nitrogen dioxide (NO_2_), temperature, humidity and air pressure. Participants also recorded their activities, e.g., cooking, cleaning, smoking, in Time Activity Diaries (TADs) for potential sources of air pollution [40] with one hour accuracy. In addition to the silicone wristbands, which provide an integrated information on exposure over the seven days of sampling, all the devices collected data with a high frequency of minute resolution or less. This high frequency enabled deployment in everyday life conditions, although it is known that variation in speed and sensor placement with respect to direction of movement can affect the results [41]. More details on the campaigns can be found in [42,43].

#### 2.1.1. Participants

The ICARUS recruitment strategy included asking primary recruits to inquire if other people living in the same household would also like to participate. The UCD research was conducted amongst all participants in Ljubljana, Slovenia, and who were involved in the pre- and post-campaign surveys and were part of the focus group. The recruitment strategy used in the campaigns enabled a more comprehensive array of participants’ profiles, thereby extending the profile of those interested in participating [44]. This meant that vulnerable and hard-to-reach population groups were also represented, e.g., children, elderly, pregnant, low-income families, low educated and those with pre-existing health conditions. The average age of all participants in ICARUS as a whole was 38, with 15% < 18 years, 73% being highly educated and 49% from middle-income families. Table 1 summarises the participant characteristics and distinguishes between Ljubljana participants and the overall participant pool in the seven participating cities (additional information can be found in Supplementary Materials File S1 in Tables S1–S10). 

#### 2.1.2. Research Team

The ICARUS research team undertaking the campaigns and designing the final results report consisted of international experts from various backgrounds. The case study researchers were mainly from natural sciences, e.g., chemistry, physics, environmental sciences, environmental epidemiology, exposure science, environmental and chemical risk management and geography. Several had practical expertise in social sciences and science communication. Moreover, the team behind the final results report also included database management experts, who wrote the codes to generate the reports [45]. The team’s diversity enabled different perspectives to be taken into account and sufficient collaboration over design, including, for example, communication of user needs and feedback and implementing trade-offs while considering technical limitations and meeting the objective of the report.

### 2.2. User-Centred Design

Individual exposure is multiplex as the concentrations of some air pollutants may vary over small spatial and temporal scales; thus, communicating air pollution exposure to non-experts requires a holistic approach. We combined methods and ideologies from a human-centred design (HCD) landscape to form a user-centred design (UCD) model to design the communication information material. For simplicity, we referred to our proposed approach as UCD despite it having aspects from other related approaches. Our UCD followed the activities of HCD with aspects of human–information interaction (HII) [24], six design thinking principles [46] and practical recommendations from other case studies, e.g., Golumbic et al. [47]. Unlike an HCD framework, which focuses on improving interactive products, services and systems, the human–information interaction approach focuses on the (communication of) information itself [24]. In addition, design thinking ideology is a user-centric approach to problem solving of complex and multifaceted issues [46]. 

**Figure 1:**
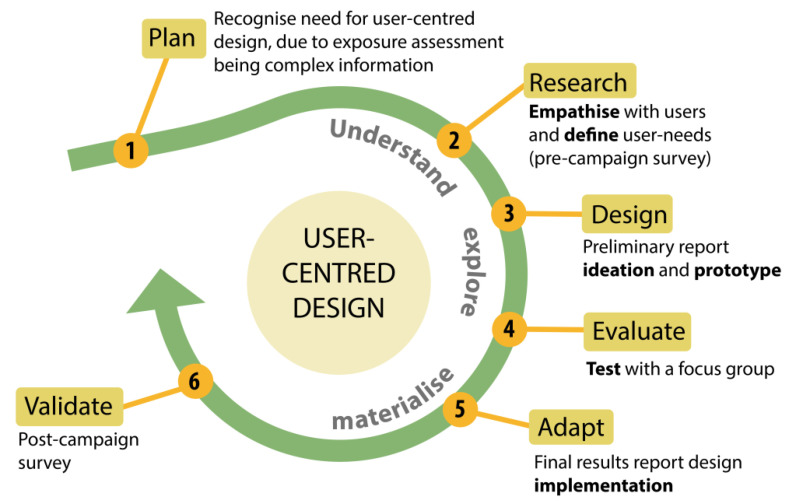
A life-cycle model of the UCD results report in the ICARUS multi-sensor personal exposure campaign.

The user-centred design was implemented as a life-cycle model (Figure 1) and incorporated in the project schedule with participant feedback as part of an iterative process to improve the report’s content. The participant involvement was implemented in steps two, four and six in order to define their needs, to adjust the report according to their feedback and to validate the final product. 

The six applied principles of design thinking include the following: empathise, define, ideate, prototype, test and implement [46] (bolded in Figure 1). The steps in implementing the UCD model included:

Step (1) Defining the context of use and recognising the need for UCD, taking into account the complexity of individual exposure data;
Step (2) Identifying and understanding user needs and preferences by obtaining feedback early on in the process through a pre-campaign survey (see Supplementary Materials File S1);
Step (3) Discussing visualisation ideas and creating a prototype report (Supplementary Materials File S2);
Step (4) Creating a focus group (*n* = 5 individuals), testing the preliminary design and facilitating fine-tuning according to group feedback (Supplementary Materials File S2–S4);
Step (5) Adapting visualisations according to the focus group results while taking into account technical requirements (Supplementary Materials File S5);
Step (6) Validating and assessing whether user requirements were met with an online post-campaign survey. (Supplementary Materials File S1)

A simplified version without details of the case study is provided in the graphical abstract for researchers to adapt.

#### 2.2.1. Plan: Recognizing the Need

In the ICARUS campaign, data were collected at high spatial-temporal scale, e.g., minute accuracy, and from various sources, e.g., indoor and outdoor together with GPS coordinates, creating a multiplex dataset. For maximising the communication output, the information must meet individual needs, which is achieved by upfront analysis of participants’ information needs and goals [48]. A design that complies with user needs instead of assumed requirements was decided upon and developed using principles from the HCD landscape. This also meant involving end-users in an iterative evaluation process involving prototypes and producing feedback until the design met the end-user requirements. Systems that are designed without end-user input might fail to provide comprehensive information.

According to HII concepts, the results report needs to communicate and enable the participants to understand the concept (e.g., background), situation (e.g., individual exposure) and relationships and interactions (e.g., the type and level of physical activity and air pollution levels) [24]. When the participants interconnect pieces of information, they will become aware of the different behaviours or actions that may be affecting their exposure. Similarly, in inclusive risk governance, a person will seek to understand the risk from their perspective [23]. That is why the results report should be designed to address the above elements. Displaying simple data, e.g., raw values, does not enable an inexperienced participant to comprehend the situation, whereas providing information with more intelligent output, e.g., post-processed information, can add context and help in understanding more complex relationships [24,49,50]. According to Albers [24], a complex communication situation should include the proper amount of information to maximise the communication output. In addition, jargon should be avoided in communicating scientific results to the public to ensure comprehension and readability for a wider audience [51], which is why the comprehension of the text in the report had to be tested amongst the participants.

We decided to study user needs regarding data visualization through a pre-campaign survey, fine-tuned within a focus group and validated with a post-campaign survey. Data visualisation refers to the representation and presentation of data to facilitate understanding [52]. From existing guides, it is known that using conventional tables or lists to display complex datasets can make it difficult to detect patterns, and other data visualisation types such as charts are preferred [25]. The visualisation solution should be trustworthy, accessible and elegant [52]. According to Allen [25], conceiving, creating, interpreting and responding to visualisations is a dynamic, complex space, and he suggests that visualisation practices influence a participant’s engagement. Similarly, Wong-Parodi et al. [53] emphasise that the most effective visualisations are those coupled with messages calling for action to reduce individual risk. The above speaks for the importance of including a list of recommended actions in the final results report, upon which individuals can act.

Previous research [54,55] has indicated that oversimplification of air pollution in the form of air pollution index values might not be the most interesting for the participant since it does not provide a sufficient level of fine-grained information and might not always be representative. Alternatively, the use of low-cost personal air-quality measuring devices provided an opportunity to delineate and visualise personal exposure levels within short time intervals, highlighting peak acute exposure levels that occurred throughout the week. In addition, the collected GPS and Time Activity diary (TAD) data could help identify exposure pathways at specific times and locations and help formulate more accurate individual exposure profiles.

#### 2.2.2. Research: Pre-Campaign Survey and User Needs

Defining communication value is a difficult task [24]. We approached this challenge by mapping the user needs with a pre-campaign survey (*n* = 82) conducted face-to face, in an interview-like setting and recorded on a paper form, at the participant’s house. The survey mapped the user needs and preferences about visualisation and their motivations, risk perception and behavioural intentions. Part of the dataset was derived from surveys analysed by Robinson et al. [43].

At the start of the campaign, participants were asked to provide suggestions on the kind of information and visualisation they wished to receive in the final report (Tables S13–S14 in Supplementary Materials File S1). It was felt important to provide the participants with the opportunity to explain their suggestions for visualisation ideas to discover features or innovative ways of displaying the data. In addition, we asked how much they would be interested in a set of pre-described ideas of data display (Tables S15–S17, Figure S1).

Trust is a significant factor in communicating scientific results to the public. It includes both trustworthiness towards the data, e.g., its uncertainty, and in the ones communicating it [25,52]. In the light of the new European General Data Protection Regulation (EU GDPR 2016/679) [56], we wanted to assess participants’ trust concerning personal data management (Tables S28 and S29 in Supplementary Materials File S1).

The paper forms were digitalized, and the findings were analysed in Excel, where open-ended questions were coded and suggestions listed and general statistics and frequencies were analysed for the Likert type questions. 

#### 2.2.3. Design: Preliminary Report

The ideas for the preliminary report were drafted based on initial user needs and collaborative efforts of the project consortium. Some visualisation ideas and decisions were discussed over email, online conference meetings and in face-to-face project meetings. We also included a hands-on workshop about data harmonisation, data visualisation and data quality. Ideas from different participating cities were discussed, and it was decided to organise a focus group to evaluate the preferences and comprehension of different visualisation options amongst the participants.

Low-cost sensors come with a particular uncertainty, and missing data from malfunction or other causes is an issue that scientists need to consider to not disappoint the participants [54,57]. Adding information about technical uncertainty is considered a positive practice when communicating the results [58]. The challenges were identified, and steps for data harmonisation, cleaning and fusion with an R script and models to identify outliers and fill data gaps were established [45].

A preliminary report was drafted and generated in Ljubljana. Pre-existing guidelines and good practices were considered, and trade-offs were discussed while accommodating the initially mapped user needs and visualisation suggestions from the other participating cities. Deciding on a design was a difficult task, as there is no one-fits-all solution. In addition, different participants might have a different level of numeracy, and sometimes simplification of the message is necessary to make sensor data more comprehensible [26].

#### 2.2.4. Evaluation: Focus Group

A focus group is an in-depth group interview with discussion and enables a collection of views about a specific subject of concern. It is used to assess needs, preferences and attitudes of participants, and the results can help in decision making. An optimal number of participants in a focus group is between five and eight [27,59]. This allows gathering insights from all participants while maintaining control over the discussions. In larger groups, this becomes difficult and limits opportunities for those who would speak less.

Invitation to the focus group was sent to 12 participants. Following Keune’s [27] and ISO HCD 2010 recommendations, participants for the focus group were selected to be representative and those who also showed interest to be actively involved. Only adult participants were invited. The focus group was organised at the onset of the SARS CoV-2 pandemic in Slovenia, causing some to decline to attend an in-person event. However, a final number of five in-person attendees is, according to Virzi [60], sufficient to identify the majority of usability issues. Some participants suggested providing feedback electronically instead, and we received 19 answers to the same questions discussed during the focus group through an online questionnaire.

A one and a half hour long focus group meeting was organised (Table 2) to ascertain whether participants correctly perceived the draft visualisations of the results report and provide additional user feedback. The displayed graphs and other visualisations used in the focus group were detailed and realistic, e.g., anonymous data from actual participants were used. Three members of the scientific project group were present at the focus group, with one acting as the facilitator. The focus group was organised after the sensor campaign and before finalising the participants’ results reports. The focus group discussion was recorded, transcribed and translated into English. The main findings were implemented straight away to improve the draft report, while details of the focus group and its findings were summarized for this manuscript. Additional information about the organised focus group can be found from the Supplementary Materials, including the translated transcripts with a list of actions based on the participants’ feedback (Supplementary Materials File S3) and the materials used during the focus group, i.e., PowerPoint presentation (Supplementary Materials File S4) and the preliminary report (Supplementary Materials File S2).

#### 2.2.5. Adapt: Final Results Report

The design of the results report was refined and improved in response to user-centred evaluation and feedback from the focus group. Any conflicts were resolved considering reasonability, technical limitations and incorrect interpretations of figures (an example report can be found in Supplementary Material File S5). An R script was written to generate the final uniform reports to all participants for all participating cities [45]. The final results report was translated to the different languages by the local case study researchers and sent to the participants by email as a PDF document in April 2020. 

#### 2.2.6. Validate: Post-Campaign Survey

The Ljubljana participants received an online post-campaign survey invitation together with the final results report email. The post-campaign survey meant that the participants were able to assess the usability and comprehensibility of the report and provide further feedback to improve similar reports in the future. Participants were then asked how the results had influenced their behaviour and risk perception. Thirty-one participants answered the survey.

## 3. Results

This section describes the results of the UCD steps two to six, where participant views were used to frame and evaluate the content of a final individual results report.

### 3.1. Research: Pre-Campaign Survey and User Needs

The pre-campaign survey revealed that the participants wanted both visualisations that provide an opportunity to interpret their exposure and the influence of behavioural choices and also a prescription of activities on how to reduce one’s exposure.

A total of 15% of the participants wanted to have all the possible data about their exposure, in either raw format, charts or numerical values (Table S14 in Supplementary Materials File S1). They also wanted a comparison with limit values (12%), comparison with other participants in the same city (11%) and spatial (12%) and time trends (10%). Five percent mentioned that they would like to have text results. Moreover, participants wished to receive data evaluation, conclusions, recommendations to mitigate exposure and information on possible adverse health effects. Nine percent of participants also specified that they wanted to receive the results electronically, e.g., a PDF file, while Microsoft’s Excel was suggested for raw data. Eighteen percent of participants were happy to receive just an executive summary. While some hoped to have the raw data, others preferred summary data or simple visualisation. Some pointed out that they would like to see any unusual observations. 

The participants were especially interested to know about their personal exposure and the air quality in their surroundings. Some also mentioned CO_2_ levels indoors, and several hoped that the results would show how clean the air is in the local area, while others hoped the results would reveal something upon which they could act. The participants also showed interest in air quality and health parameters. The participants were also interested in the accuracy and reliability of the monitoring devices and what happens in the case of “bad” data. 

The following are specific quotes from the participants: 

LJU_P033: *“Similar to doctors reports, value ranges and limit values, data tables, textual and charts with explanations.”*

LJU_P093: *“Pollution levels and physical activity displayed with time trends with short time intervals (1s), in electronic form, in data tables.”*

The participants repeatedly mentioned that they wished to see the results before taking any action and would prefer to see the data in real-time. All the participants wanted to receive the results report and hence pursue their right to know.

From the preliminary interest list (Tables S15–S17 in Supplementary Materials File S1), on a scale from one to five, the participants expressed interest in Where your maximum/minimum air pollution exposure occurs (Mean 4.72) followed by During which activity you are most/least exposed to air pollution (4.76), Suggestions to reduce your air pollution exposure (4.55), Individual pollution concentrations (4.47), Which transportation mode contributes the most/least to your air pollution exposure (4.39), Map of your weekly whereabouts with indicative colour codes (4.37) and Is my weekly dose of air pollution less or more than others who participated? (4.36).

Each participant held a prior perception of the level of credibility of the scientific institutions that conducted the case studies and communicated the results. None of the participants thought their data would be handled inappropriately, with 79% being confident that the researchers handled their data appropriately, while 21% did not know or did not answer the question (Tables S28 and S29 in Supplementary Materials File S1).

### 3.2. Design: Preliminary Report

Ideas for visualisations for the final results report were prompted from all participating cities, and some distributed preliminary results to the participants soon after the end of the campaign. For example, in Brno, short reports, following a basic report structure providing available data from the commercially used sensor devices, were created. They included an introduction to the measured parameters and abbreviations, followed by tables with mean values from the whole campaign and participants’ decile within the Brno campaign. Each page clearly stated that the data were not certified, accredited or validated and served only as a visual output from commercially available devices. Data from PPM devices were provided upon request. Some of the earliest visualisations in Athens included summary statistics and box plots for individual indoor pollutants. Data were cleaned by removing NA values, duplicate values and other issues. Average daily and weekly concentrations of PM from the summer and winter campaigns were also used, and different fractionations of PM (PM_1_, PM_2.5_ and PM_10_) were displayed per participant for each microenvironment and activity, e.g., cooking, cleaning, smoking (derived from TADs). In Basel, for example, the preliminary report included a map of PM_10_ data created upon request with GPS data for times when participants were not at home. Follow-up calls and emails from participants in Basel and other cities demonstrated effective communication and further interest in the topic. Individuals in different cities discussed their indoor air quality values during the final visit in households when devices were collected. Some participants also suggested checking specific PPM levels for time periods where they assumed they had been highly exposed (e.g., during campfire). In Milan, winter vs. summer visualisation of the average concentrations of PM_10_ was used.

It was agreed to use a uniform format for the final report for all cities. The report’s structure was planned to include a general introduction, an overview of the air quality in households, a personal situation exposure assessment, and generalised recommendations. Creating a report with data displayed straightforwardly without giving absolute values was agreed upon, and displaying daily averages were preferred. Comparison with limit values was a subject of discussion since it could provide valuable information to the participant, although it could confuse participants due to the uncertainties in the measured data and the different time scales of the limit values. Uncertainties and the use of relative vs. absolute values were discussed. In Ljubljana, previous validation studies showed that absolute values from the IAQ sensor should not be used, while absolute values from the particulate matter monitor could [61]. The problem with the validity of absolute data from the IAQ turned the decision to provide heatmaps rather than measured values. Information about inhalation rates and intake dose assessment was not considered a priority for the report, and regarding exposure, it was decided to provide information by specific location (e.g., home vs. commute vs. work) rather than by physical activity. 

### 3.3. Evaluation: Focus Group

During the focus group discussions, the participants had issues deciphering some of the suggested visualisations due to missing labels and different axis labels, which made comparing different charts complicated. Supplementary Materials File S3 (chapter 4. List of suggestions from the participants and actions taken based on them) contains a more detailed description of visualisations that were difficult to comprehend and taken to improve them.

### 3.4. Adapt: Final Results Report

An example report can be found in Supplementary Materials File S5. The adaptation of the results report included compromises between participants’ requirements and technical limitations. The PM, for example, was considered an important parameter and was presented in different charts. In the most basic form, using daily averages together with WHO guidelines were displayed. A more fine-grade visualisation was not feasible due to space limitations and because WHO guidelines for PM_2.5_ and PM_10_ only include daily values. In addition, charts with additional context were prepared and included specific activities or locations of the participants and heartbeat data with a more fine-grade hourly data. Winter and summer campaigns were plotted separately. 

**Figure 2:**
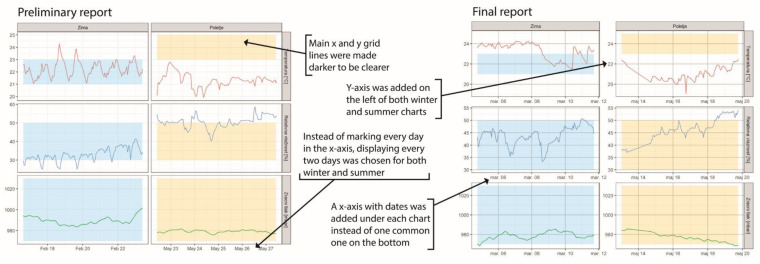
Example of adjustments to the visualisation based on the focus group discussions. Final figure caption: “Meteorological conditions in one household during the winter (**left**) and summer (**right**) campaigns. The top plot displays temperature, followed by relative humidity and air pressure. Optimal ranges for all three parameters are also displayed and coloured in yellow (summer) and blue (winter)”.

An example of adjustments to the visualisation based on the focus group discussions is displayed in Figure 2, while the whole final report can be found in Supplementary Materials File S5.

QA/QC steps to remove outliers with extremely high concentrations (over 240 µg/m^3^) were performed in R for PM data following predetermined criteria of consecutively occurring measurements, as reporting these types of erroneous results could have been worrying for the participants. Conversely, removing outliers might have unintentionally underestimated someone’s exposure to severely high concentrations, e.g., time spent in a nightclub where smoking was permitted. In most cities, only a small number of the participant’s (up to 12%) data had significant outliers, i.e., over 10% of all measurements of PM_10_ were more than 240 µg/m^3^, and only the participants can know what they did when the peak values occurred. The codes were checked and revised for flaws such as double reports, mixed summer/winter seasons, wrong participant numbers, typos and the deletion of some lines. The individual reports were still manually checked for any mistakes before sending them to the participants. The results from the silicon wristbands were not distributed in the final results report as the laboratory analyses of passively sampled organic chemicals were still ongoing.

**Figure 3:**
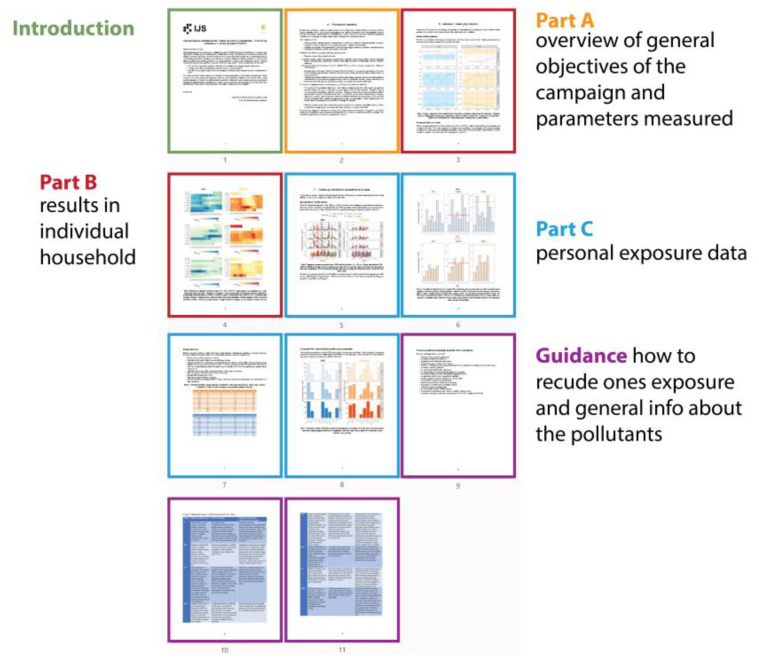
Structure of the final results report.

The structure of the report (visually displayed in Figure 3) followed the planned initial structure, and the text, e.g., in the introduction, was fine-tuned collaboratively with researchers from participating cities. The final report (Supplementary Materials File S5) was 11 pages long and included charts (histograms, line columns), tables and heatmap visualisations with colour-coded additional information of activities and guideline values or optimal ranges where applicable.

### 3.5. Validate: Post-Campaign Survey

The post-campaign survey validated the usability and comprehensibility of the report amongst 31 participants.

An emotion of surprise was present in the group who evaluated the final results report (Tables S18–S20 in Supplementary Materials File S1). Almost half (48%) were surprised by their data, especially about different from expected concentration values of air pollutants.

The majority (79%) indicated that the report was useful, easy to understand (79%) and contained the right amount of information (Tables S21–S25 in Supplementary Materials File S1). One-third (35%) of the participants also gave suggestions on how the report could be improved (Tables S26 and S27 in Supplementary Materials File S1). Some suggested simpler infographics for laypeople, while others would have liked more detailed ones. Suggestions for improving the charts included a better colour scale for activities and more charts displaying activity data. They also suggested the results should be displayed spatially, a comparison with other participants, an online introductory video, a public web page and an invitation to all participants to a dissemination event. 

The participating children received help from their parents to interpret the research results. In some households, the results and participation had created further discussions beyond family units. The results report was well-received both in Ljubljana and in the other participating cities. Post-campaign communication continued in all cities, and some participants reached out to the research groups to ask for additional visualisations, e.g., trajectories along their cycling trip. In some cities, e.g., in Brno, a public presentation of the results was organised, where all participants were invited.

Due to the nature of low-cost sensors, some sensors failed to record any data while others malfunctioned, resulting in data gaps. The result was that some participants’ reports had missing data resulting in blank charts, which left participants feeling disappointed, especially given all their effort. Equally, many understood the nature of low-cost sensors as a possibility at the beginning of the campaign. Some participants, both in Ljubljana and in the other case study cities, contacted the local researchers to ask about the missing values. Others wanted to know, for example, if they were above or higher than the study average or to explain to the researchers the reason for the observed peak values in their data. 

Mitigation guidance was presented at the end of the report. Over half (58%) of the participants reported having made behavioural changes, and 36% thought their air pollution exposure was higher than expected.

## 4. Discussion

Deciding on a universal design for a final results report is a challenging task. Individual characteristics influence how participants interact with health information, making it difficult to meet everyone’s needs [62]. Information exists within a continuum, and individuals interpret and reflect on the information based on their own experiences, feelings, assumptions and beliefs [24,25]. This was also emphasised from the participants’ side during the focus group discussion: “It was also interesting to see how some of us understood, while others did not. You could see how all of us look at things differently.” 

The involvement of the participants in the three report design stages enabled us to fine-tune the details and make sure the final report was fit-for-purpose and comprehensible to the majority of participants. Another reason for using a common approach in the reporting on the European scale was to create a harmonised analysis of the data itself. Involving participants in the design provided meaningful input to the content and provided a user perspective. Adding another cycle before distributing the final report would most likely further increase the comprehensibility. An additional step would be to target different sub-groups specifically, e.g., children, elderly, health-suppressed individuals, participants with low socioeconomic status (SES) or the highly educated, by using personas to divide the subgroups and adjust and enhance the communicated message accordingly. In addition, to improve the report using UCD, including a science communication expert to assist in designing the communicated results would be beneficial [63]. This inclusion of an expert would also contribute towards higher environmental health literacy [64].

The focus group enabled us to explore the report’s content in depth through group discussions, e.g., comprehension of suggested visualisation and possible misunderstandings, feedback for improvements, and refining user needs at the end of the project. The discussion also reflected their environmental health literacy. A focus group method enabled us to obtain a common impression and in-depth information in a short time. The involvement of the participants enabled us to frame and formulate risk information about their exposure to be communicated in a way that a participant could comprehend.

The participants’ needs, feedback, and suggestions reflect the users’ capabilities, characteristics, and experience, agreeing with ISO recommendations. The detailed user studies were conducted in Ljubljana, but given that the report’s reception in other participating cities was positive, the design was well suited to the community that participated all across Europe. Hence, similar design aspects could be implemented elsewhere.

Effective and efficient communication methods based on data from low-cost sensor devices is an ongoing debate. The study advances the current practice in environmental health communications where results of an exposure study are communicated retrospectively to the participants by providing insights and evaluations of user needs through an UCD process. Careful planning, time and effort is needed to perform a UCD study to meet the user needs while adapting to the challenges and limitations of the technology and information design. We illustrated increased understanding of user needs and demonstrated an approach to support and validate it.

Being trustworthy is also a factor. The majority of the participants expressed confidence that we would handle data appropriately, while the rest were unsure or did not answer the question. None explicitly expressed “worry”.

The report was considered lengthy at 11 pages and was one of the issues discussed while preparing the report. However, the participants confirmed that the amount of information was “just right”, which is appreciated, especially at a time when people are experiencing information overload [65]. Despite this, some participants preferred timelier reporting. 

The final results reports were provided almost a year after the campaign had finished. While this can be unfavourable, they would have been less relevant for the participants if delivered earlier and without going through a UCD process. If data were shown in real-time, or the report was sent earlier, we could have increased the communication value, as communication needs to be relevant and on time [24,66]. Thus, this would increase their intention to change behaviour [43]. A step forward would be to create an online version of the results report, allowing participants to change variables to suit their contextual needs, facilitate comprehension and nurture curiosity. The number (*n* = 31) of the post-campaign surveys reflects how some interest was lost compared to the pre-campaign survey (*n* = 82). In most cases (i.e., in 28 households), only one person from the household answered the post-campaign survey instead of the whole family, which can explain this difference.

Using hourly and daily averages instead of higher time resolution visualisation, we could not provide detailed information about each activity, location and time. The final interpretation of the results was left to the participants, who had information about their specific activities if they still remembered. Automating the information content related to individuals spatial location and activity mode using data mining techniques, which classifies relative location and activity using supplementary information available, could further improve the interpretation of the results and remove the burden of filling out time activity diaries and remembering details [67].

Involvement in the process leads to greater understanding, and consequently, appropriate action [27], e.g., as many as 58% reported behavioural change in the Ljubljana case study. Sensitive individuals, especially those with underlying health conditions, received an opportunity to examine possible triggers if peak values were present during their study period. It is considered good practice to communicate uncertainties, e.g., sensor performance or outliers that over- or underestimate a participant’s exposure.

Collecting data on their immediate environment and receiving results about their living environment must be more motivating than campaigns that aim to collect air quality monitoring data on the city level. Robinson et al. [54] concluded that participants in an air quality study are more motivated to learn about their immediate environment and hence more likely to change their behaviour when provided with more targeted results about their living environment. Instead of the mass communication of aggregated results, the individuals received only their results, although some would have liked to see their situation compared to the other participants.

The WHO 24 h mean guideline values for some of the used AQ parameters were updated after the project had ended, e.g., PM_2.5_ was lowered to 15 from 25 μg/m^3^ and PM_10_ from 50 to 45 μg/m^3^ [68]. Future studies could study the risk perception of exposure to air pollutants in the light of measured AQ values and the new WHO guideline values.

## 5. Conclusions

This work describes and proposes a model of user-centred design (UCD) of a final results report and demonstrates a deliberate, collaborative science communication effort. The participants of the multi-sensor ICARUS campaign in Ljubljana, Slovenia, were included in the design of a final results report during three stages of the design process. The report was individualised, self-descriptive and intuitive, fit for purpose, met user expectations and provided an opportunity to learn something new. 

The UCD model is a result of lack of existing UCD models for results reporting in environmental health studies. The developed UCD model is a combination of principles and practices from the HCD landscape, design thinking and HII, the latter focusing on communicating complex information where the user makes decisions about a complex situation. The UCD approach was incorporated into the project schedule and involved collecting information on user needs and gained feedback about preliminary design solutions from an end-user perspective to improve the final design and evaluate and validate whether user requirements were met. The information presented was subjected to a series of trade-offs, to be understandable by the general public, yet not overly simplified, which would underestimate the complexity of the information, and hence to take into account the underlying situation, people’s needs and the way they come to understand the information provided. Using a UCD approach, we co-created usable content, enabling participants to comprehend the complex topic of personal exposure that they could use to make informed decisions, both being essential aspects of environmental health literacy. 

By asking participants to provide input, we were able to better meet the participants’ needs, which probably influenced the high acceptance of the information. By involving the participants in co-designing the communication output, we increased the inclusiveness of the project from traditionally contributory type ones. We recommend others to use the UCD approach to democratise science and to involve the participants in co-creating complex information.

The report provided both options to examine the results in light of established air quality standards and display individual levels of exposure for the study period. By providing several visualisations, we addressed multiple goals and the motivational drivers of the participants before their involvement. Carrying sensors in places where official air quality monitoring does not extend, e.g., at homes and in private cars, enabled the participants to understand personal exposure. The communication was effective, since it prompted a change in behaviour in the majority of the participants. This finding shows that lived experiences and co-created communicated material increases environmental health literacy by increasing the interest, awareness and understanding of the particular topic leading to taking action [32,69]. It also conforms with other literature on behavioural change [53,70], which also emphasises that information about a risk to air pollution exposure on its own is not as powerful. By providing each individual with a set of sensor devices, we enabled them to experience the air quality first-hand. Projects involving participants in collecting data should use the opportunities modern technology provide to grant the participants access to real-time (instantaneous) data in addition to online visualisation and self-exploration of data.

## Figures and Tables

**Table 1:** Participant characteristics.

	Participants in Ljubljana		Participants in All Cities	
Characteristics	Total	Percentage	Total	Percentage
**Age**				
<18	8	11%	77	15%
18–64	60	82%	398	79%
>65	5	7%	32	6%
Pregnant	1	1%	6	1%
**Gender**				
Male	39	53%	242	47%
Female	47	47%	269	53%
Other	0	0%	0	0%
Underlying health condition	26	36%	194	36%
**Education level of adult participants**				
Primary education/Not completed secondary education	4	6%	16	4%
Completed secondary education	9	14%	101	23%
Higher education	52	80%	313	73%
**Income level of adult participants**				
Lower 25%	7	11%	86	20%
Average (25–75%)	37	57%	183	43%
Upper 25%	16	25%	107	25%
Unknown	5	8%	54	13%

**Table 2:** Focus group structure.

Section	Theme	Goal	Planned	Timeline in the Recording
1	Welcome and short survey	Flashback paper survey on what participants remember about the campaign	5 min	00:00–04:10
2	Introductory PowerPoint presentation	A presentation about the project and campaign, measurement uncertainties	10 min	04:10–10:15
3	Discussion Part 1	Mapping motivations and expectations on what participants would like to learn	10 min	10:15–11:57
4	Discussion Part 2	User needs: data aggregation in most useful way according to participants ideas	10–15 min	11:57–13:40
5	Evaluation	Comprehension of suggested visualizations (paper survey)	20 min	13:40–32:29
6	Discussion Part 3	Visualization: first suggestions and their comprehension and suggestions for improvements	20 min	32:29–1:07:44
7	Discussion Part 4	Impact on behavioural change and user needs during and after the campaign	10 min	1:07:44–1:18:39
8	Conclusions and socialising	Preliminary observations from the data. Final remarks and farewell	10 min	1:18:39–1:34:41

## Data Availability

Data available on request due to privacy restrictions.

## Supplementary Materials

The following are available online at https://www.mdpi.com/article/10.3390/ijerph182312544/s1, Supplementary Materials File S1: Data analysis, Supplementary Materials File S2: Printed materials, Supplementary Materials File S3: Transcript of the voice recording and summary of changes, Supplementary Materials File S4: Focus group PPT, Supplementary Materials File S5: Example report.
