# Supplementary material for: User-Centred Design of a Final Results Report for Participants in Multi-Sensor Personal Air Pollution Exposure Monitoring Campaigns
Source: Int J Environ Res Public Health. 2021 Nov 28;18(23):12544. doi: 10.3390/ijerph182312544 (PMC8656880; doi:10.3390/ijerph182312544)
Supplement: Supplementary file 1 [file ijerph-18-12544-s001.zip › S2_printed_materials.pdf]

Supplementary material 4 for the article "User-Centred Design of a Final Results Report for Participants in Multi-Sensor Personal Air Pollution Exposure Monitoring Campaigns" submitted in *International Journal of Environmental Research and Public Health*. Printed materials used during a focus group discussion. The same material was re-created for an online questionnaire for those who could not join face-to-face.

- **Please enter your participant ID: _______________**

- **Which parameters did we measure during the campaign (check all that apply)?**

a) Particulate matter	b) Carbon monoxide	c) CO_2	d) pH	e) GPS - location

- **Which sensor units did you use (check all that apply)?**

a) pH meter	b) Activity tracker	c) uHoo stationary unit for indoor air	d) light sensor	e) silicone wristlet

- **How long did you participate in our campaign (please select only one answer)**

a) Twice for 14 days	b) 3 days during winter and 10 days during summer	c) Twice for 7 days	d) Three times for 5 days	e) One week in March

- **What did you mark in the time activity diary (check all that apply)?**

a) Smoking	b) Playing with a cat/dog	c) Working in the woods	d) Open window	e) Sleeping

- **How accurately did you mark your activities?**

a) Every 20 minutes	b) Every full hour	c) Four times a day	d) Every odd hour	e) Once a day

- **What were the aims of the campaign?**

1. Meteorological parameters indoors:

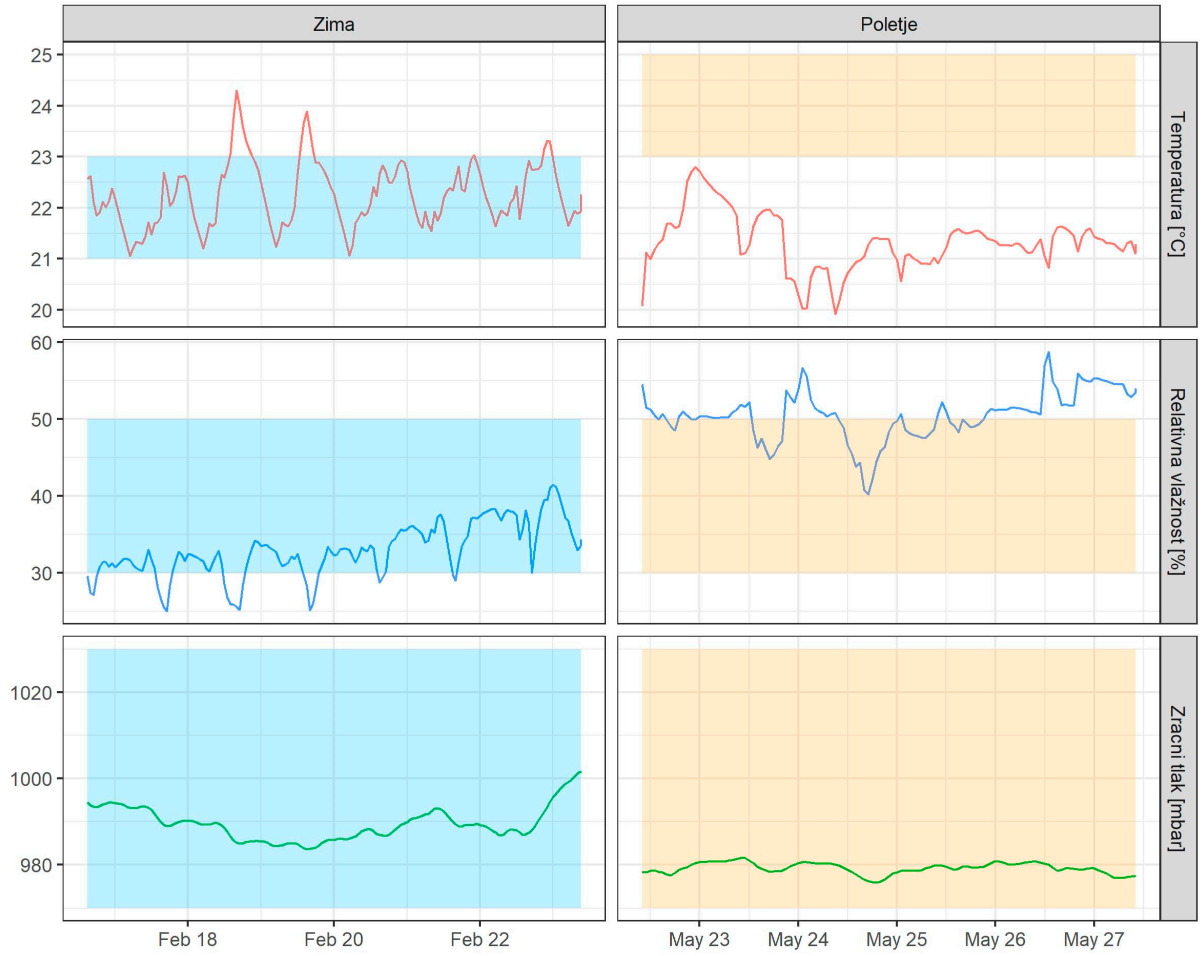

Figure 1: Meteorological conditions in your household during the winter (left) and summer (right) campaigns. The top plot displays temperature, followed by relative humidity and air pressure. Optimal ranges for all three parameters are also displayed and coloured in yellow (summer) and blue (winter).

Look at the Figure 1 and try to answer the following questions:

- When approximately was the highest measured temperature in the example household? _______________

- What is the range of recommended humidity levels in summer? _______________

2. Indoor air quality parameters

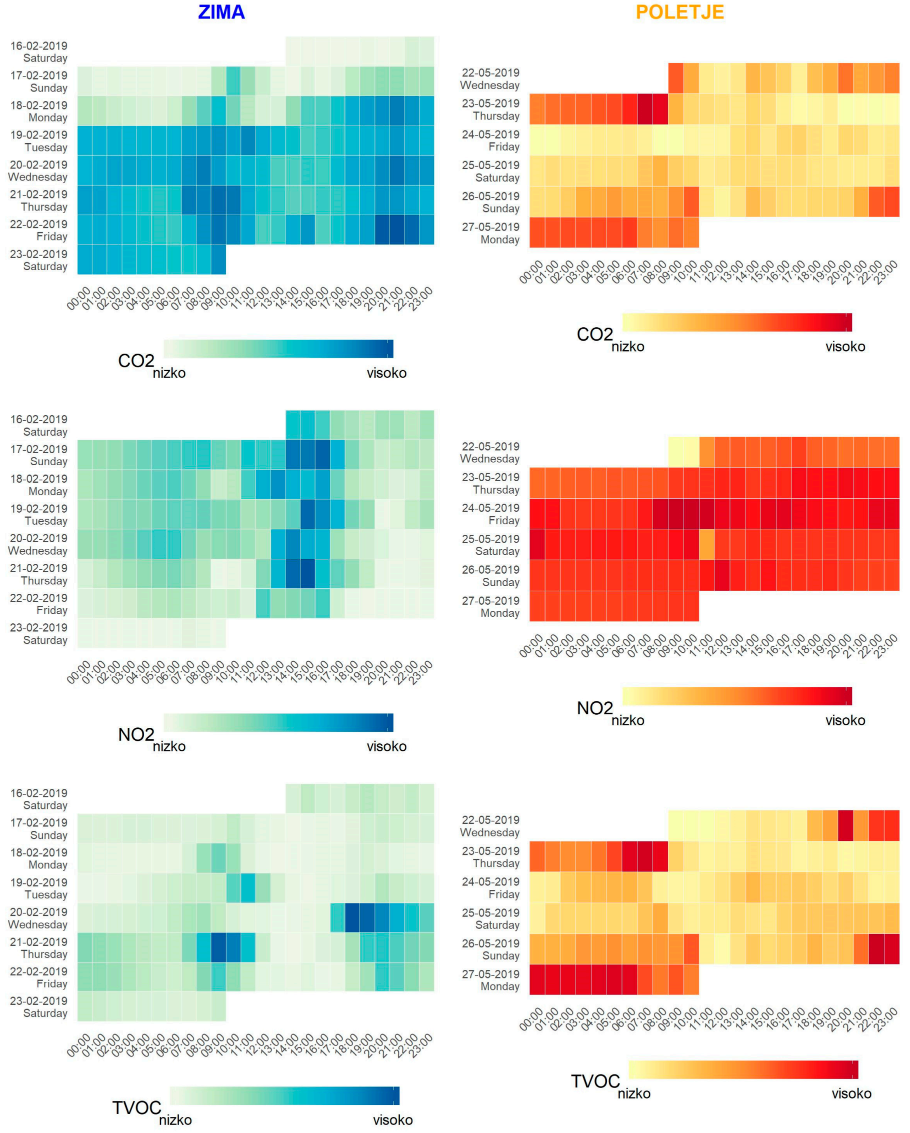

Figure 2 displays air quality parameters (CO2, TVOC and NO2) in example household for individual days in both seasons. Parameters are shown in a form of heat-maps displaying relative deviation of each parameter in comparison to highest and lowest measured values in winter (left) and summer (right) measuring campaigns. The label "low" represents the lowest measured value, while the label "high" the highest measured value. The days are displayed in English (Pondeljek – Monday, Torek – Tuesday, Sreda – Wednesday, Četrtek – Thursday, Petek – Friday, Sobota – Saturday, Nedelja – Sunday.

Look at the Figure 2 and try to answer the following questions:

- When approximately was a highest value for CO2 in summer measured?

  ____

- Are the measured NO2 values during winter higher during afternoon compared to before noon?

  ____

- Given the selected method of displaying the measured values, is it possible to directly compare the values between winter and summer? YES NO

3. Results from particulate matter meter (PM) and heart rate

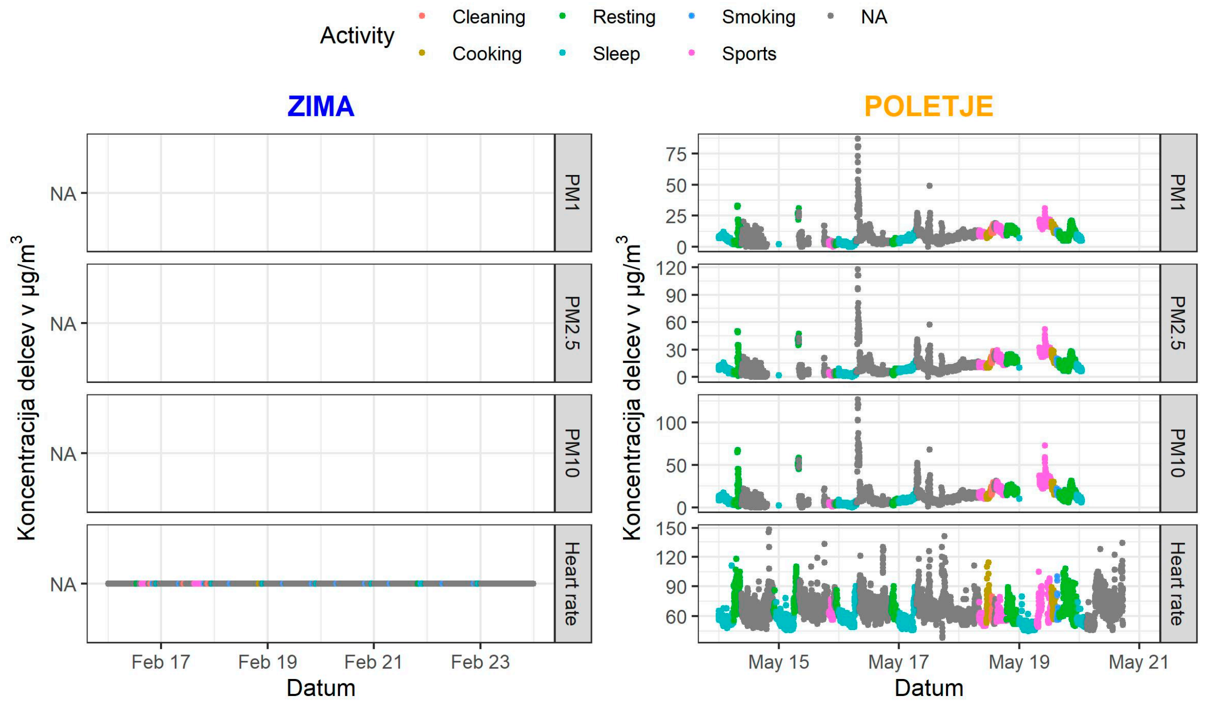

Figure 3: Measured concentrations of particulate matter (PM) in different sizes (<1, <2.5 and <10 µm displayed as PM1, PM2.5, and PM10) as well as heart rate in winter (left) and summer (right) together with activity data. The activities are displayed in English: »Cleaning« – čiščenje, »Resting« - počivanje, »Smoking« - kajenje, »Cooking« - kuhanje, »Sleep« - spanje, »Sports« - športe aktivnosti, »NA« - missing information/podatek manjka

Look at the Figure 3 and try to answer the following questions:

- How much is the highest measured concentration of $PM_{2.5}$?________

- When was the lowest heart rate measured? ____________

- How much is the highest measured concentration of PM10 measured on 19th of May during sports activity? ________

- What do the grey areas in the chart mean? _____________________________________

- What does the heart rate graph during winter (left) display? _________________

4. Daily averages for particulate matter (PM) in both seasons

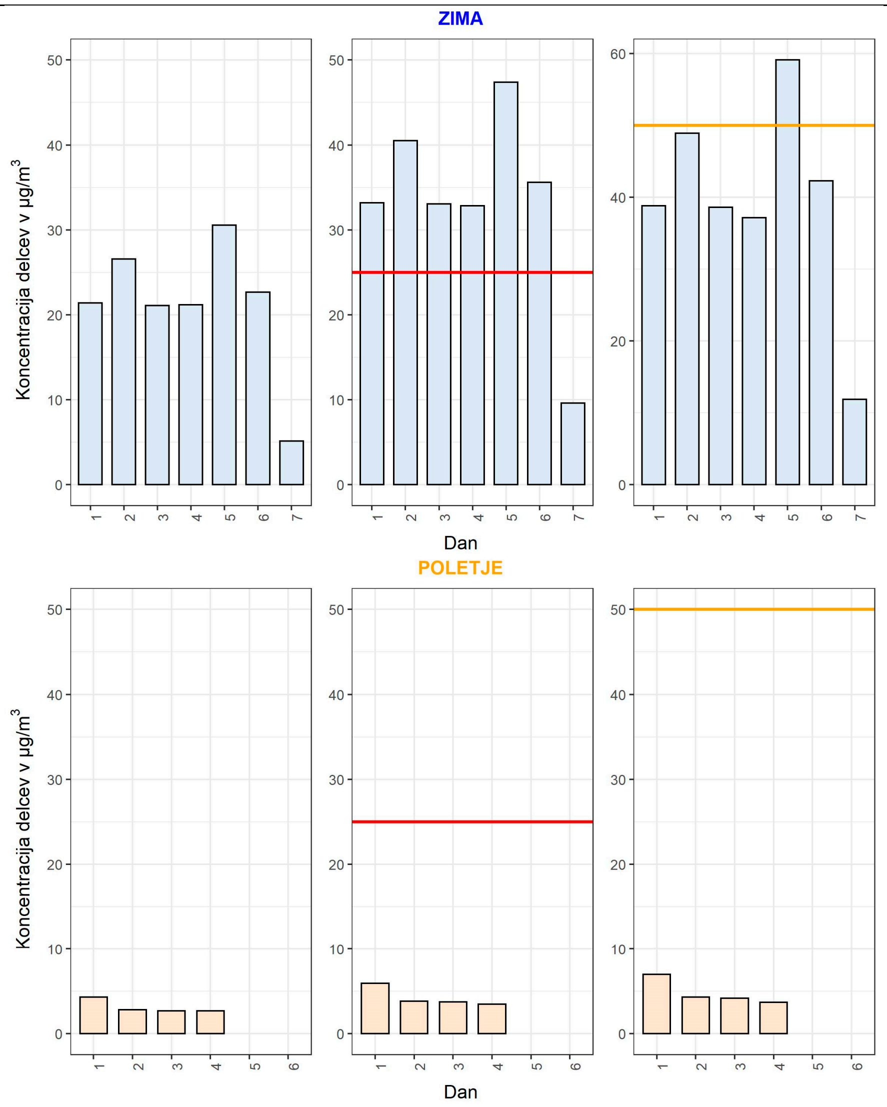

Figure 4: Comparison of average daily PM values with WHO Air Quality Guideline values for winter (upper chart) and summer campaigns (lower chart). The red line represents the WHO guideline for 24-hour average value for $PM_{2.5}$ and the orange line represents the WHO guideline for 24-hour average concentration of PM_{10}.

Look at the Figure 4 and try to answer the following questions:

- How many days a week and during which season were $PM_{2.5}$ concentrations exceeded? ________
- How many days a week and during which season were PM_{10} concentrations exceeded? ________
- How much is the limit value for $PM_{2.5}$? ________________
- Some information on PM concentrations is missing. How many days and during which season? ________

5. Average PM values per activity

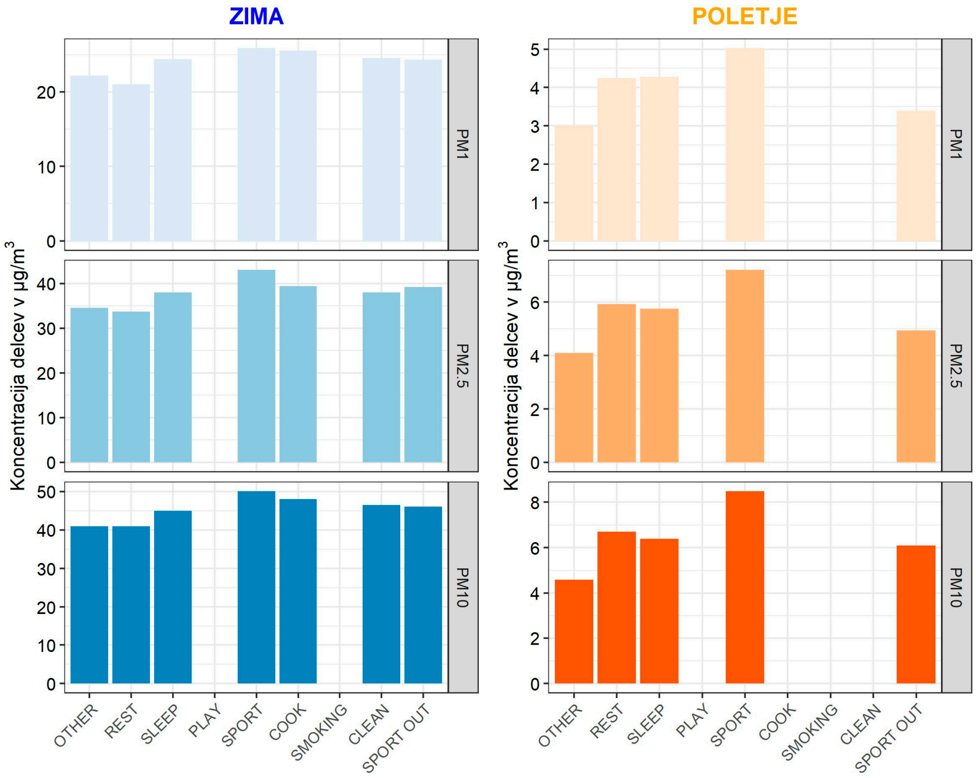

Figure 5. Average PM values per activity of the person during winter (left) and summer (right). Activities are marked in English: »Clean« – čiščenje, »Rest« - počivanje, »Smoking« - kajenje, »Cook« - kuhanje, »Sleep« - spanje, »Sport in« - športe aktivnosti v zaprtih prostorih, »Sport out« - športe aktivnosti zunaj, »Other« - drugo

Look at the Figure 5 and try to answer the following questions:

- During which activity were average PM10 concentrations the lowest? ________
- Given the selected method of displaying the measured values, is it possible to directly compare the values between winter and summer? YES NO
