# Supplementary material for: User-Centred Design of a Final Results Report for Participants in Multi-Sensor Personal Air Pollution Exposure Monitoring Campaigns
Source: Int J Environ Res Public Health. 2021 Nov 28;18(23):12544. doi: 10.3390/ijerph182312544 (PMC8656880; doi:10.3390/ijerph182312544)
Supplement: Supplementary file 1 [file ijerph-18-12544-s001.zip › S3_Focus_group_PPT.pptx]

## Slide 1
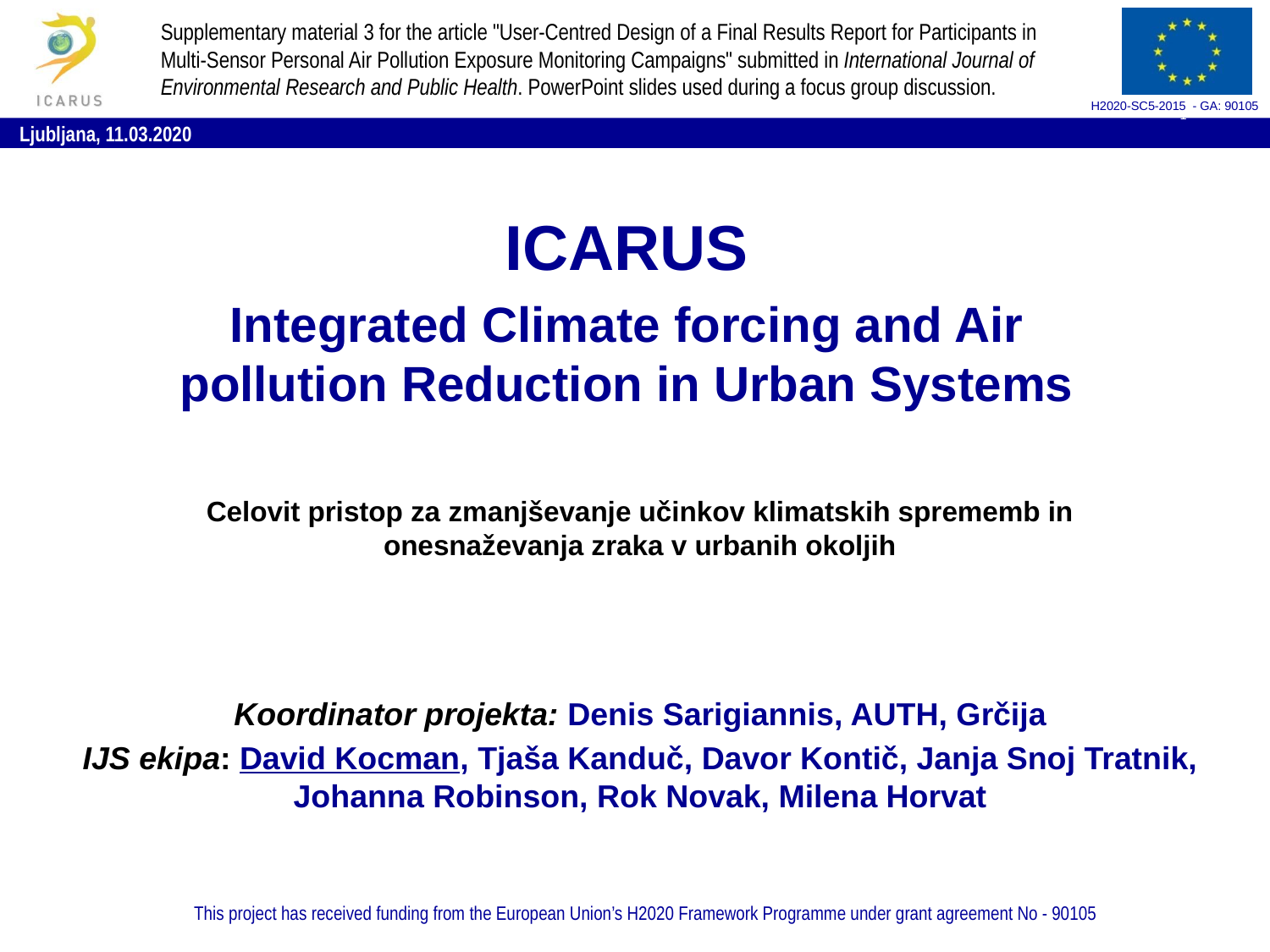

Supplementary material 3 for the article "User-Centred Design of a Final Results Report for Participants in Multi-Sensor Personal Air Pollution Exposure Monitoring Campaigns" submitted in International Journal of Environmental Research and Public Health. PowerPoint slides used during a focus group discussion.
ICARUS
Integrated Climate forcing and Air pollution Reduction in Urban Systems
Celovit pristop za zmanjševanje učinkov klimatskih sprememb in onesnaževanja zraka v urbanih okoljih
Koordinator projekta: Denis Sarigiannis, AUTH, Grčija
IJS ekipa: David Kocman, Tjaša Kanduč, Davor Kontič, Janja Snoj Tratnik, Johanna Robinson, Rok Novak, Milena Horvat

## Slide 2
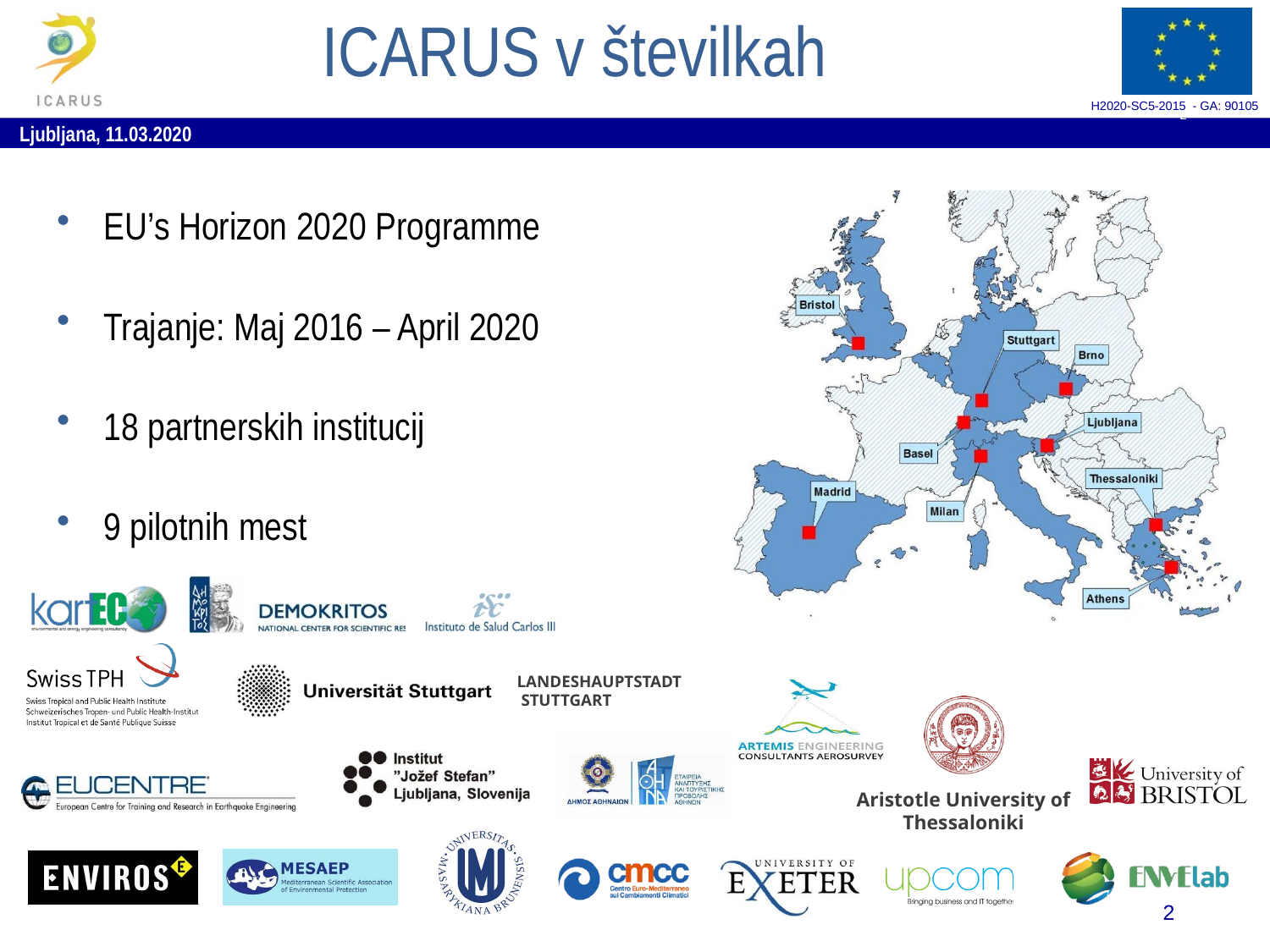

# ICARUS v številkah
EU’s Horizon 2020 Programme
Trajanje: Maj 2016 – April 2020
18 partnerskih institucij
9 pilotnih mest
LANDESHAUPTSTADT  STUTTGART
Aristotle University of Thessaloniki

## Slide 3
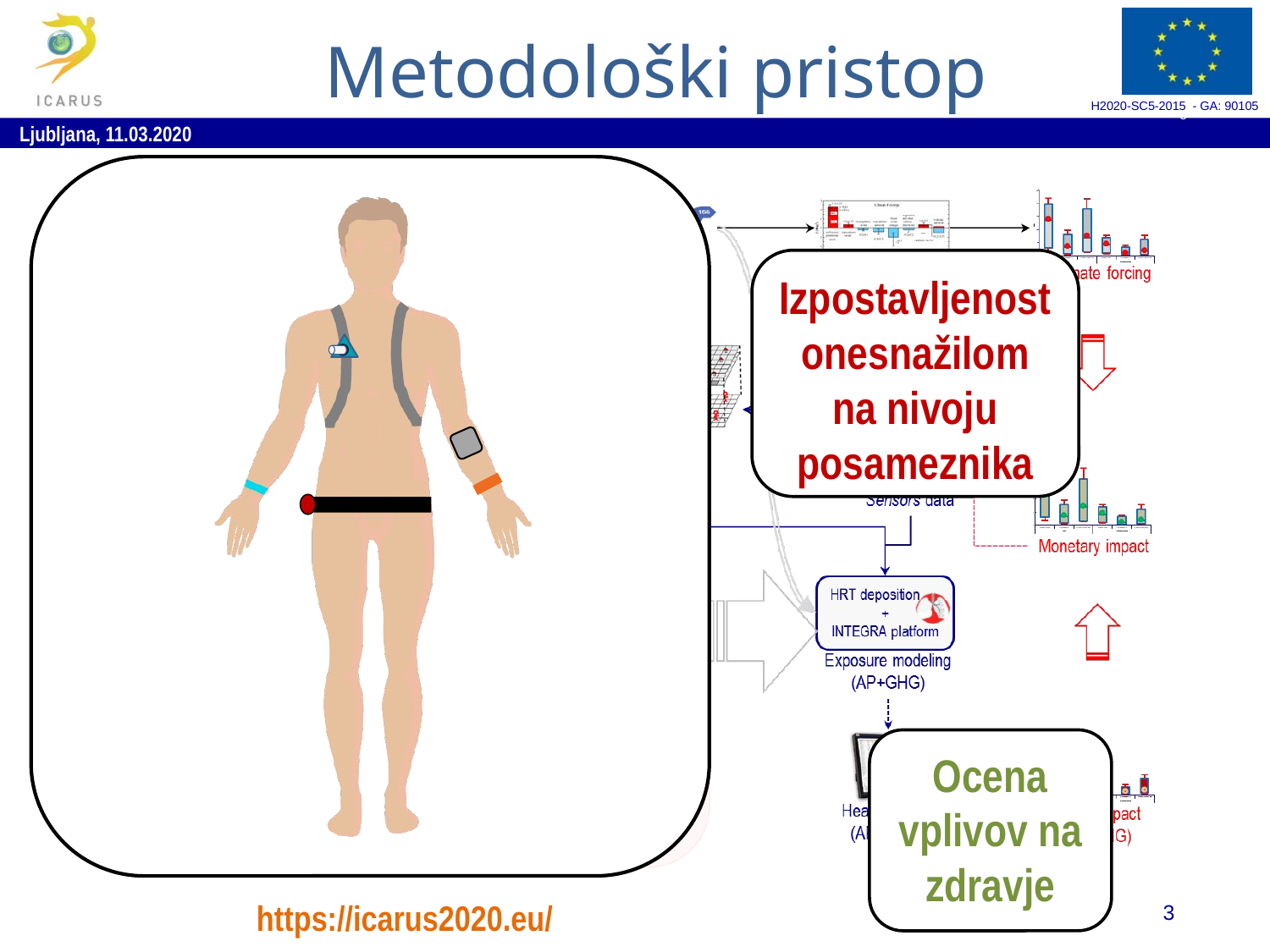

# Metodološki pristop
Meritve
Izpostavljenost
onesnažilom na nivoju posameznika
Emisije snovi v zrak
Modelni pristopi & analiza scenarijev
Ocena vplivov na zdravje
https://icarus2020.eu/

## Slide 4
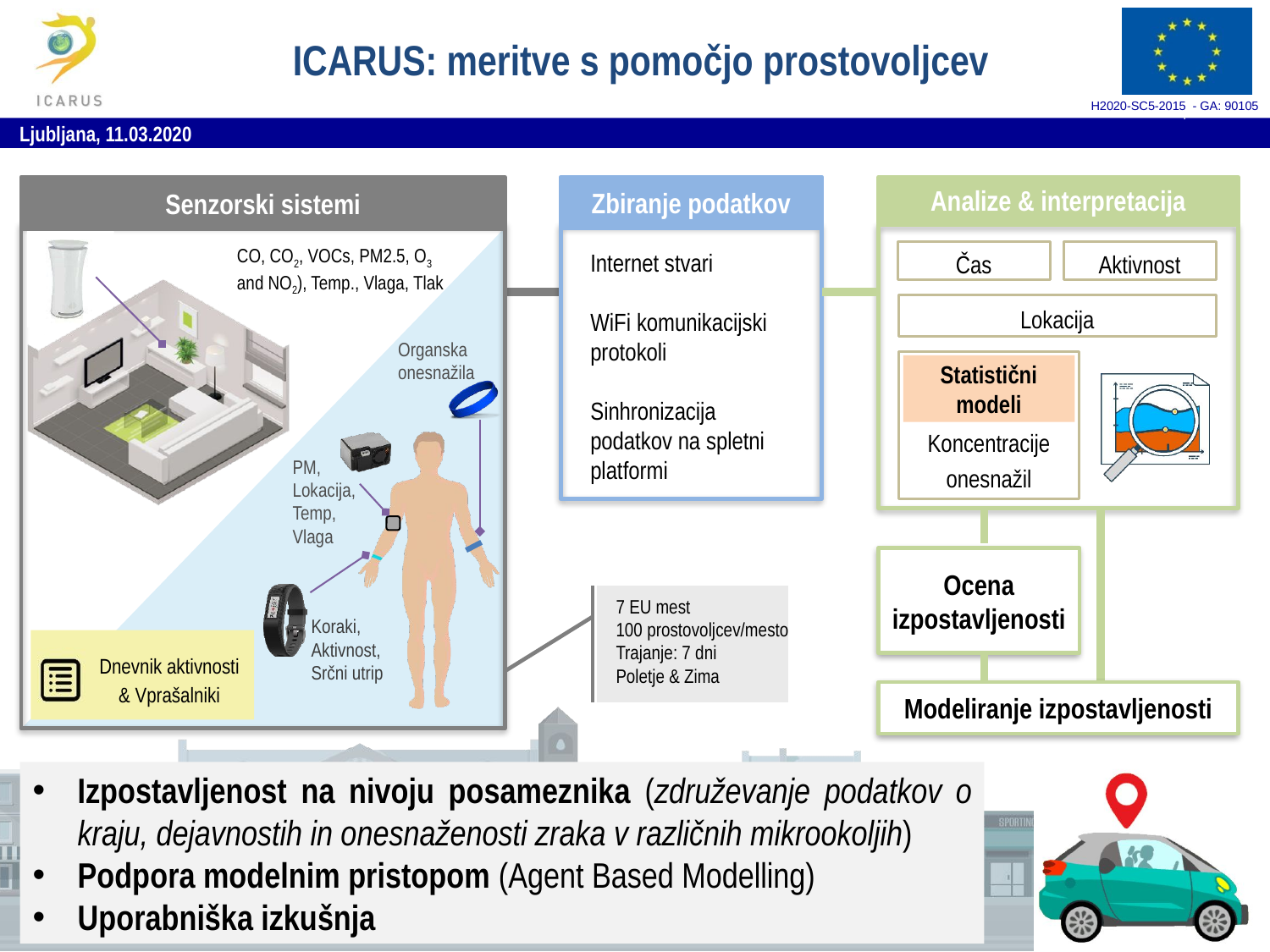

ICARUS: meritve s pomočjo prostovoljcev
Zbiranje podatkov
Senzorski sistemi
Analize & interpretacija
CO, CO2, VOCs, PM2.5, O3 and NO2), Temp., Vlaga, Tlak
Internet stvari
WiFi komunikacijski protokoli
Sinhronizacija podatkov na spletni platformi
Čas
Aktivnost
Lokacija
Organska onesnažila
Koncentracije onesnažil
Statistični modeli
PM, Lokacija,
Temp,
Vlaga
Ocena izpostavljenosti
7 EU mest
100 prostovoljcev/mesto
Trajanje: 7 dni
Poletje & Zima
Koraki,
Aktivnost,
Srčni utrip
Dnevnik aktivnosti
& Vprašalniki
Modeliranje izpostavljenosti
Izpostavljenost na nivoju posameznika (združevanje podatkov o kraju, dejavnostih in onesnaženosti zraka v različnih mikrookoljih)
Podpora modelnim pristopom (Agent Based Modelling)
Uporabniška izkušnja

## Slide 5
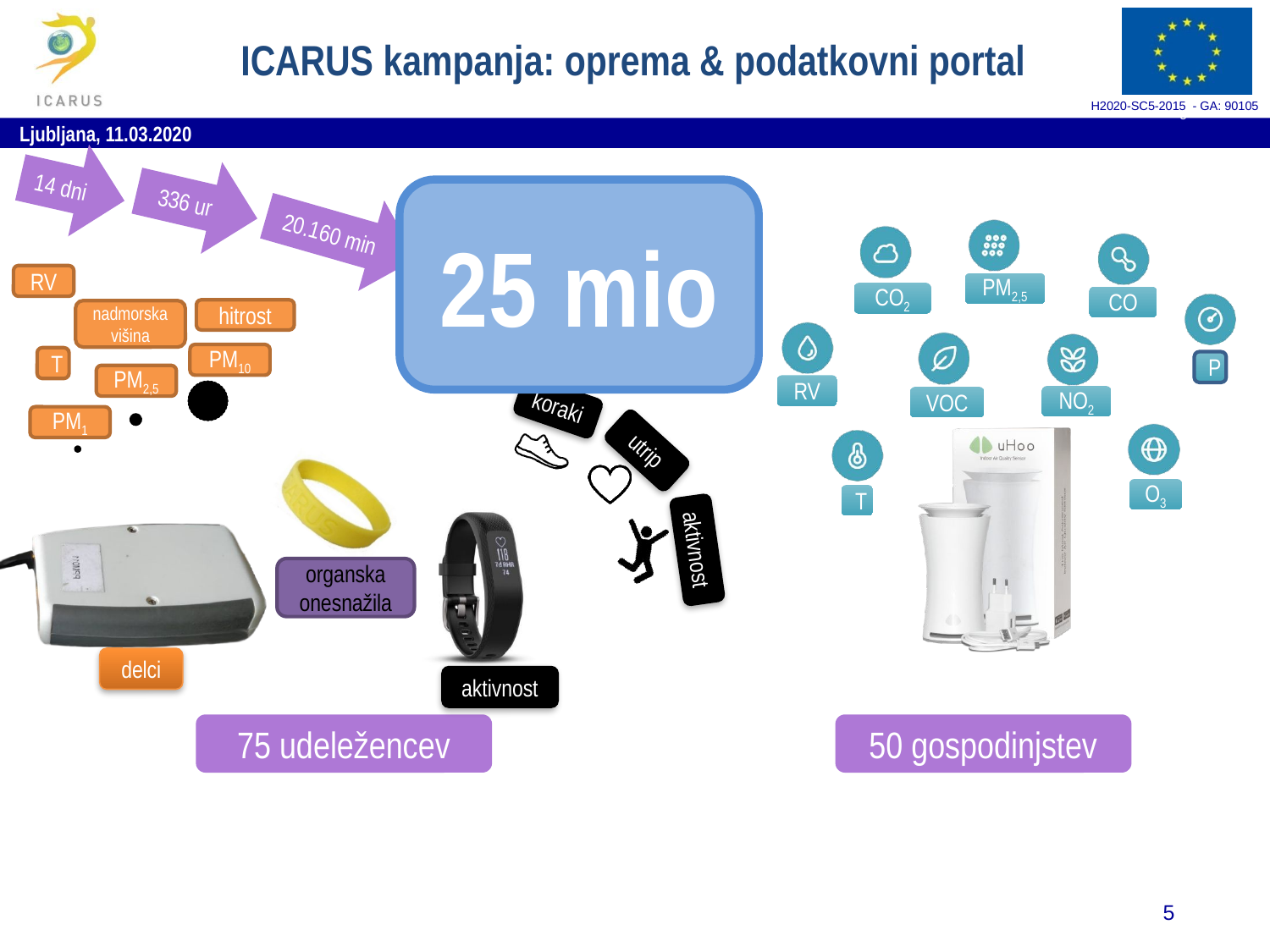

ICARUS kampanja: oprema & podatkovni portal
2 sezoni
14 dni
336 ur
25 mio
20.160 min
RV
PM2,5
CO2
CO
hitrost
nadmorska višina
PM10
T
P
PM2,5
RV
NO2
koraki
VOC
PM1
utrip
O3
T
aktivnost
organska onesnažila
delci
aktivnost
50 gospodinjstev
75 udeležencev

## Slide 6
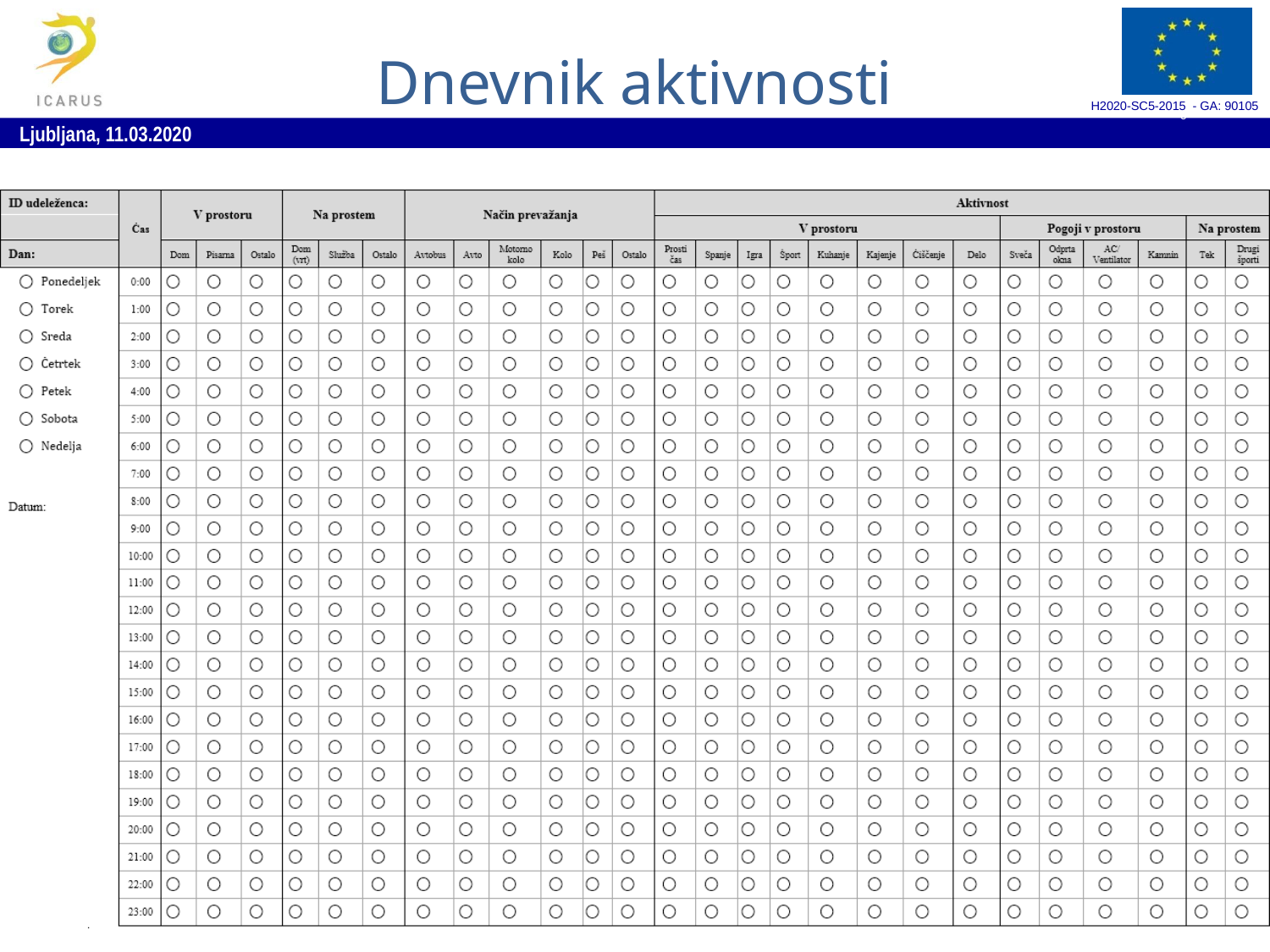

# Dnevnik aktivnosti

## Slide 7
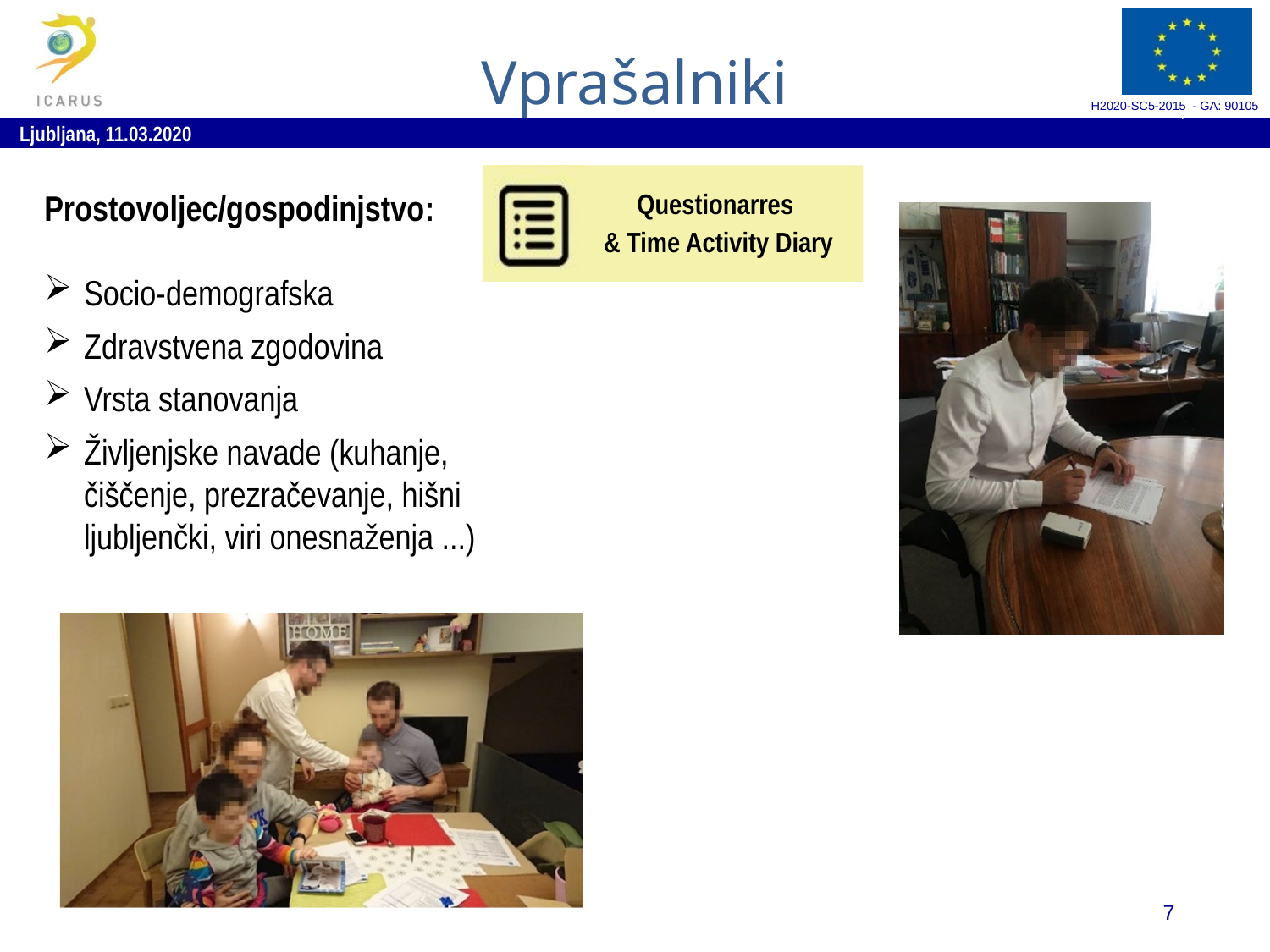

# Vprašalniki
Questionarres
& Time Activity Diary
Prostovoljec/gospodinjstvo:
Socio-demografska
Zdravstvena zgodovina
Vrsta stanovanja
Življenjske navade (kuhanje, čiščenje, prezračevanje, hišni ljubljenčki, viri onesnaženja ...)

## Slide 8
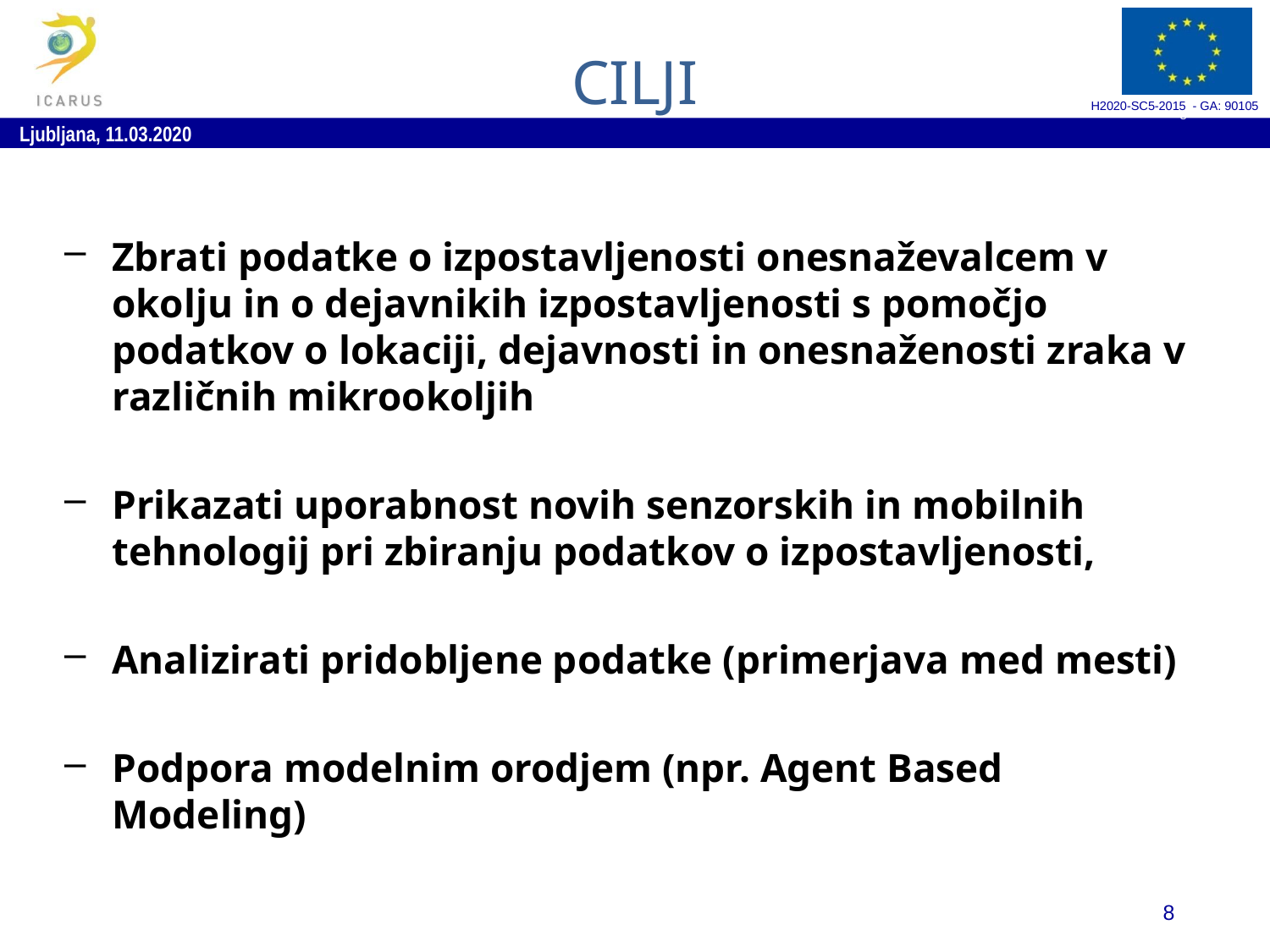

# CILJI
Zbrati podatke o izpostavljenosti onesnaževalcem v okolju in o dejavnikih izpostavljenosti s pomočjo podatkov o lokaciji, dejavnosti in onesnaženosti zraka v različnih mikrookoljih
Prikazati uporabnost novih senzorskih in mobilnih tehnologij pri zbiranju podatkov o izpostavljenosti,
Analizirati pridobljene podatke (primerjava med mesti)
Podpora modelnim orodjem (npr. Agent Based Modeling)

## Slide 9
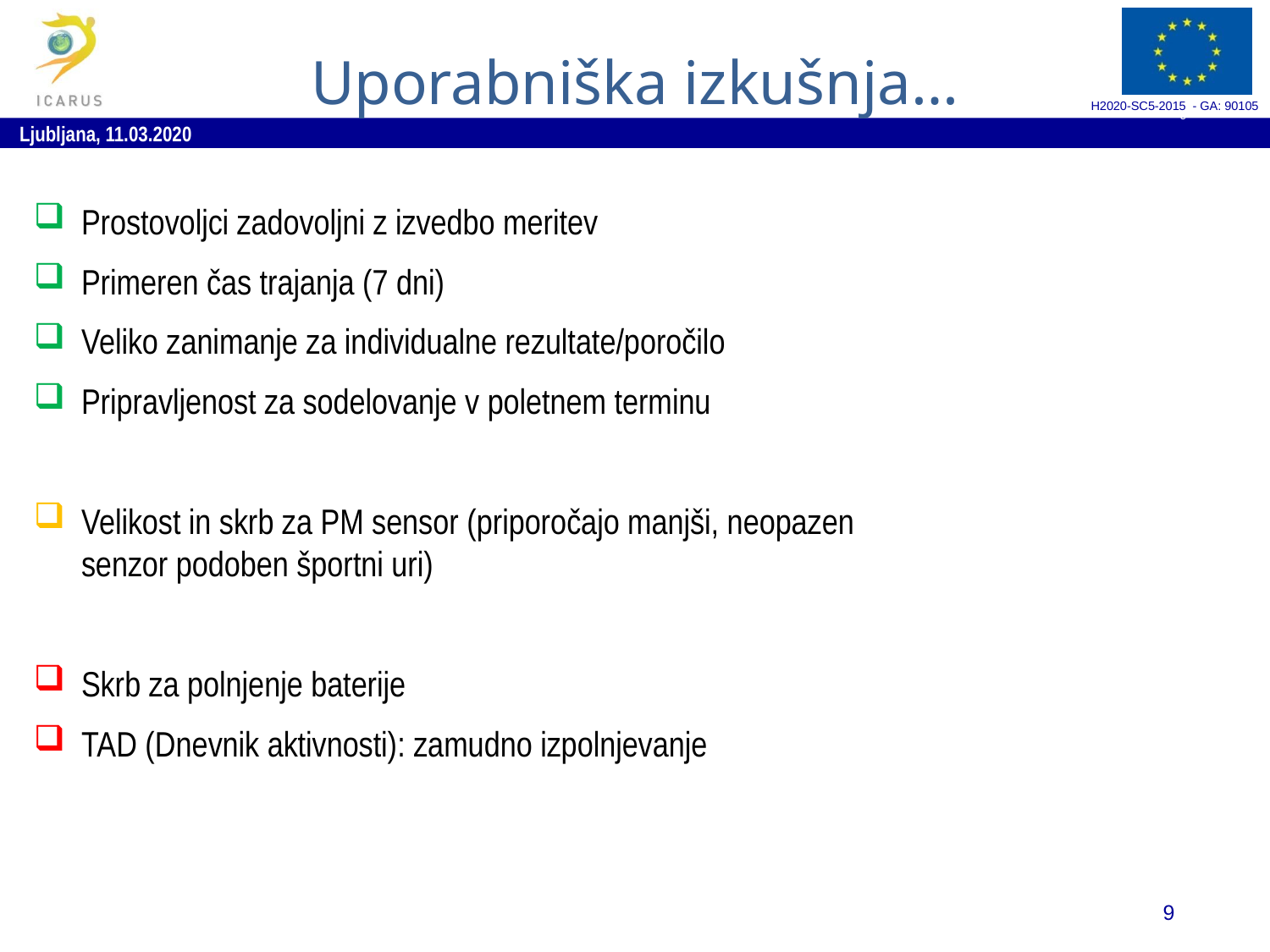

# Uporabniška izkušnja…
Prostovoljci zadovoljni z izvedbo meritev
Primeren čas trajanja (7 dni)
Veliko zanimanje za individualne rezultate/poročilo
Pripravljenost za sodelovanje v poletnem terminu
Velikost in skrb za PM sensor (priporočajo manjši, neopazen senzor podoben športni uri)
Skrb za polnjenje baterije
TAD (Dnevnik aktivnosti): zamudno izpolnjevanje

## Slide 10
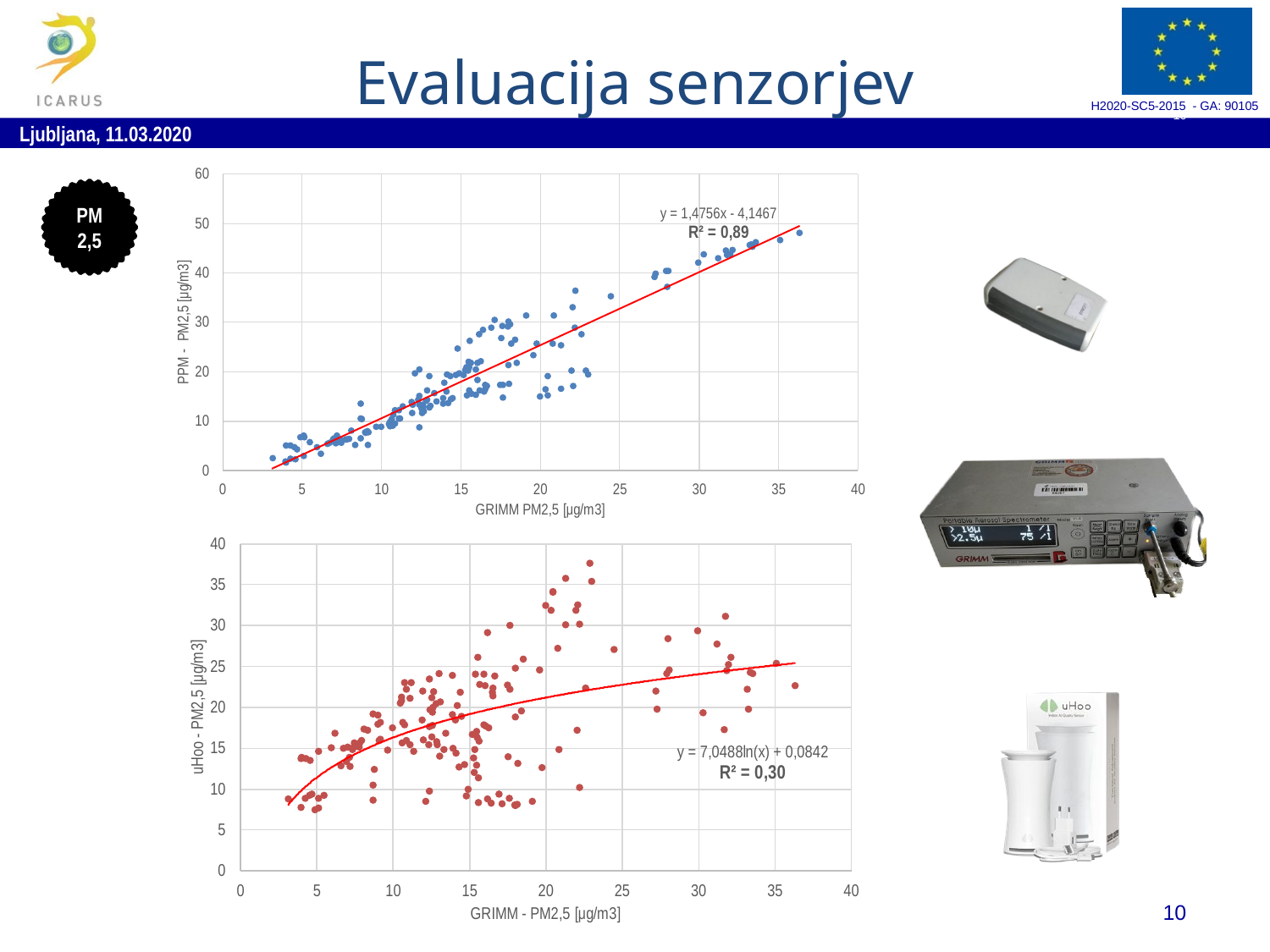

# Evaluacija senzorjev
PM
2,5

## Slide 11
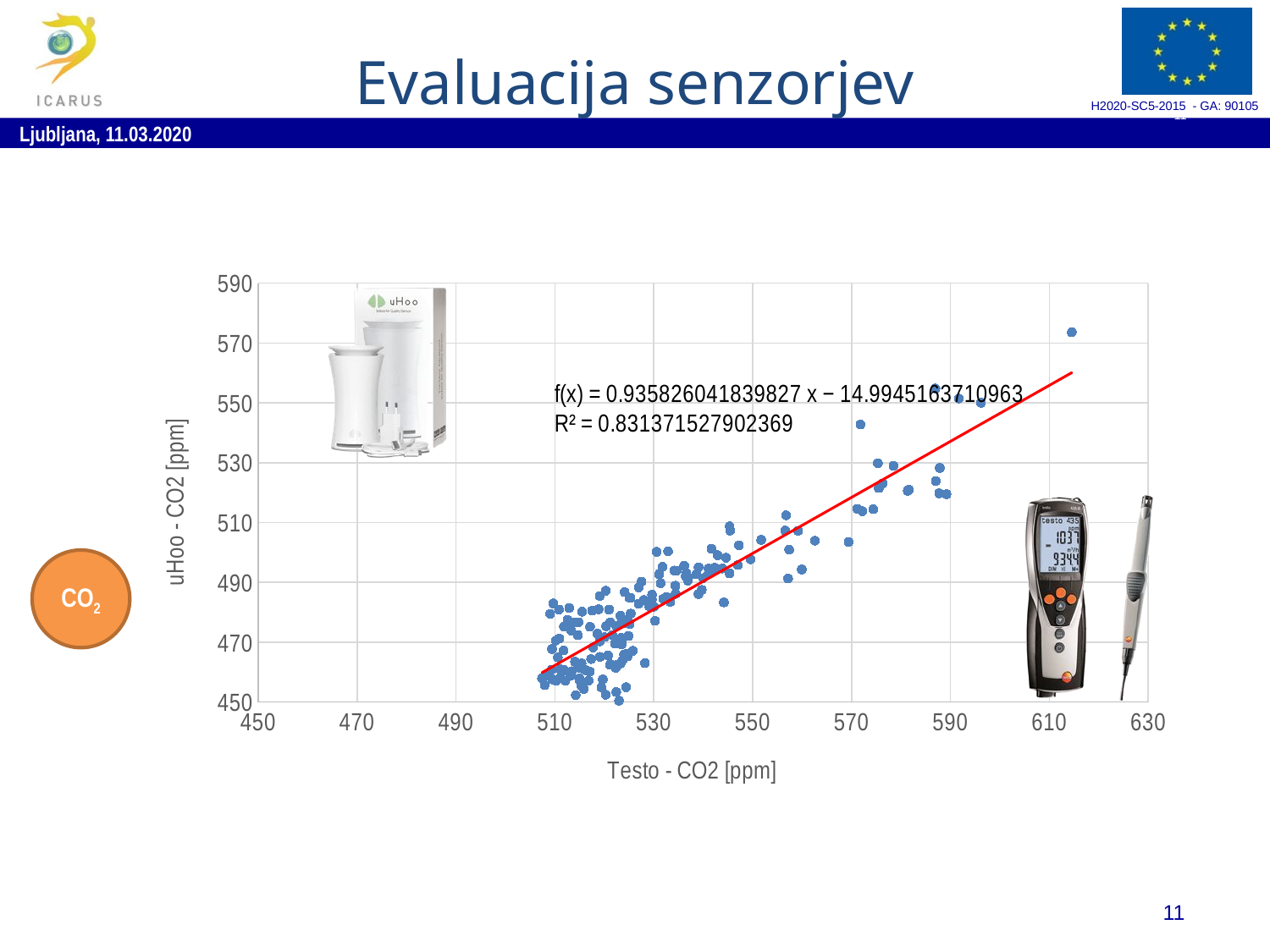

# Evaluacija senzorjev
### Chart
| Category | CO2 (uHoo) |
|---|---|
CO2

## Slide 12
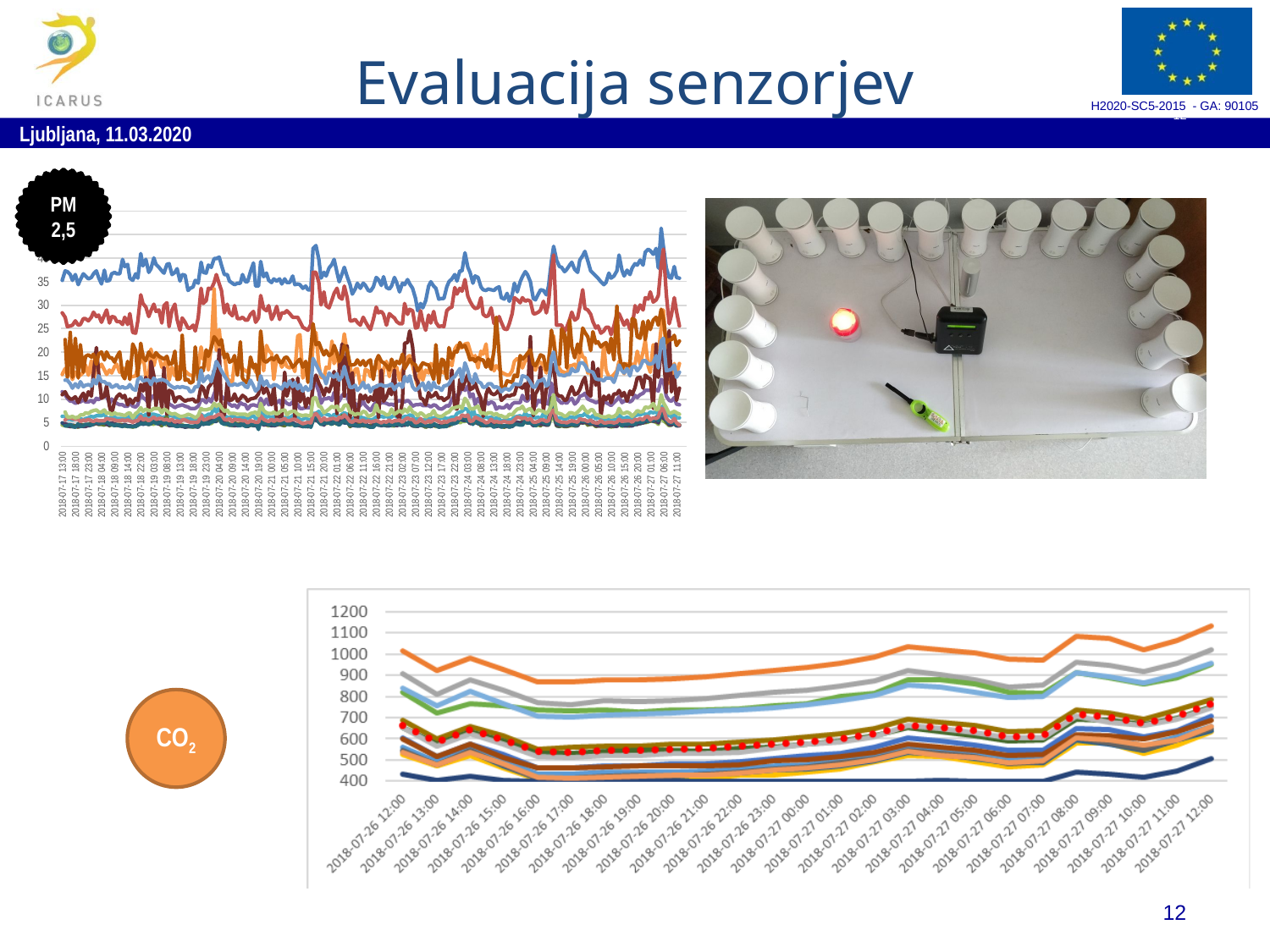

# Evaluacija senzorjev
PM
2,5
CO2

## Slide 13
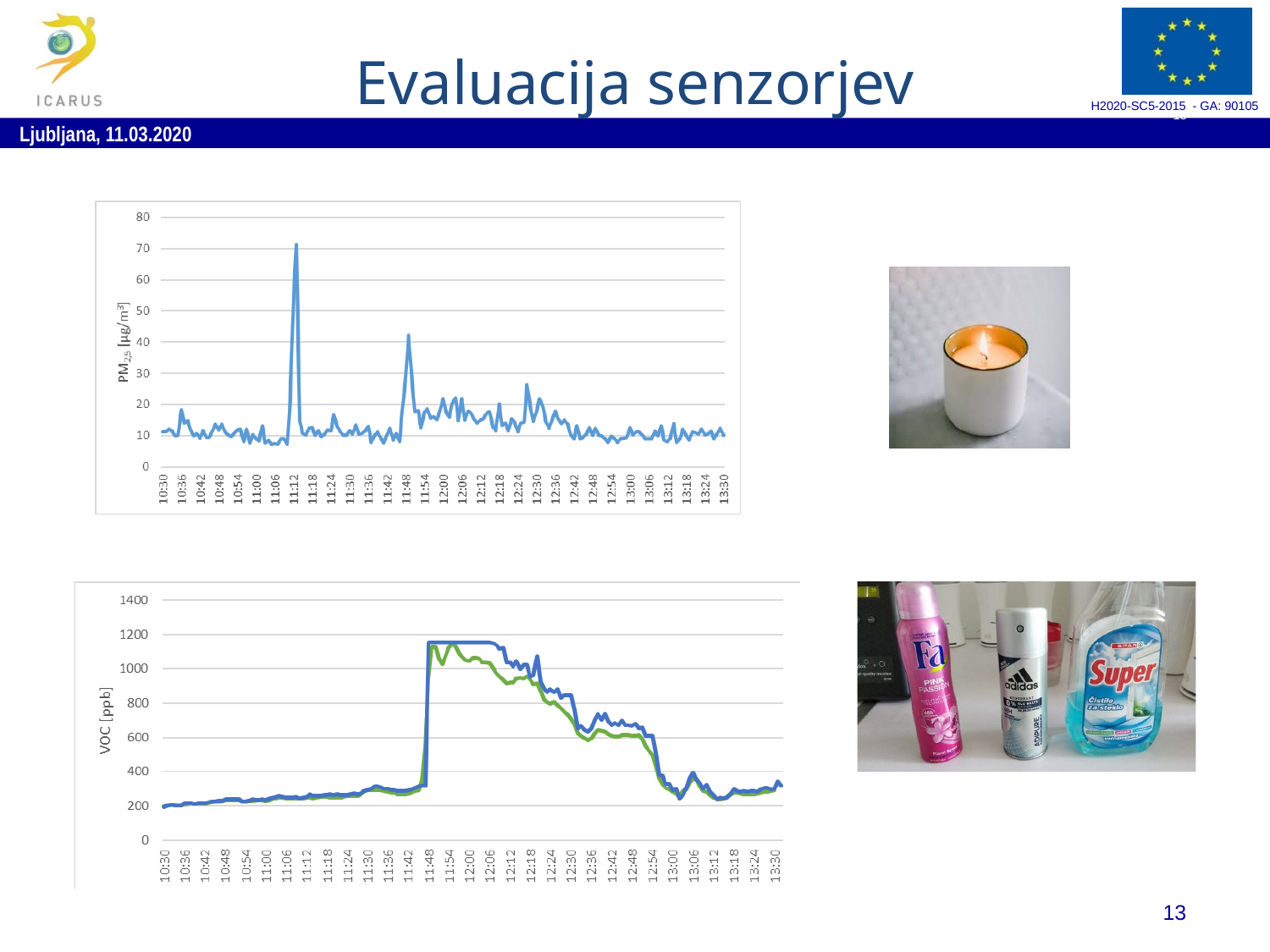

# Evaluacija senzorjev

## Slide 14
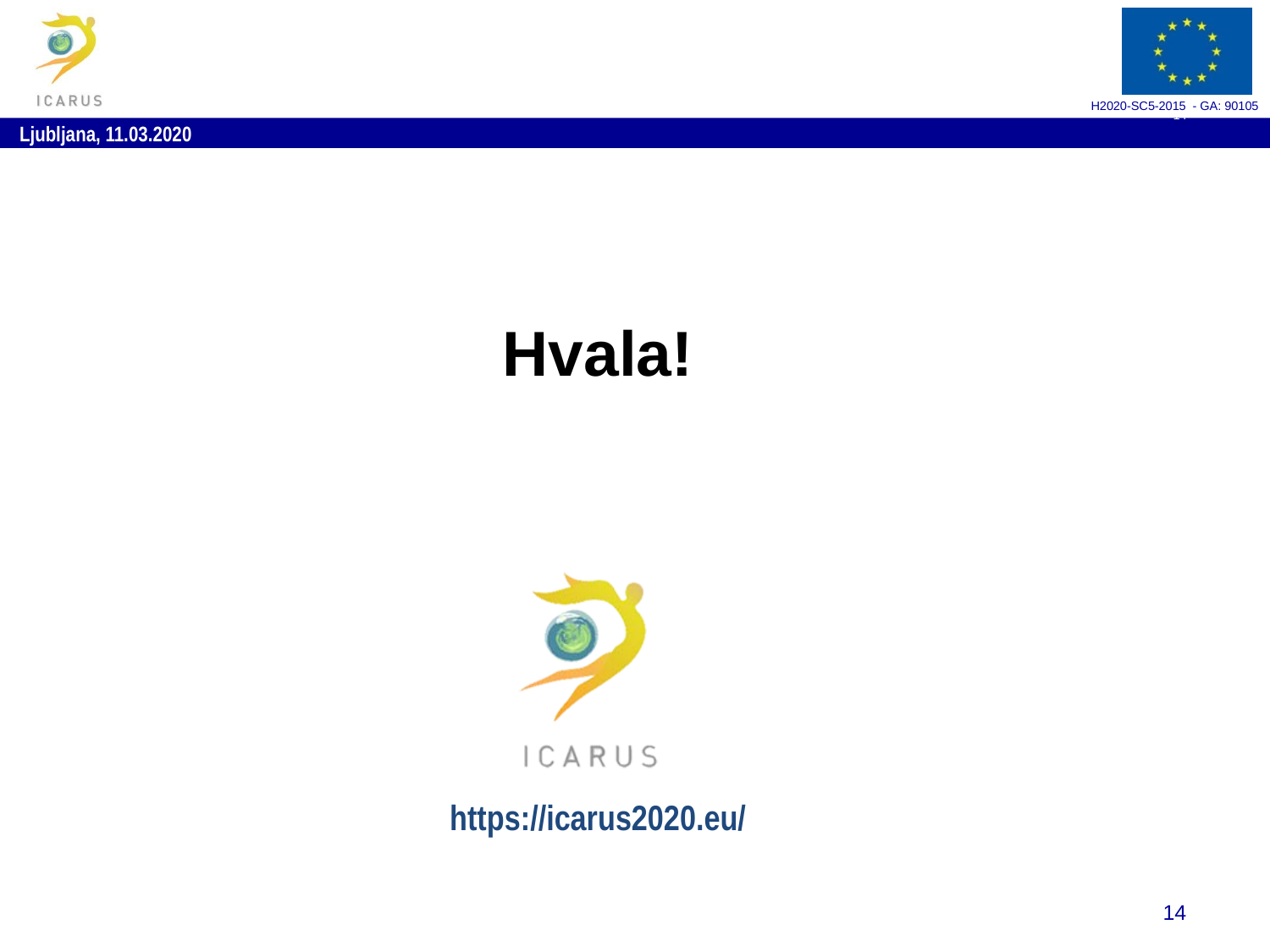

Hvala!
https://icarus2020.eu/

## Slide 15
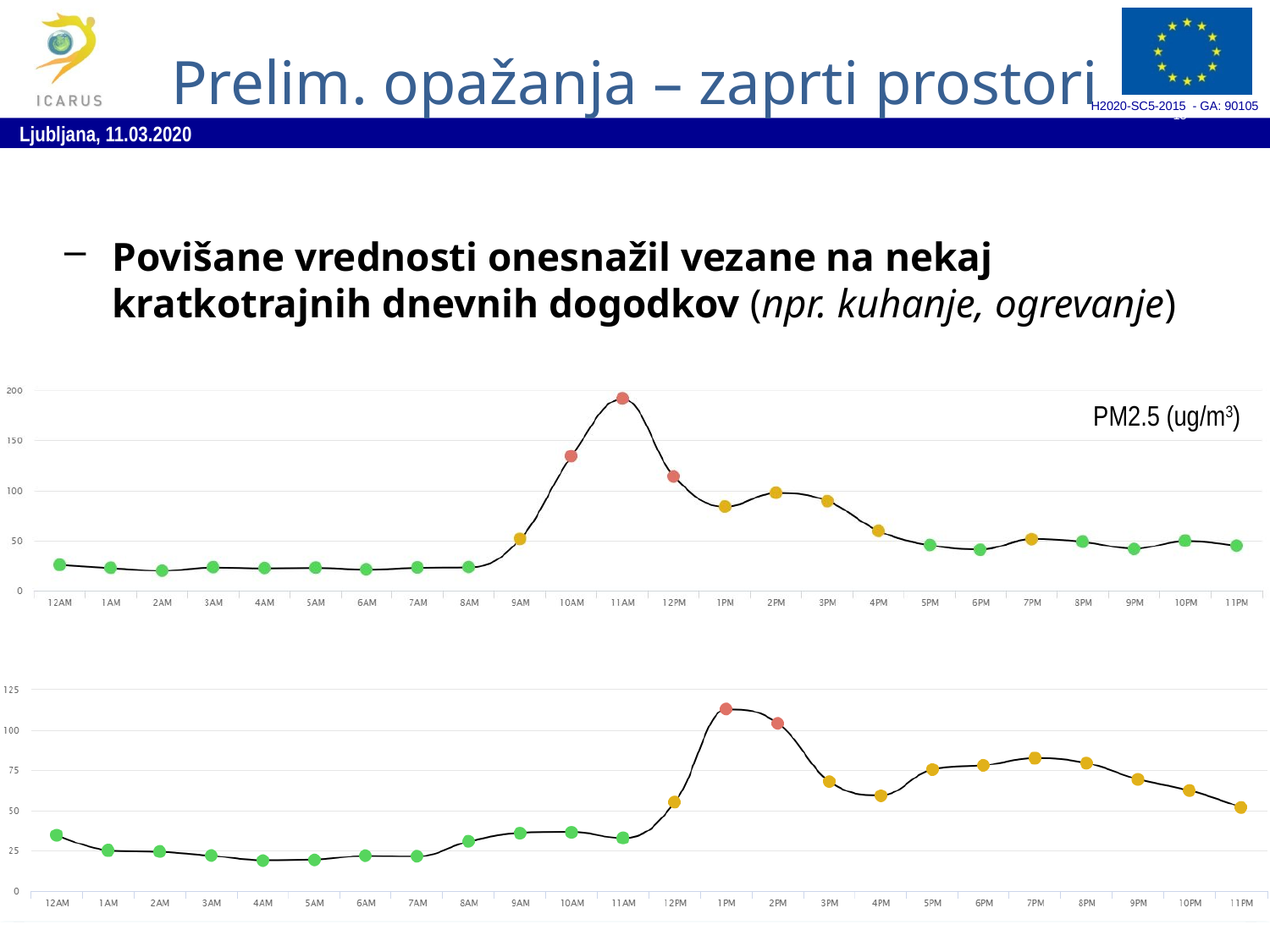

# Prelim. opažanja – zaprti prostori
Povišane vrednosti onesnažil vezane na nekaj kratkotrajnih dnevnih dogodkov (npr. kuhanje, ogrevanje)
PM2.5 (ug/m3)

## Slide 16
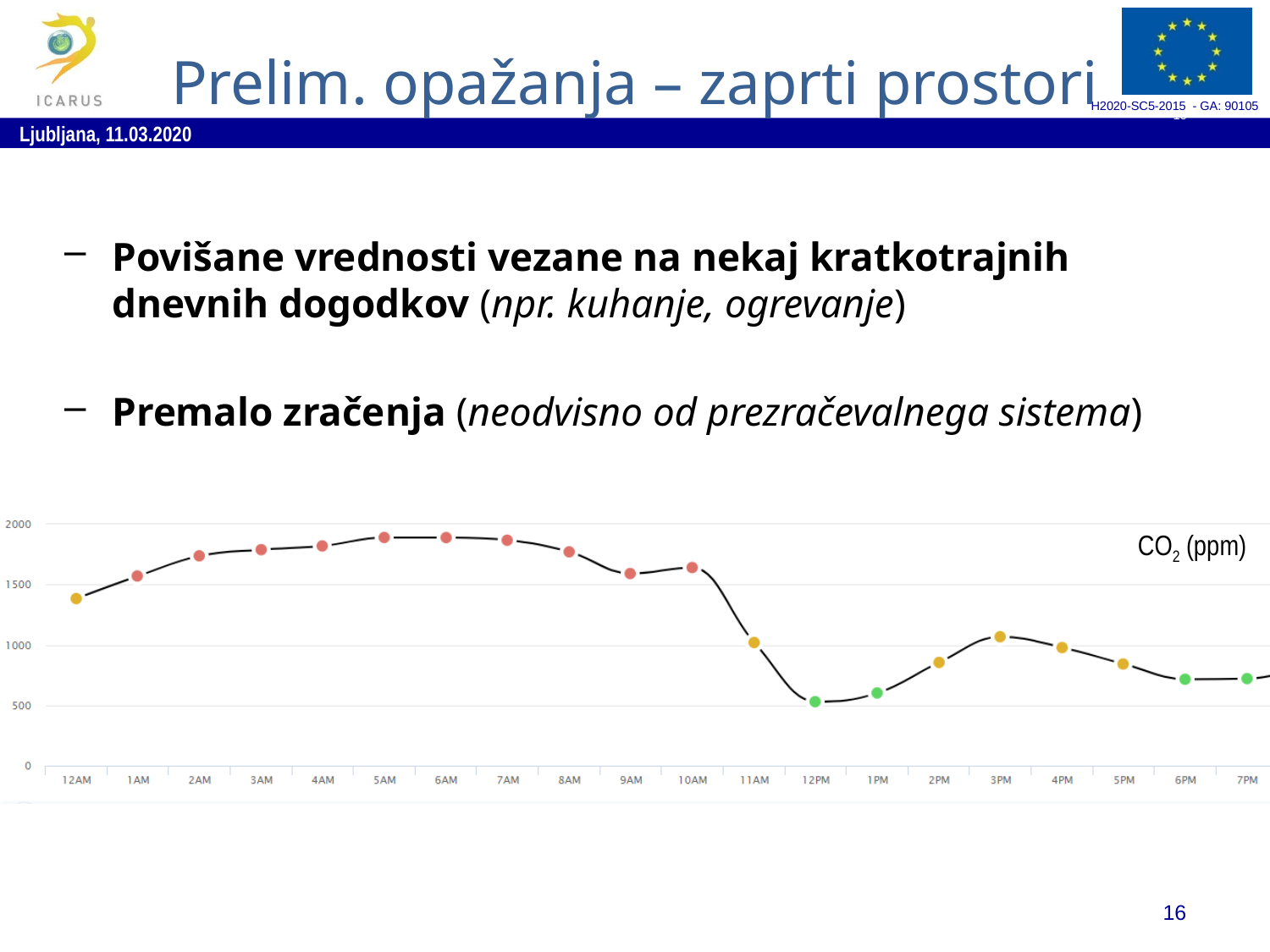

# Prelim. opažanja – zaprti prostori
Povišane vrednosti vezane na nekaj kratkotrajnih dnevnih dogodkov (npr. kuhanje, ogrevanje)
Premalo zračenja (neodvisno od prezračevalnega sistema)
CO2 (ppm)

## Slide 17
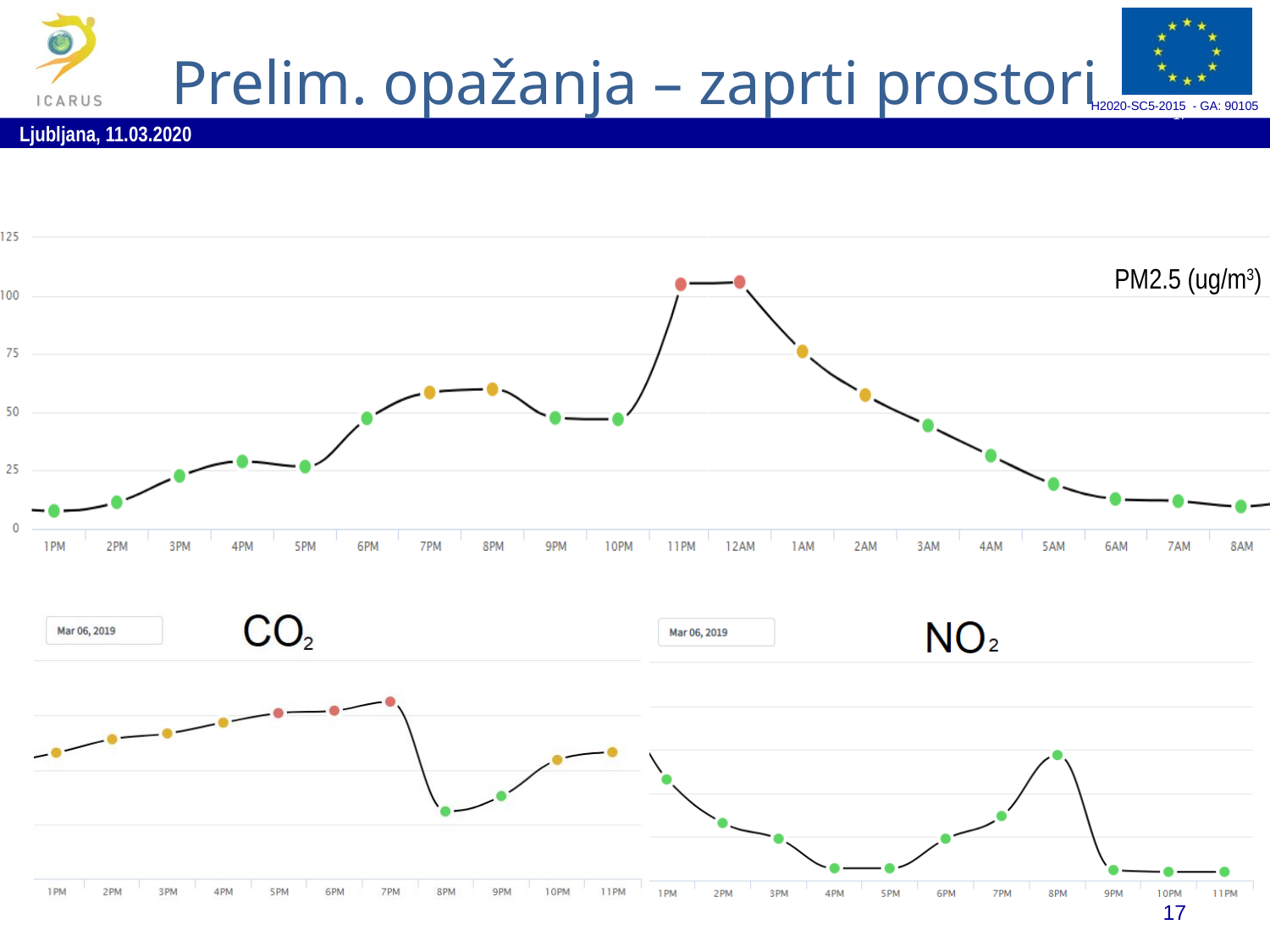

# Prelim. opažanja – zaprti prostori
Povišane vrednosti vezane na nekaj kratkotrajnih dnevnih dogodkov (npr. kuhanje, ogrevanje)
Premalo zračenja (neodvisno od prezračevalnega sistema)
Čas in trajanje zračenja (odvisno od aktivnosti in lokacije)
PM2.5 (ug/m3)

## Slide 18
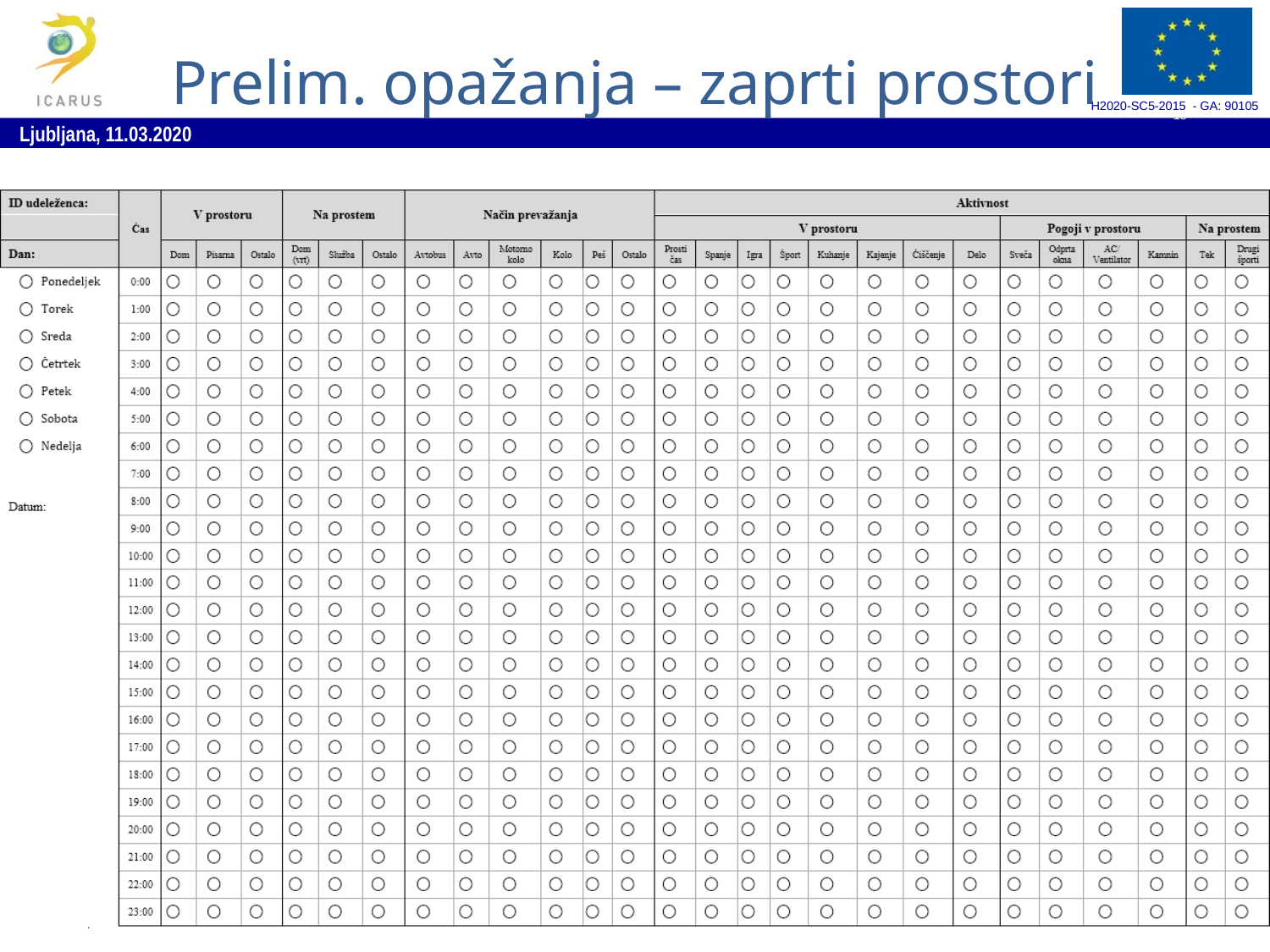

# Prelim. opažanja – zaprti prostori
Povišane vrednosti vezane na nekaj kratkotrajnih dnevnih dogodkov (npr. kuhanje, ogrevanje)
Premalo zračenja (neodvisno od prezračevalnega sistema)
Čas in trajanje zračenja (odvisno od aktivnosti in lokacije)
Za ustrezno interpretacijo nujno poznavanje aktivnosti v prostoru (Time-Activity Diaries)

## Slide 19
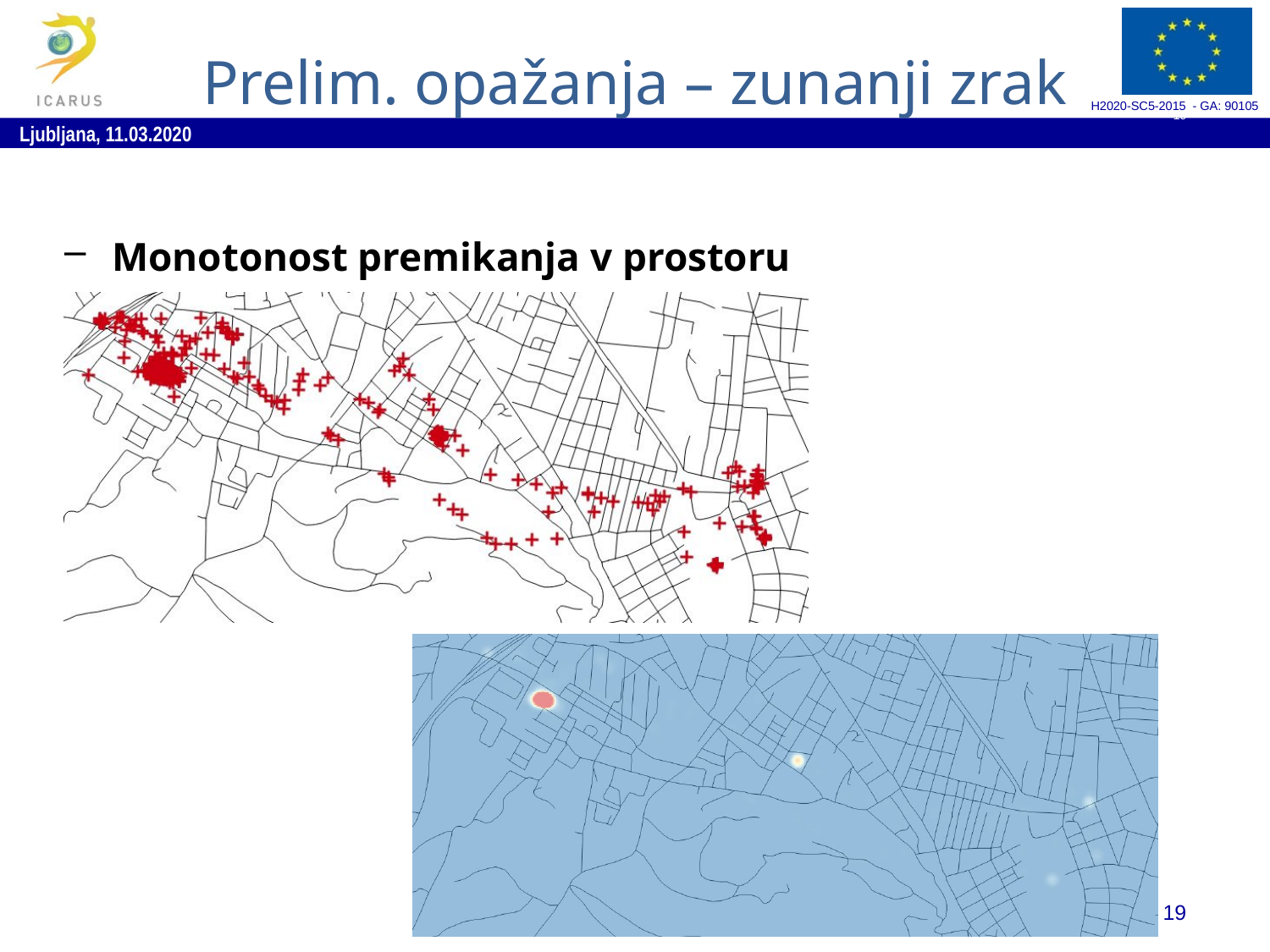

# Prelim. opažanja – zunanji zrak
Monotonost premikanja v prostoru

## Slide 20
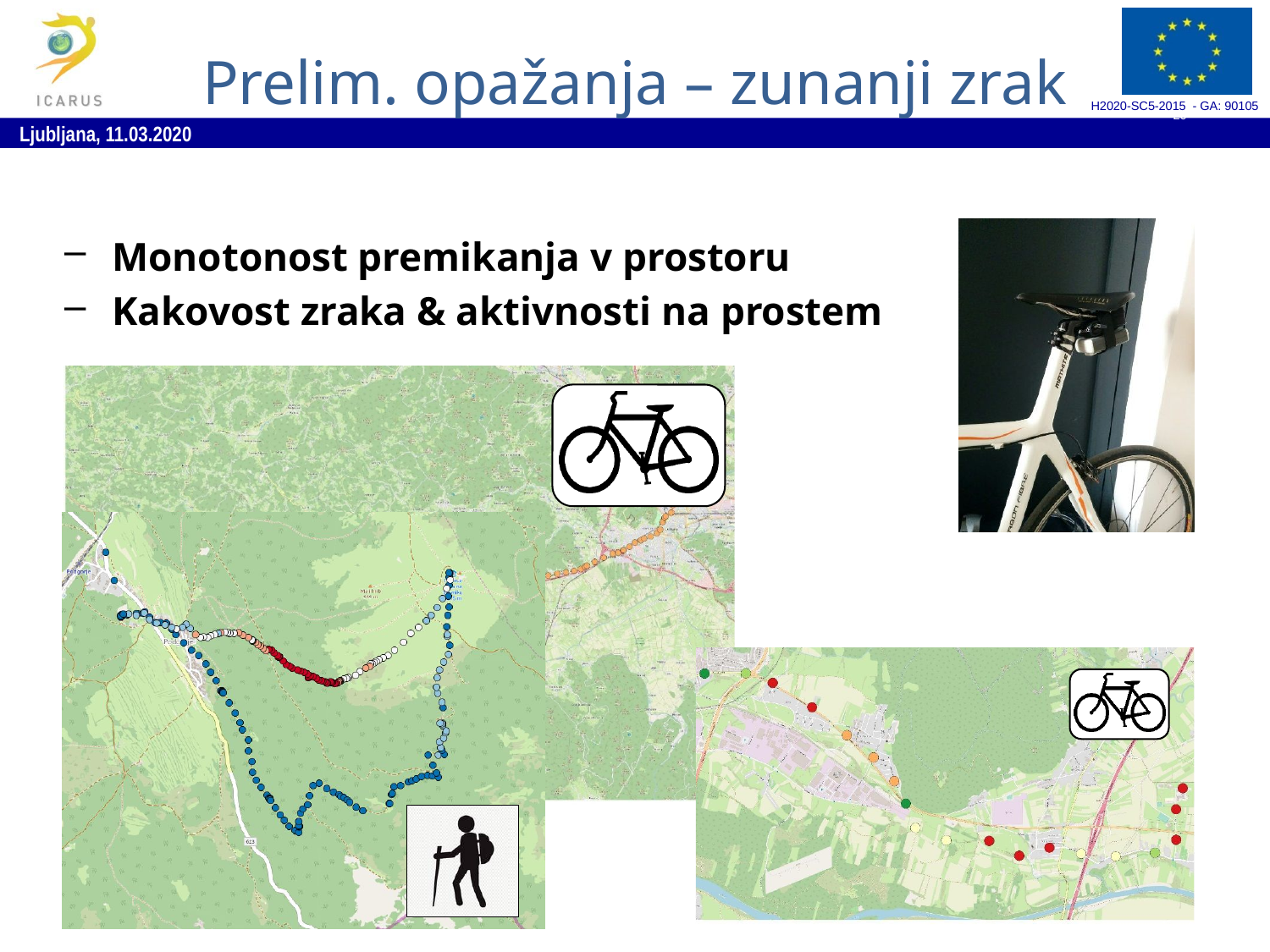

# Prelim. opažanja – zunanji zrak
Monotonost premikanja v prostoru
Kakovost zraka & aktivnosti na prostem

## Slide 21
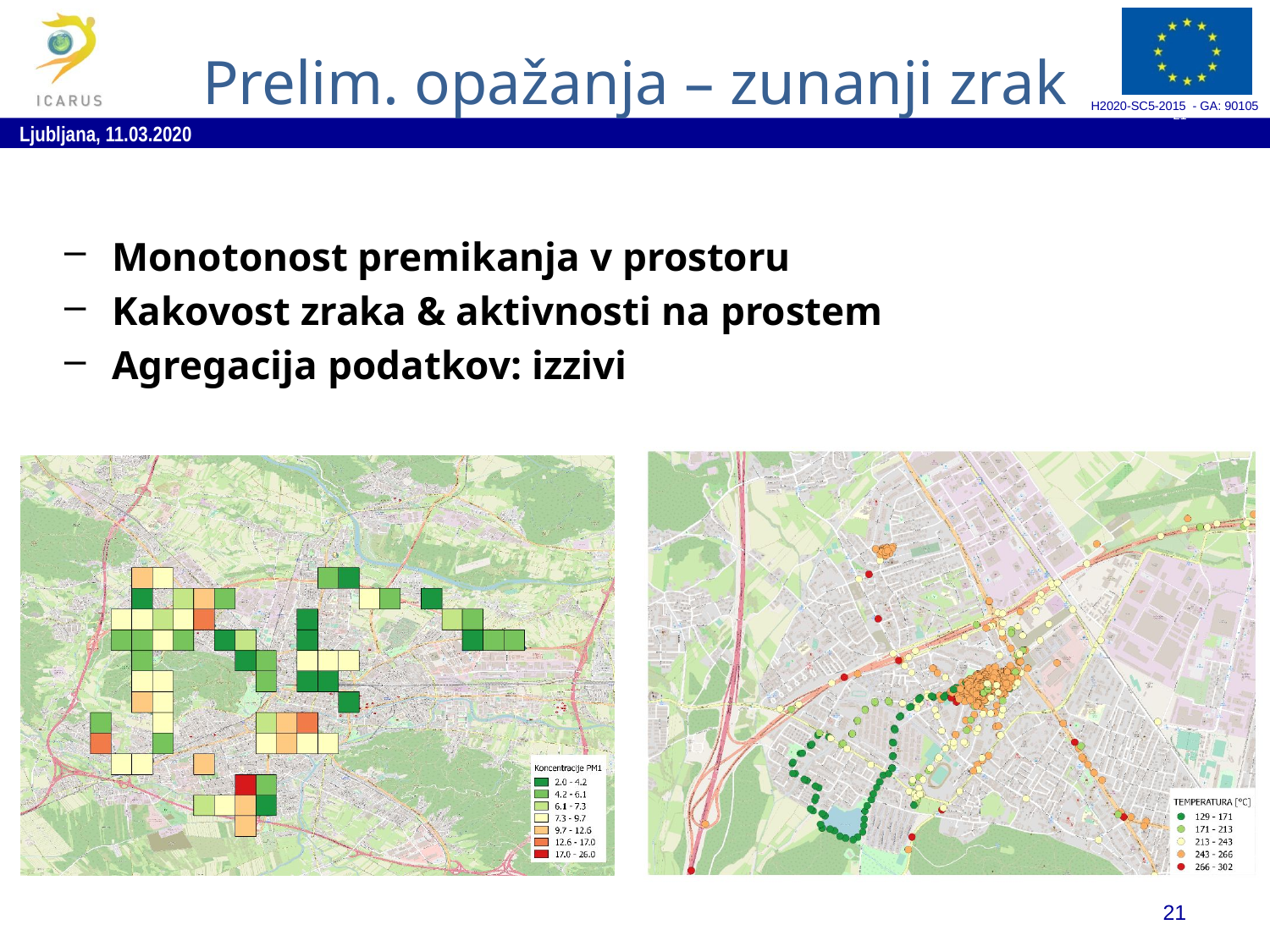

# Prelim. opažanja – zunanji zrak
Monotonost premikanja v prostoru
Kakovost zraka & aktivnosti na prostem
Agregacija podatkov: izzivi

## Slide 22
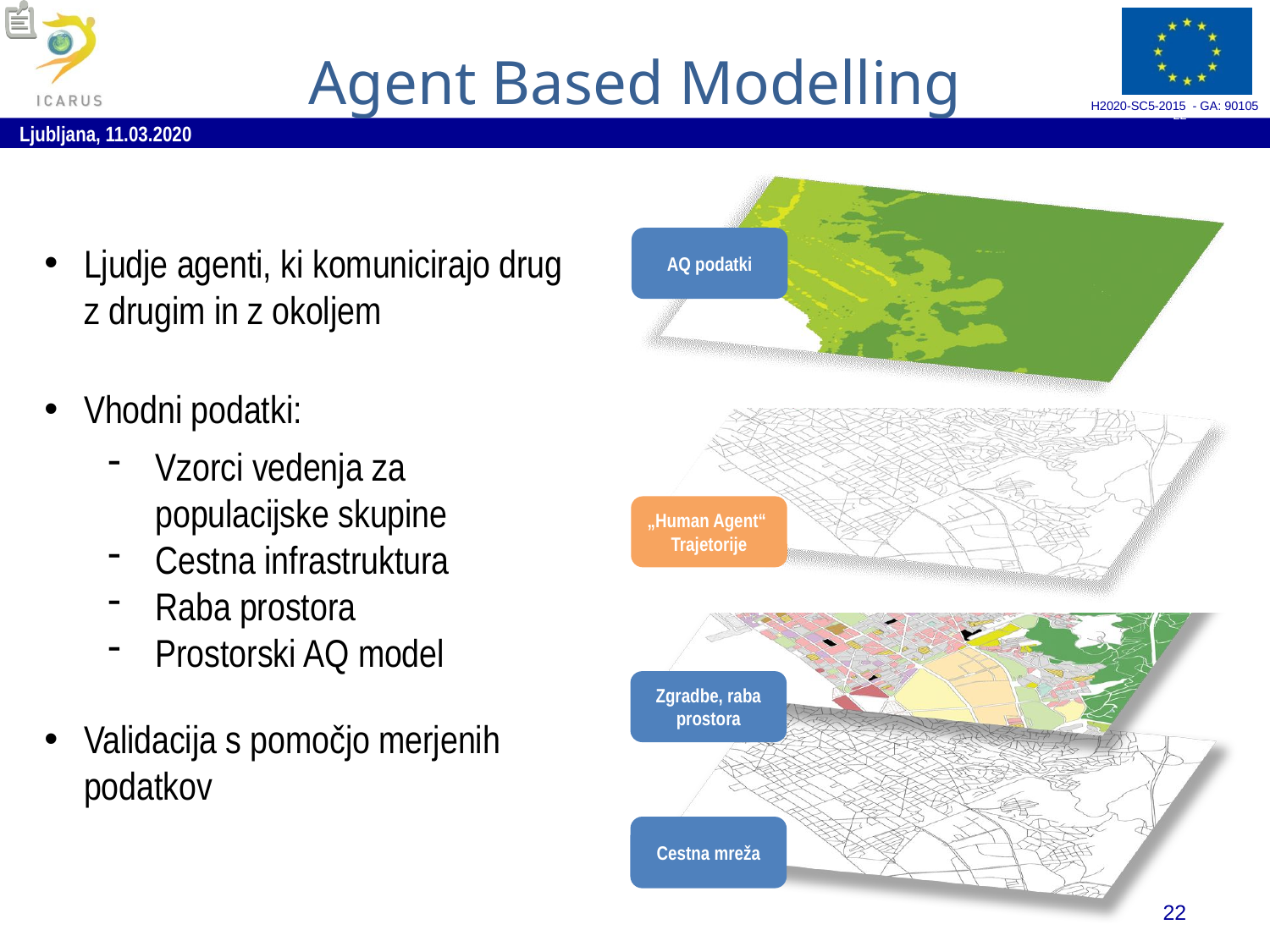

# Agent Based Modelling
AQ podatki
Ljudje agenti, ki komunicirajo drug z drugim in z okoljem
Vhodni podatki:
Vzorci vedenja za populacijske skupine
Cestna infrastruktura
Raba prostora
Prostorski AQ model
Validacija s pomočjo merjenih podatkov
„Human Agent“ Trajetorije
Zgradbe, raba prostora
Cestna mreža
