# Supplementary material for: User-Centred Design of a Final Results Report for Participants in Multi-Sensor Personal Air Pollution Exposure Monitoring Campaigns
Source: Int J Environ Res Public Health. 2021 Nov 28;18(23):12544. doi: 10.3390/ijerph182312544 (PMC8656880; doi:10.3390/ijerph182312544)
Supplement: Supplementary file 1 [file ijerph-18-12544-s001.zip › S4_transcript_and_summary_of_changes.pdf]

Supplementary material 2 for the article "User-Centred Design of a Final Results Report for Participants in Multi-Sensor Personal Air Pollution Exposure Monitoring Campaigns" submitted in *International Journal of Environmental Research and Public Health* consisting of:

1. Instructions given to a student writing the transcript

2. Summary of further actions

3. Transcript and translation of the focus group

4. List of suggestions from the participants and actions taken based on them

1. Instructions given to a student writing the transcript:

- Label the participants: Researcher 1=R1, Researcher 2=R2, Researcher 3 = R3, Female participant 1= FP1, Male participant 1= MP1, Male participant 2= MP2…
- When writing down the recording try to pay attention to who is talking, use encripted participant IDs as above. Add as many participants as you hear.
- The transcript should look something like this:
  - R1: Bla bla bla
  - R2: Bla bla bla
  - FP1: Bla bla bla
  - R1: Bla bla bla
  - FP2: Bla bla bla
- If you want you can add time every now and then, so it is easier to go back to look who said what. No need to add it every time someone different talks. i.e:
  - **05:20**
  - R1: Bla bla bla
- When you need to re-hear what someone said, press Shift+left arrow for going back 3 seconds, or Alt+left arrow for 10 seconds (at least in VLC).
- In case you don't hear properly what someone said, mark it red. i.e. when two people are talkign at the same time it might be hard to distinguish certain words.
- After you have finished transcription, please translate it in English.

2. Summary of further actions

The transcriptions were further supplemented with sections and additional information in [] and the textual corpus was read and corrected contextually and grammatically, thus following the realistic flow of the person speaking. Feedback from participants was summarised for the manuscript and specific actions can be found at the end of this supplementary material.

3. Transcript and translation of the focus group

Section 1: Welcome and short survey

Slovene (original language)	English (translated from Slovene)
**00:15**<br><br>R1: Preden vas mi kontaminiramo s kakršnimi koli informacijami, bi vas prosili, če izpolnite čisto kratek vprašalnik s katerim želimo preveriti koliko se vi spomnite, kaj ste v resnici počeli takrat, ko ste bili tu z nami.<br><br>FP3: Se pravi odgovori niso vsi pravilni.<br><br>R2: Ne.<br><br>(nekdo začne obračati liste)<br><br>**01:05**<br><br>R1: Samo na prvi strani je. Ni treba nič dvigovati listov, ker so še ene druge stvari za naprej tam.<br><br>**01:25**<br><br>FP2: Bi mogli povedat, da bi mi malo ponovili.<br><br>R1: To sledi z moje strani.<br><br>FP2: A moramo zdaj napisat kaj so bili cilji kampanje ali lahko potem?<br><br>R1: Kar tako, kot se vam zdi. Iz glave.<br><br>**02:35** Ali če hočete svoje inicialke gor še do pisat.	**00:15**<br><br>R1: Before we contaminate you with any information, we would ask you to fill out a brief questionnaire, to check how much you remember what you did when you were here with us. [Participants are given printed material, page 1]<br><br>FP3: So, answers are not all correct.<br><br>R2: No.<br><br>(someone probably starts turning sheets)<br><br>**01:05**<br><br>R1: It's only on the first page. There is no need to lift the sheets because there are other things listed there.<br><br>**01:25**<br><br>FP2: You could have told us, so we would have rehersed a little.<br><br>R1: That follows on my part.<br><br>FP2: But do we have to write down what the goals of the campaign were now, or can we do this later?<br><br>R1: Just as you see it. From top of your head.<br><br>**02:35** If you wish, you could write your initials in the paper.

Section 2: Introductionary Powerpoint presentation

**04:10**<br><br>R1: Zgleda da smo. Evo, sledi par diapozitivov v	**04:10**<br><br>R1: Looks like we are done. Here's a few slides to

katerem vas opominjamo na to kaj smo počeli in tu so odgovori na vprašanja, ki ste jih ravnokar reševali. Se pravi, bili ste udeleženi v tako imenovanem projektu ICARUS, ki je eden večjih Horizon 2020 projekt, ki se aprila letos zaključuje. Dogajal se je, ali odvijal, v devetih mestih prikazanih na karti, Ljubljana je bila ena izmed pilotnih mest. V projektu je bilo vključenih mislim, da vsega skupaj 19 različnih inštitucij, od raziskovalnih pa do razno raznih manjših podjetij ipd. Ukvarjali smo se z razno raznimi vidiki onesnaženosti zraka v mestih in sicer z računanjem in ugotavljanjem emisij snovi v zraku, z meritvami onesnaženosti, z izdelavo različnih modelov in pa na podlagi modelov scenarijev kaj bi se zgodilo, v primeru določenih ukrepov, s kvaliteto zraka. En zelo pomembnih vidikov pa je bila ocena vpliva na zdravje ljudi, in sicer smo ugotavljali izpostavljenost onesnažilom na nivoju posameznika in sicer z uporabo t.i. novih nizko cenovnih senzorskih tehnologij in to je del projekta kamor ste bili vi vključeni. Načrtovano je bilo takole: vključenih je bilo 7 mest, tudi Ljubljana seveda. Ciljali smo na 100 prostovoljcev, v vsakem od teh mest. Na koncu smo jih dobili nekje med 70-80 na sezono, ker je kampanja trajala dvakrat po teden dni v zimskem in pa poletnem obdobju. Pri čemer ste, če se spomnite, imeli v vašem gospodinjstvu en ta uHoo senzor, potem ste s sabo nosili en ta prenosni senzor za delce v zraku in imeli ste tole komercialno dostopno Garmin uro za fizično aktivnost. Poleg tega smo vas prosili za, no vsi ti podatki so se potem zbirali preko brez brezžične povezave oz. mobilnega omrežja v oblaku, kar nam je potem omogočilo pogled in povezovanje izmerjene koncentracije onesnažil doma in tam kjer ste bili, se pravi, povezovanje tudi z vašo aktivnostjo in kdaj so se stvari dogajale. Na ta način smo potem za vas pripravili poročilo z izpostavljenostjo onesnažilom vas kot posameznikov, nam pa bo potem prišlo prav za različne dodatne statistične obdelave in pa podporo pri izdelavi različnih modelov. Če se v tej animaciji malo poigramo s številkami, da	remind you of what we've been doing and here are answers to the questions you've just been solving. [Slide 1]. So, you were involved in the so-called ICARUS project, which is one of the larger Horizon 2020 projects that is ending in April this year [Slide 2]. It was happening in nine cities shown on the map, Ljubljana was also one of the pilot cities. I think, a total of 19 different institutions, from research to a variety of smaller companies, etc. have been involved in this project. [Slide 3]. We dealt with various aspects of urban air pollution, namely the calculation and determination of emissions of substances into the air, the measurement of pollution, the creation of different models and what would happen, in the case of certain measures, with air quality, on the basis of scenario models. One very important aspect was the assessment of the impact on human health, which was to determine the exposure of pollutants at the individual level using the so-called new low-cost sensor technologies and this is part of the project where you have been involved. [Slide 4]. It was planned like this: 7 cities have been included, including Ljubljana of course. We targeted 100 volunteers in each of these cities. In the end, we got them somewhere between 70-80 per season, because the campaign lasted twice a week in the winter and summer. And if you remember, you had one of these uHoo sensors in your household, you were also carrying one of these portable airborne particulate sensors and you had this commercially available Garmin physical activity watch. All this information was then collected via a wireless connection or mobile network in the cloud, which then allowed us to view and connect the measured concentration of pollutants at home and where you were, that is, connecting to your activity and when things were happening. In this way, we prepared a report for you, with exposure to pollutants of you as individuals, and then we will come in handy for various additional statistical processing and support in the production of

dobite občutek s čim smo se mi tu ubadali. Se pravi imeli smo ta prenosni senzor za delce, ki meri tri velikostne razrede od 1, 2.5 in 10 mikronov potem pa še temperaturo, vlago, nadmorsko višino in hitrost. Relevantni podatki za nas od Graminove pametne ure so bili koraki utrip in pa aktivnost. uHoo je zbiral 9 parametrov, ki se zdaj tam prikazujejo, da jih ne naštevam in če vem, da smo meli v povprečju 75 udeležencev iz 50 gospodinjstev, krat 2 sezoni, krat 14 dni in tako naprej, pridemo do 25 milijonov števil, ki smo jih mi na ta način zbrali. In seveda ne rabim poudarjati, da je bil izziv kako jih osmislit in to je eden izmed razlogov zakaj smo danes tu. Poleg tega se spomnite, smo vas prosili, da izpolnite ta dnevnik aktivnosti. To je bilo nekaj kar so ljudje najmanj radi počeli, ker se je bilo treba za nazaj spomniti kaj smo počeli za vsako uro posebej, se pravi to je bila časovna resolucija. Na začetku pa ste odgovorili na 2 tipa vprašalnika. Eden je bil pripravljen za gospodinjstva , drugi pa za posameznike, kjer so potem sledila različna sociodemografska, zdravstvena vprašanja, kakšen tip stanovanja imate in kakšne so vaše življenjske navade. Vse to s ciljem, da se da izbrane podatke postaviti v neki kontekst in si jih osmisliti. Zdaj kar se ciljev tiče, cilji so bili različni. Ti, ki so bili relevantni za vas, kot je to že bilo omenjeno, je vpogled v vašo individualno izpostavljenost glede na način in tip življenja. Nas pa je zanimal tudi čisto tak splošen vidik kako so te nove senzorske tehnologije uporabne in primerne za nekaj takšnega se iti. Potem smo želeli, kar sledi v prihodnje, ko bodo ti podatki v vseh teh mestih ustrezno harmonizirani zbrani, bomo naredili tudi primerjavo med mesti in pa v prihodnje sledi nekaj česar se bo verjetno R3 v doktoratu lotil – podpora teh podatkov, uporaba podatkov za validacijo modelnih orodij kot je agnet-based modeling, v katerem primeru simuliramo gibanje nekega posameznika in poskušamo teoretično izračunati njegove izpostavljenosti s konkretnimi podatki pa validirati, če so modeli smiselni ali ne.	different models. [Slide 5] If we play around with numbers in this animation, you get a sense of what we were dealing with here. So, we had this portable particle sensor, which measures three sizes ranging from 1, 2.5 and 10 microns and then temperature, humidity, altitude and speed. The relevant data for us since Gramin's smartwatch were the steps of the hearth rate and activity. uHoo has collected 9 parameters that are now displayed there that I do not list and if I know that we have an average of 75 participants from 50 households, times 2 seasons, times 14 days and so on, we reach 25 million numbers which we collected them in this way. And of course the challenge was how to design them and that is one of the reasons why we are here today. [Slide 6]. In addition, remember, we asked you to complete this activity diary. This was something that people didn't love to do because they had to remember back in time what they were doing for each hour, that is, it was a time resolution. [Slide 7]. At the beginning, you answered 2 types of questionnaire. One was prepared for households and the other for individuals, where various sociodemographic and health questions followed, what type of housing you have and your lifestyle. All this with the aim of putting the selected data in a context and making sense of it. [Slide 8]. Now, as far as the goals are concerned, the goals were different. Those that were relevant to you, as mentioned earlier, are insights into your individual exposure by lifestyle and type. We were also interested in just such a general aspect of how these new sensor technologies are useful and suitable for something like this. Then we wanted what follows in the future, when these data are properly harmonized in all these cities, we will also make a comparison between the cities, and in the future, something that R3 for a PhD will do - supporting this data, using data for validation modeling tools such as agent-based modeling, in which case we simulate the motion of an individual and try to theoretically calculate his or

	her exposures with concrete data and validate if the models are reasonable or not.

Section 3: Discussion Part 1

**10:15**<br><br>S tem bi jaz tukaj zaključil. To je to na kar so se vprašanja nanašala. Zdaj pa, preden gremo na drugi del, bi vas čisto tako vprašal – ko ste se vi lotili, kaj je vas motiviralo, za to, da ste se udeležili te kampanje? Ima kdo kakšna posebna pričakovanja? Prden gremo pogledat primere rezultatov, ki smo jih pripravili.<br><br>FP1: Mene je zanimalo v kakšnem stanovanju živim. A je zrak v redu ali ni.<br><br>R1: mhm<br><br>FP1: Pa po poti, ko grem v službo. Če spremenim, ko hodim peš ali je boljše, da grem direkt po Dunajski. Verjetno ne. Najbrž ne, ampak zanimalo me je, če se je kaj pokazalo.<br><br>MP2: Ja mene je tudi zanimalo. V bistvu, ker cel dan preživim zunaj in pač kako je to glede onesnaženosti zraka, če se da videti, v katerem področju bolj ali manj.<br><br>R1: Se pravi te je zanimala ta prostorska komponenta ali aktivnost, tvoja?<br><br>MP1: Tudi ja, v povezavi z aktivnostjo.<br><br>FP2: Saj to je zanimivo. Greš ven pa telovadiš pa misliš, da si veliko naredil pa ti potem mogoče pokaže, da ni tako. Recimo.<br><br>R1: Ja, drugač je to en vidik.<br><br>FP1: Prišla sem, ker je R2 zelo lepo prosila pa ker je rekla, da rabite prostovoljce. Pa sem rekla v redu.<br><br>R1: To je ja motiv mnogih, ki so v kakšnih podobnih poklicih in potem razumejo in jih pač	**10:15**<br><br>That is what I would conclude here. This is what the questions were about. Now, before we go to the second part, I would like to ask you this - when you started, what motivated you to participate in this campaign? Does anyone have any specific expectations? Let's go and look at some examples of the results we have prepared.<br><br>FP1: I was interested in what kind of apartment I live. If the air is fine or not.<br><br>R1: mhm<br><br>FP1: And also on the way, when I go to work. If I have to change something. It's better if I walk or it's better to go straight to Dunajska streetProbably not, but I was wondering if something turned out.<br><br>MP2: Yeah, I was also interested. Basically, because I spend all day outside and just how it is in terms of air pollution, if you can see which areas are more and in which less polluted.<br><br>R1: So you're interested in this spatial component or you individual activity?<br><br>MP1: Yes, also in connection with the activity.<br><br>FP2: That's interesting. You go out and workout and you think you did a lot for yourself, but then you realize that this isn't true. For example<br><br>R1: Yes, that is one way to look at it.<br><br>FP1: I came because R2 asked very nicely, and because she said you needed volunteers. So I said okay.<br><br>R1: This is the motive of many who are in a similar profession and who understand this and

zanima.<br><br>FP1: Pa zaradi tega, ker si izvedel kaj se zgodi?	are interested.<br><br>FP1: And because, you found out what happens?

Section 4: Discussion Part 2

**11:57**<br><br>R1: A ima kdo kakšen občutek, kaj bi se zdaj s temi podatki dalo narediti? Glede na parametre, ki smo jih merili, glede na časovno resolucijo, način… Je kdo razmišljal kaj sam o tem ali čakate vsi na neko poročilo? Ali pa v kakšni obliki si vi predstavljate, da bi bilo to poročilo?<br><br>MP1: Ja mogoče v neki obliki priporočil, tudi odločevalcem?? Al pa prebivalstvu z vidika izpostavljenosti zraka.<br><br>FP3: Zemljevidi.<br><br>MP1: Zemljevidi pa so seveda orodje, ne?<br><br>FP3: Mikrolokacije. Pa v poletnih časih...to, ko ste pač enkrat pozimi, enkrat poleti oz. kakšna je razlika, in kaj je največji onesnaževalec…<br><br>FP1: Jaz bi priporočala kak dashboard, ne? Takole za prikaz. A ne, se pravi, da lahko ti pol sam parametre primerjaš? in sam pogledaš kakšni so pozimi, poleti.<br><br>R1: Se pravi celo tako interaktivno orodje.<br><br>FP1: Absolutno ja.<br><br>**13:00**<br><br>R1: To je sicer tudi eden izmed, ravno danes omenjenih produktov tega projekta. Ni čisto direktno vezan na te vaše rezultate, ki so pač vaši osebni in vedno znova podajamo, da jih lahko dobite v čisto surovi obliki in lahko z njimi počnete kar koli želite in znate. Mi smo se pač mogli omejiti na neko agregacijo teh podatkov, ki se nam je zdela smiselna. In zato smo pripravili v bistvu 5 primerov, mogoče bomo potem lažje pol debatirali, če se vam to zdi, ima rep in glavo. Ali	**11:57**<br><br>R1: Does anyone have any sense of what can be done with this information now? Given the parameters we measured, the time resolution, the way… Did anyone think about it alone or are you all waiting for a report? Or in what form do you imagine this report will be?<br><br>MP1: Yes in some form of recommendations to the population in terms of air exposure.<br><br>FP3: Maps.<br><br>MP1: Maps are a tool, of course, right?<br><br>FP3: Microlocations. And comparission between summertime andwinter, and Differences and what is the biggest polluter…<br><br>FP1: I would recommend a dashboard for displaying. But no, that is, you can compare these half parameters yourself? And look at yourself in winter, summer.<br><br>R1: So an intervative evaluation tool.<br><br>FP1: Absolutely.<br><br>**13:00**<br><br>R1: This is also one of the products mentioned in this project today. It is however not directly related to your personal results, and we keep saying that you can get them in raw format and do whatever you want and know with them. We had to limit ourselves to some kind of aggregation of this data that we thought would make sense. And that is why we have prepared basically 5 examples which will help us to debate about them. And if you think they make sense.

bi kdo od vaju še kaj vprašal, smo kaj pozabili?<br><br>R3: Mislim, da smo kar dobro zaobjeli.	Does any of you want tp ask anything else we might have forgot?<br><br>R3: I think that’s pretty much everything.

Section 5: Evaluation

**13:40**<br><br>R1: Ok, potem pa bi vas prosil če obrnete list in sledite navodilom na listih. Se pravi, prikazanih je 5 tipov vizualizacije teh podatkov in zdaj nas zanima, če je ta vizualizacija, način podajana rezultatov razumljiv. V miru preberite, poglejte grafe in poskusite odgovoriti na spodnja vprašanja. Če pa je še kakšno vprašanje za nas pa kar…<br><br>R3: Se bomo potem tudi potem pogovorili.<br><br>R1: Bomo šli skozi skupaj čez to ja.<br><br>[nekdo je mislil, da je že zaključil]<br><br>**15:23**<br><br>R3: Saj so še naprej.<br><br>R1: Kar do konca, 6 strani je teh grafov.<br><br>**17:48**<br><br>FP3: Nimam svojih očal zraven. Eni so premajhni grafi.<br><br>FP1: Absolutno, sploh ne vidim nič. Se bom probala.<br><br>**23:17**<br><br>FP1: A lahko se tudi kaj vpraša?<br><br>R1: Bomo šli pol čez?<br><br>FP1: Aja, ker ne razumem enega od vprašanj.<br><br>R1: Aja, to pa lahko.<br><br>FP1: Kaj prikazujejo sivi prikazani deli grafov. Kaj	**13:40**<br><br>R1: Ok, then I would ask you to turn the sheet and follow the instructions on the sheets [Printed material pages 2-6]. So, there are 5 types of visualization of this data, and now we are interestedif this visualization, the way the results are presented, is understandable. Take your time, read in peace, look at the graphs and try to answer the questions below. But if there are any questions, just ask.<br><br>R3: We'll talk about everything later.<br><br>R1: We'll go through this together, yes.<br><br>[someone thought he had finished])<br><br>**15:23**<br><br>R3: There is more.<br><br>R1: Yes, you have to finish it, there are 6 pages of these graphs.<br><br>**17:48**<br><br>FP3: I don’t have my glasses. Some graphs are too small.<br><br>FP1: Absolutely, I see nothing at all. I will try.<br><br>**23:17**<br><br>FP1: Can I ask something?<br><br>R1: We will go trough this together later<br><br>FP1: Oh, because I don't understand one question.<br><br>R1: Aha ok, you can ask.

so deli grafov? So to deli grafov?<br><br>R1: Aja, zelo smiselno vprašanje.<br><br>FP1: Ali to? Barvno sivo, podatki v grafih.<br><br>R1: Točno to.<br><br>FP1: Aha, ok.<br><br>R1: Si lahko zapomnimo za online verzijo, da ne bo dileme. Za vse tiste, ki niso uspeli priti pa še kakšnemu, za po mailu jih prosit za tole vajo.<br><br>R2: Mhm.<br><br>R3: Ja.<br><br>**27:23**<br><br>FP1: pripomba pri 4. nalogi: kako vem kateri so PM2.5, kateri so pa PM10?<br><br>MP1: To je iz navodil možno razbrat.<br><br>FP2: maš oranžno pa rdečo črto.<br><br>FP1: To sploh ne vem ja.<br><br>R3: Hm, pri zadnji interaciji izdelave grafov zgleda, da je ven padel.<br><br>R1: Ja.<br><br>R3: Hvala.<br><br>FP1: Se pravi, tam ko je rdeča, gre dejansko za PM2.5, tam ko je oranžna pa PM10.<br><br>R1: Pa ni bilo mišljeno, da bi šla tako skelp tako kot je R3 rekel. Ene črte smo so pač.. prvič to tudi vidimo mi<br><br>R1: Preveč smo zbrisali, ne.<br><br>FP1: se pravi druga kolona je PM2.5,tretji graf je ..<br><br>R1: Tako ja, gre 1, 2.5 pa 10.<br><br>FP1: V redu.	FP1: What the gray sections of the graph show. What are graph sections? Are these parts of the graphs? [showing image 3]<br><br>R1: Yeah, a very sensible question.<br><br>FP1: Or that? Color gray, data in graphs.<br><br>R1: Exactly.<br><br>FP1: okay.<br><br>R1: We can remember this for the online version, that there will be no dilemma. For all of those who couldn't come today, we will ask sent them the survey online.<br><br>R2: Mhm.<br><br>R3: Yeah.<br><br>**27:23**<br><br>FP1: Comment on Task 4: How do I know which is PM2.5, and which PM10?<br><br>MP1: This can be seen from the instructions.<br><br>FP2: Orange and red line.<br><br>FP1: I don't even know that.<br><br>R3: Um, It seems like it has been left out during the last iteration oncreating the graphs.<br><br>R1: Yes.<br><br>R3: Thank you.<br><br>FP1: So, when it's red, it's actually PM2.5, and when it's orange, it's PM10.<br><br>R1: But it wasn't meant to be that way, as R3 mentioned. Some lines.. We also noticed this now the first time.<br><br>R1: It looks like we deleted too much.<br><br>FP1: So, the second column is PM2.5, the third graph is ..

R3: Drži ja.<br><br>R1: In to bo ja, tudi pisalo na jasno na teh grafih.<br><br>R3: Bo, bo, je že pa bo spet.<br><br>R2: Je že ja.	R1: So yes, it goes 1, 2.5 and 10.<br><br>FP1: Okay.<br><br>R3: Yes.<br><br>R1: And this will also be written clearly on these graphs.<br><br>R3: It will be, it has been and it will be again.<br><br>R2: Yes, it has been.

Section 6: Discussion Part 3

**32:39**	**32:39**
R1: Ok, jaz mislim, da smo vsi zaključili in nič gremo si pogledat ta čuda. Če imate kakšen komentar, kakšno vprašanje oz. gremo kar skupaj zdaj odgovorit na vprašanja. Pri tem smo kaj vprašali? Kdaj približno je bila izmerjena najvišja temperatura v prikazanem gospodinjstvu?<br><br>FP2: 19. februar.<br><br>R1: V katerem obdobju? Aja, drugač ne zdaj popravljati odgovorov prosim, prav? Ja, 19. februar.<br><br>MP1: Jay mislim da je bilo 18. februar, ampak ja,.<br><br>R1: V glavnem, a je to vsem jasno? Kaj predstavljajo ti grafi? Da gre za 2 sezone, 2x po 1 teden, pa da so vsakič po 3 parametri.<br><br>R2: Gre se za to, da ko boste vi te rezultate dobil, da jih boste znali razbrati. Zaradi tega smo danes tukaj.<br><br>P skupaj: Ja, ja<br><br>FP1: Malo težje je pa, ko moraš gledat tako gor vizualno.<br><br>R2: Mhm, No ja, sej za izboljšave ja.	R1: Ok, I think we're all done and we can look at these together. If you have any comments, questions… let's go together now to answer the questions. What have we asked here? When was the maximum temperature measured in the displayed household approximately?<br><br>FP2: February 19th.<br><br>R1: In what period? Yeah, and please do not alter your answers anymore. Yes, February 19th.<br><br>MP1: To me it looks like February 18th, approximately.<br><br>R1: Basically, is that clear to all? What are these graphs? We have 2 seasons, twice for one week, 3 parameters each.<br><br>R2: The point is that when you get these results you will be able to understand them. That's why we're here today.<br><br>All together: Yes, yes<br><br>FP1: It's a little more difficult when you have to look up every time.<br><br>R2: Mhm, Yes, this is for the improvements.<br><br>R1: So if anyone has any comments, it is very

R1: Tak, da če ima kdo kakšne komentarje, je zelo dobrodošlo, če bo kdo kaj malo pošimfal.<br><br>MP1: Mogoče ta skala, jaz bi to skalo tudi za poletje dal.<br><br>FP2: Ja, jaz sem se tu zmotila, ker sem takoj pogledala, aha itak je poleti višja temperatura in sem takoj …<br><br>R1: Aha, na vseh številke ali kaj?<br><br>FP3: Ja.<br><br>R1: A boš ti vse to pisal? Super<br><br>**34:13**<br><br>R1: Potem drugo vprašanje pa samo ga malo tricky poskušali ga obrnit.<br><br>R3: Kaj pa datumi, ki so na x osi? A to vam je v redu, če so samo odspodaj ali bi bili tudi pod vsakim grafom, da bi bilo bolj pregledno?<br><br>FP1: To sem hotela povedati. Meni bi bilo bolj pregledno, da je pod vsakim grafom. Drugače moraš že skoraj imeti ravnilo al kako bi to sčitali.<br><br>MP1: Odvisno kakšna je natančnost. Mislim.. Za približno je to v redu, za kaj več pa moraš že malo bolj napeti oči.<br><br>FP1: Te črte se malo slabo vidi.nimam očal<br><br>R1: To, da so razponi priporočenih vrednosti označeni z barvno ozadaj, to je tudi kontrastno.<br><br>R2: Barve so v redu ali bi bilo kaj drugače?<br><br>FP1: Ja, sicer to, ko ste napisali rumena poleti, to je za mene bolj oranžna.<br><br>MP1: Drugače vidim, da imate datume, da poleti so prav za vsak dan, za pozimi pa na dva dni. Jaz bi to poenotil.<br><br>FP1: Ja, fajn je, da je skala enotna.<br><br>R1: Kul, še kakšen nasvet?	welcome.<br><br>MP1: Maybe this scale, I would give this scale for the summer as well.<br><br>FP2: Yeah, I made a mistake here because I looked at it right away, but in the summer there is a higher temperature and I'm right…<br><br>R1: Aha, so in practice, in all numbers, right?<br><br>FP3: Yes.<br><br>R1: Will you write all these down, great! [talks to another researcher]<br><br>**34:13**<br><br>R1: Then the second question – we wanted this question to be a little tricky.<br><br>R3: What about dates that are on the x axis? Is this okay for you if they are just below or should they also be below each individual graph to make it clearer?<br><br>FP1: That's what I wanted to say. It would be clearer to me if it was below each graph. Otherwise you must almost have a ruler to read it.<br><br>MP1: Depending on the accuracy. It is about allright, but for something more you have to strain your eyes a little more.<br><br>FP1: Some charts are difficult to see. I don't have my glasses.<br><br>R1: The ranges of the recommended values are indicated by a colored background, which is also contrasting.<br><br>R2: Are the colors okay or sould they be different?<br><br>FP1: Yeah, eventhough when you wrote yellow for the summer, it looks more orange to me.<br><br>MP1: Otherwise I see you have dates that in summer they are for every day and in winter for

MP2: Ne vem, tam mrežne črte se mi zdi, da bi mogoče lahko bile malo bolj temnejše barve. Da bi bili bolj jasen.<br><br>**36:18**<br><br>R1: Nič, gremo na naslednjega ali ima še kdo kaj za pripomnit?<br><br>R3: So tudi enote dovolj jasne?<br><br>R1: Govoriš o prvem še?<br><br>Vsi skupaj: Ja, ja.<br><br>R2: Tudi, če so samo na desni strani napisane tukaj za poleti, moraš desno levo kombinirat ali bi bilo boljše, da bi bilo tudi na levi napisano?<br><br>FP1: Je pa lahko skala na obeh in opis na obeh.<br><br>R2: Ali pa da se nežne črte bolj poudarijo ne? Da se vidi kje je os ne?<br><br>MP2: Ja, da se vidi pač<br><br>R2: kje je os skal.<br><br>**37:06**<br><br>R1: Prav, gremo na drugega [Page 3]. S tem smo se res matrali, moramo priznat. Kako spraviti vso to količino podatkov na par diagramov. Nekateri v konzorciju so se nagibali k tipu poročila, kot je laboratorijski izvid z nekaj številkami, ampak to je nemogoče narediti. Tak da mi smo se poskušali omejiti v številu strani, ki jih boste dobili, da ne bo preveč branja in vsega in še vseeno je kar nekaj strani tega.<br><br>R1: Ok, začnemo: vprašanje je bilo – kdaj pribl. Je bila zabeležena najvišja koncentracija CO2 v poletni sezoni?<br><br>FP1: Jaz imam 23.5., v četrtek.<br><br>FP2: Zjutraj.<br><br>MP1: Ob 7.00 ali 8.00.	two days. I would unify that.<br><br>FP1: Yes, the fine thing is that the scale is the same.<br><br>R1: Cool, any other advice?<br><br>MP2: Maybe, there, the grid lines could be a little darker to be clearerer.<br><br>**36:18**<br><br>R1: Ok, so, let's go to the next one, or is there anything else you might want to comment on?<br><br>R3: Are the units clear enough?<br><br>R1: Are you still talking about the first one?<br><br>All together: Yes, yes.<br><br>R2: How about thatnow they are only written on the right here for the summer, and you have to combine the right and left or would it be better if it was written on the left as well?<br><br>FP1: Scale can be on both charts as well as the description.<br><br>R2: Or to emphasize the fine lines? To see where is the axis?<br><br>MP2: Yeah. So they would be more visible.<br><br>R2: To see the axis<br><br>**37:06**<br><br>R1: Okay, let's go to another one [Printed material, page 3]. We really struggled with this, we have to admit. How to get all this data into a set of diagrams. Some in the consortium tended to prefer a type of report, similar to lab reports with numbers, but this is impossible to do. So we tried to limit ourselves to the number of pages that you will get, so that there is not too much reading and everything and yet there is a still a lot of pages.<br><br>R1: Ok, let's starR2: the question was - When

FP1: Aja to pa nisem nič več videla.

MP1: Ob 7.00, mislim ne vem.

FP2: Jaz sem napisala samo zjutraj.

FP1: Ampak bodo dejansko potem tako majhne številke?

R2: Ja je kar majhno, tudi jaz ki dobro vidim, imam težave.

R3: V končnem poročilo bo malo večje, samo tule je pač v tabeli, ker so še vprašanja zraven pa smo hoteli, da je vse na eni strani. Tako da bo bolj večja.

R1: Pa če ne drugo, tam bo v pdf pa se bo dalo povečati.

R3: Tudi to je res.

38:56

R1: Se pravi OK, če ste to razumeli potem ste avtomatsko dešifrirali, da govorimo o zimi in poletju, da ima vsaka svojo barvo, našli ste kje je CO_2 in potem ste tudi pogruntali, da je za vsak dan za vsako uro posebej različno obarvano.

Vsi skupaj: Mhm.

R1: In potem, če ste ugotovili to, niste imeli težav z drugim vprašanjem, ki se glasi: ali so izmerjene vrednosti NO_2 v zimskem obdobju v popoldanskem času višje ali nižje od dopoldanskih?

Vsi skupaj: Višje, višje.

39:30

R1: To je en tak jasen trend in nekdo, ki bi to dobil, je to je ta informacija, ki jo želimo podati. Tu nismo operirali tudi zaradi negotovosti rezultatov povezanih z temi nizkocenovnimi senzorji, imamo predstavnika za ARSO, ne bom ga identificiral, vedno dajem kot primer. Tale reč, ki jo lahko sami kupite mislim, da stane med 300 in 400€ in seveda ne moremo pričakovati enako

approximately was the highest CO_2 concentration recorded during the summer season?

FP1: I have on 23.5, on Thursday.

FP2: In the morning.

MP1: At 7am or 8am.

FP1: I didn’t see that.

MP1: At 7.00, hard to say.

FP2: I only wrote in the morning.

FP1: But will the numbers actually be so small?

R2: Yeah, it's small. Even if I see well, I also have problems here.

R3: It will be a little bigger in the final report. Here we inserted the images in a table in order to fit also the questions on the same page.

R1: Report will also be in .pdf form, so you will be able to zoom it.

R3: That's also true.

38:56

R1: So OK, if you understood that, then you automatically deciphered that we are talking about winter and summer, that each one has its own color, you also found where the CO_2 is and then you also figured out that for each day and hour there was a different color.

All together: Mhm.

R1: And then, if you found that out, you had no problems with the second question, which is: are the measured NO_2 values in winter season in afternoon higher or lower as in the morning?

All together: Higher, higher.

39:30

R1: This is such a clear trend and this is the

natančnih podatkov, kot nam jih daje ARSO, ker tista naprava stane 100 tisoč € pa še vsaj en človek je zaposlen, ki to kalibrira in se matra vsak dan s tistim. Je treba imeti pač realna pričakovanja glede te natančnosti. Zato smo se odločili za relativni prikaz v smislu več je manj in to je mislim da informacija, ki jo človek želi da ugotoviti. Aha nekaj se mi dogaja v stanovanju, ker vsak dan popoldne so povišane koncentracije NO2, v tem primeru.<br><br>**40:29**<br><br>R3: Hkrati s tem, ko boste dobili te svoje dnevnike, ki ste beležili aktivnost, ne, boste lahko direktno primerjali, aha, v četrtek popoldne se je to pa to dogajalo in lahko malo iz tega sklepate potem sami. Se pravi, ne bomo zdaj mi dajali neko interpretacijo, boste potem sami malo pogledali pa razmislili kaj se dogaja.<br><br>R1: To je bil tudi eden izmed glavnih zaključkov, če bo interes pa če kaj časa ostane, imam tudi z ene druge prezentacije par slajdov, z nekimi takimi reprezentativnimi tipičnimi rezultati, kaj se zdaj vidi. Ugotavljamo, da brez dejanskega poznavanja kaj je človek počel, čeprav imamo tu napisano v temu dnevniku aktivnosti, je strašno težko interpretirat. Vsak lahko samo zase ve kaj ti rezultati pomenijo. Tako da, mi smo se razen teh priporočil na koncu poskušali izogniti nekim zaključkom, ker jih lahko vsak zase potegne samo vsak posameznik, za začetek pa mora razumeti kaj gleda. No, in če pa razumemo to, je odgovor na tretje vprašanje potem tudi jasen. Ali lahko delamo direktno primerjave med zimskimi in poletnimi grafi? To je to, kar se tu sprašuje.<br><br>**41:38**<br><br>FP1: Jaz sem napisala, da ne, ker so relativni.<br><br>FP2: Ker so relativni, ja.<br><br>R1: Tako. Tu ja, te grafi ne povejo nič o dejanskih številkah, ampak samo o tem ali je bilo zjutraj več in manj in tekom dneva, kako se je, so se	information we want to give to you. We also didn't operate here because of the uncertainty of the results, associated with low cost sensors. We have a representative from SEA here, but I will not identify him. I always give the following example. You can buy this kind of thing for yourself, I think it costs between 300 € and 400 € and of course we can't expect the same exact information as SEA gives us, because that device costs 100,000 € and there is at least one person who is employed to calibrate and deal with it in daily basis. It is necessary to have realistic expectations about the accuracy. So we decided on a relative display in the sense ofmore is less, and that this is the information that a person wants to find ouR2: aha, something is happening in the apartment. Because, in this case, the concentrations of NO2 are high every afternoon.<br><br>**40:29**<br><br>R3: Ones you will get your TADs, you will be able to compare directly: aha, on Thursday afternoon, this and that was happening and you can then make your own conclusionst. So, instead of us providing you the interpretation, you can take a look by yourself and think about what is going on.<br><br>R1: That was also one of the main conclusions, And if you have interest andif there is any time left, I will also present from one other presentation few slides, with some representative typical results, of what we also see now. So far we concluded, that without actually knowing, what the person was doing, even though we have it written in this time activity diary, it's terribly difficult to interpret. Everyone can know for themselves what these results mean. So, in addition to these recommendations in the end, we tried to avoid any conclusions, because everyone can draw them for themselves, but first they need to understand what they are looking at. Well, and if we understand that, then the answer to the third question is also clear. Can we make direct

vrednosti spreminjale, ali gor ali dol. Zato sta tudi izbrani dve različni barvi za vsako sezono, da nebi bilo kakšne zmede okrog tega. In tudi nikjer niso napisane številke. Samo z nizko in visoko operiramo.<br><br>FP3: Jaz sem pa prej občutek dobila, da je pač poleti. Glih zato, mogoče ker je ta barva rdeča.<br><br>R2: Aja.<br><br>R1: Aja, da je taka. Mogoče bi mi to v tekstu dodali, da gre za relativne in kar pomeni, da tretirajte kot opozorilo.<br><br>R3: Malo bolj poudarimo to.<br><br>R2: Da ja, ni treba drame delat iz tega.<br><br>FP1: Zdaj, če bi videli kako je visoko poleti in kako pozimi, bi že lahko primerjali, ne?<br><br>**42:39**<br><br>R1: Ja pa tudi bi lahko šli v detajle okrog tega. To je v svojem magisteriju R3 raziskoval. Natančnost teh podatkov. To bi bilo v možno tudi v primeru, če bi enako enoto, isto enoto uporabljali in pozimi in poleti. Kar pa v večini primerov ni bilo in se ne da delati direktne primerjave, ker je ta offset tako velik med enotami. Če poenostavim: če mi damo 20 takšnih instrumentov na kup, bodo vsi kazali iste trende gor in dol, absolutne številke pa bodo bistveno drugačne med eno in drugo enoto. Se pravi, ne moremo zaupati, ker bi jih mogli v vsakem gospodinjstvu posebej z drago opremo predhodno skalibrirati in to je pač out of scope te raziskave. Nemogoče no. In en izmed raziskovalnih izzivov kako to rešiti v bodoče, ker teh firm, ki prodajajo razno razne senzoriko oz. vsak srednješolec, ki zna malo programirat, si lahko na kitajskem za nekaj 10 € naroči arduino in senzorje in sestavi skupaj in tista škatlica bo začela bruhat podatke. Kako so pa smiselni, je pa drugo vprašanje. A, ja, še kaj v zvezi s tem grafom? Je bilo dovolj teksta zraven, da se je dalo razbrati? Očitno ja.	comparisons between winter and summer graphs? This is what is being asked here.<br><br>**41:38**<br><br>FP1: I wrote no because they are relative.<br><br>FP2: Because they are relative, yes.<br><br>R1: That's right. These graphs tell nothing about the actual numbers, but just about if there were more and less in the morning, how it was, if the values varied, if it goes up or down. That's why two different colors were selectedfor each season so that there is no confusion about it. Andnumbers are nowhere written. We only operated with “low” and “high”.<br><br>FP3: I just got the feeling it was summer. Maybe it's because this color is red.<br><br>R2: Yeah.<br><br>R1: Oh, so is like this. Maybe we have to add this to the text and emphasize in a disclaimer that it is relative.<br><br>R3: Let's emphasize that a little more yes.<br><br>R2: Yes, you don't have to make drama out of it.<br><br>FP1: Now, if you could see how high it is in summer and how it is in winter, you could already compare, right?<br><br>**42:39**<br><br>R1: Yeah, well we could go into the details about that. In his Master's thesis, Rock was studying the accuracy of this data. This would also be possible if the same unit were used both in winter and summer. But in most cases this was not the case and it is not possible to make direct comparisons, because this offset is so large between units. To simplify: If we put 20 such instruments together, they will all show the same trends of ups and downs, but the absolute values will be significantly different between units. So, we cannot trust this, because they

44:12

FP1: Jaz bi samo rekla, če se to kaj v oklepaju spremeni – CO2, NO2, … tako kot se sledilo dol, je sicer pikolovsko, ampak…

R1: Ne, ne to je vse prav.

R2: Sosledje ??

R1: To bi bilo vse narejeno, ampak je naša pikolovska sodelavka na materinskem dopustu.

MP1: Saj to pojasnilo glede poimenovanja dni [v Angleščini] je po mojem odveč. Čisto tako mimogrede. Ker na prvi pogled, se mi zdi da so navodila zelo dolga. Ampak ni tako hudo, kokr mogoče zgleda

44:51

R1: Sicer je v dejanskem poročilu, en del. Ni vse to kot fiber caption, ampak je en del tega teksta v samem tekstu v razlagi. Mi smo samo ekstrahirali ven.

FP1: Ampak to so poročila, ki so potem namenjena slovenskim udeležencem ne? Zakaj potem ne bi poslovenili tudi legende?

R3: Malo je težav v programskem jeziku, ker je delano v aru (R) vse skupaj in je malo bolj kompleksno ker je težko iz šumnike operirat in take zadeve in... Na koncu smo potem tehtali, ali si vzeti par ur pa tako zadevo popraviti, ali dodaten stavek napisat. Nekje sicer smo probali pripraviti. Recimo nizko – visoko, tam je bilo prej low pa high. Tako da nekje smo pa probali popraviti. Bom pa še enkrat tudi to pogledal, če se da.

45:47

R1: Ja, ker ta poročila so v resnici avtomatsko generirana. Mi ne moremo iti v vsakega posameznika pogledat, ker to bi pol tudi zgledalo, da bi vas klicaili in spraševali kje ste bili pa kaj ste delali pa zakaj …

would need to be pre-calibrated with expensive equipment in each household, and this is just out of the scope of this research. Impossible. And one of the research challenges is how to solve it in the future, because of these companies that sell various sensor devices and any high-schooler who can program a little can for few tens of euros order from China an arduino and assemble couple of sensors togetherand that box will start producingdata. How much they make sense, is another question. Oh, yeah, anything else about this graph? Was there enough accompanying text for it to be clear? Apparently yes.

44:12

FP1: I would just say, to change whats inthe brackets - CO2, NO2,… to be in subscrip, which is just a detail, but…

R1: No, no that's all right.

R2: Subscript you mean?

R1: That would be all done, but our co-worker prone to details is on amaternity leave.

MP1: I think this clarification on naming the days [in English] is unnecessary. Just a comment. Because at first glance, the explanations of the charts seem very long, but it is not as bad as it seems.

44:51

R1: Otherwise, it's in the actual report, one part. Not all of this is made like fiber caption, but one part of this text is in the text itself in the explanation. We just extracted it.

FP1: But these are reports that are then intended for Slovene participants? Then why not do a legend in Slovenian language?

R3: It's a little bit of a problem in the programming language, because it is done in R and it getsa little more complex and it's very difficult to operate with sibilants (is this translate

OK, če ni v zvezi s tem več nič, gremo na graf 3, slika 3.

46:29

FP1: Mene je tukaj malo zmotilo. NA piše ni podatka ne, podatek manjka, mamo pa veliko teh pikic. To me je čisto zmedlo. Nisem vedela kaj naj zdaj. Recimo, ko smo imeli, koliko je najvišje prikazana koncentracija PM2.5: če jaz pogledam PM2.5 pa mi kaže tam 120 pa je siva.

MP1: To samo pomeni, da nimamo podatkov o aktivnosti.

FP1: To bi bilo fajn, da piše, da podatek manjka.

FP2: Ja, ni podatka o aktivnosti.

R1: Super.

FP1: To me je čisto zmedlo, pol nisem vedela kaj naj gledam.

R3: Super, super.

47:31

R1: Zdaj verjamem, da ste ugotovili, da če ne drugo, da za zimo podatki manjkajo, ne?

Vsi skupaj: ja

R1: In da potem, prvo vprašanje se je samo na poletje nanašalo. Kdaj je bila najvišja koncentracija PM2.5, to ste našli, da gre za tri velikostne razrede delcev in spodaj je še srčni utrip.

R2: Je to jasno, da je tako?

R1: In da je PM2.5 tu v tej drugi koloni in da je nekje tule 120 ta vrednost. Ja seveda, če pa se ne razume pa je strašno zavajajoče.

R2: Saj mogoče tudi pač sama barva je problem ja, da je siva.

FP2: Samo jaz mislim, da nikoli ni – podatek manjka. A ni takrat bilo meritve, ali vemo…?

for Š Č Ž letters?). In the end, we then weighed whether to take a couple of hours to correct this, or just write an additional sentence. We tried to prepare some things. Let's say “nizko” and “visoko” used to be “low” and “high” at the beginning. But I'll take a look at this again, and see what we can do about it.

45:47

R1: Yes, because these reports are automatically generated. We can't go into every single person because it would look like this: we would need to call you and ask where you were, what you were doing, and why…

OK, if there is nothing mote to addLet’s go to graph 3, figure 3 [printed material, page 4].

46:29

FP1: I was a little confused here. NA means no data, or missing data, but there is a lot of these dots. It just confused me. I didn't know what to think. Let's say when we had a question - how much was the maximum PM2.5 concentration: if I look at PM2.5, it shows me 120 but it's grey.

MP1: It just means we have no data of the type of activity.

FP1: It would be nice to say the information is missing.

FP2: Yes, there is no data of activity.

R1: Great.

FP1: It just confused me. I didn't know what am I looking at.

R3: Great, great.

47:31

R1: Now I believe, you have figured out that data for the winter is missing, right?

FP1: Ja nekje je mogla biti.<br><br>MP1: Samo meritve so bile, ker imaš, ne?<br><br>**48:30**<br><br>R3: Ja super, sicer najnižji srčni utrip se mogoče malo slabše vidi ampak ste verjetno našli to pikico, ki je malo nižje kot vse ostale.<br><br>FP1: 17. maj ponoči.<br><br>R3: Tam nekje ja.<br><br>FP2: Ja, ja.<br><br>**48:49**<br><br>R1: In potem tretje: koliko je najvišja PM10 koncentracija izmerjena 19. maja med športno aktivnostjo?<br><br>MP1: 75.<br><br>FP1: Ni bilo športne aktivnosti, ker nisem videla barve.<br><br>FP2: 75.<br><br>R1: Govorimo tukaj ne? Smo na PM10, športna aktivnost.<br><br>FP1:športna je oranžna<br><br>FP3: športna je roza.<br><br>R1: Se pravi smo tukaj. 19.5. Se je tu začel in je potem tukaj. To smo meli v mislih, tole pikico.<br><br>R3: A bi bilo to fino, če so te pikice gor v legendi večje?<br><br>FP1: Ja.<br><br>MP1: Ja.<br><br>FP2: Ja.<br><br>R2: Ker v bistvu sta dva roza slabo vidna, ker «clean» pa «sport» se mi zdi, da je skoraj enaka barva.	Participants all together: yes<br><br>R1: And then that the first question was only about summer. When was the highest concentration of PM2.5? And you found out there are three size classes of particles and on the bottom there is alsoheart rate.<br><br>R2: Is that clear?<br><br>R1: And that PM2.5 is here in this second column and that somewhere around 120 the value we were looking for. Yes, now I see, if someone doesn't understand this, it's terribly misleading.<br><br>R2: Maybe the color itself is a problem. Because it's gray.<br><br>FP2: But the way I understand this is that there was never data missing. But there was no measurement then, or something?<br><br>FP1: Yeah, they should besomewhere.<br><br>MP1: The measurements were, because you have here…, right?<br><br>**48:30**<br><br>R3: Great! Maybe the lowest heart rate is hard to see, but you probably found this dot, which is a little lower than all the other ones.<br><br>FP1: May 17 at night.<br><br>R3: Yeah, there somewhere.<br><br>FP2: Yeah, yeah.<br><br>**48:49**<br><br>R1: And then thirR1: how much is the highest PM10 concentration measured on May 19 during sports activity?<br><br>MP1: 75.<br><br>FP1: I wrote that there was no sport activity because I didn't see the color.

**49:50**<br><br>MP1: Tudi ta ura med clean pa sports je zelo podobna, vsaj v legendi no, zdaj tam na grafu ..samo graf, saj sploh ne vem kje je to čiščenje.<br><br>R3: Lahko, da ga ni, ker ni bilo čiščenja, ali je pa samo ena pika, ki se skrije.<br><br>R2: Da ni prevladujoče ja, niso nič čistili.<br><br>R1: Ja pa res.<br><br>Vsi: haha<br><br>R1: Razen, če kdo cleaning jemlje kot športno aktivnost.<br><br>FP2: Haha, da lažje čistiš.<br><br>R3: Pa je res, zgleda da je vmes med «cooking»om, čisto zraven je mejčkena rdeča «cleaning».<br><br>**50:41**<br><br>R1: To je potem treba menjat za bolj poudarjeno barvo. Ja na to četrto pa smo že odgovorili. Kaj prikazujejo sivi prikazani deli grafov oz. sive pikice.<br><br>MP1: Pač, da nimamo podatkov o vrsti aktivnosti.<br><br>FP1: Jaz sem čist padla, ker nisem nič kapirala Sem napisala koncentracije PM o počivanja in utrip, sem čisto padla tukaj.<br><br>R2: Saj to pomeni, da smo mi narobe zastavili in je treba izboljšati.<br><br>FP1: Kaj prikazujejo sivi deli grafov. To so sive pikice, a ne?<br><br>R3: Mhm<br><br>FP3: Sivo prikazane vrednosti…<br><br>MP1: Ja mogoče, formulacija bi lahko bila boljša.<br><br>R1: Ja tudi smo lahko jasni bolj v tem online	FP2: 75.<br><br>R1: We're talking about this? We are on PM10, a sports activity.<br><br>FP1: Sport is marked orange<br><br>FP3: Sport is marked pink<br><br>R1: So we're right here. 19.5. Started here and is here. That's what we had in mind, this dot.<br><br>R3: Would it be nice if those dots are bigger in the legend?<br><br>FP1: Yes.<br><br>MP1: Yeah.<br><br>FP2: Yes.<br><br>R2: Because basically two pink dots are poorly visible, but 'clean' and 'sport' seem to me to be almost the same color.<br><br>**49:50**<br><br>MP1: Also occurance of 'clean' and 'sport' in the timeline is very similar, at least in the legend. Well, now there on the graph... only the graph… actually I don't know where the 'clean' is.<br><br>R3: It's possible that is not there, because the person wasn't cleaning, or there may be only one dot, which have been hidden.<br><br>R2: Is not dominant. They didn't clean anything.<br><br>R1: Yeah, in deed.<br><br>All: haha<br><br>R1: Unless someone takes cleaning as a sports activity.<br><br>FP2: Haha, to make it easier to clean.<br><br>R3: Well, it looks like it's between the "cooking", right next to it there is a small red dot "cleaning".

vprašalniku.<br><br>R3: Če bi mogoče kar tam, kjer je ta siva pikica napisali nekak podatek manjka. Or something like that.<br><br>FP3: Ali pa neopredeljena aktivnost. Ker imate podatek, samo ne vem točno...<br><br>R1: Pa še NA lahko ostane.<br><br>R3: Neznana aktivnost haha.<br><br>**51:54**<br><br>R1: Ja, vse jasno ker jaz poznam poklic od xy pa ne smem povedat, ker je vse anonimizirano.<br><br>FP2: Zakaj se ne sme povedat?<br><br>R1: Malo se hecamo. Mi smo imeli toliko enih težav z to anonimizacijo, da si vi sploh ne morete predstavljat. Mi nismo operirali z vašimi imeni in to smo se zgubljali v teh…<br><br>R2: Dejansko sploh ne vem zdaj točno. V bistvu je bil pod gospodinjstvo ID pa še posameznik pod ID pa še 2 sezoni sta bile, in...<br><br>R1: In vse se je zbiralo na podlagi generiranih mailov, ki so bili generirani na podlagi vaših ID in to je šlo gor v oblak itd. … vse pod ključem.<br><br>R2: Ja, vse imamo v vartnosti omari.<br><br>**52:47**<br><br>R1: Tole povezano z zadnjim vprašanjem ne, kaj prikazuje graf za srčni utrip v zimskem obdobju. Vam je to bilo jasno? Verjetno ne.<br><br>MP1: Bolj ne.<br><br>FP2: Da ni podatkov.<br><br>FP1: Ni podatkov ja.<br><br>R3: Da ni podatkov o srčnem utripu, imamo pa	**50:41**<br><br>R1: That must then be changed to a more visible colour. Ok, we have already answered this fourth question. What does the gray sections of the graphs or gray dotsmean?<br><br>MP1: That we don't have data about the type of activity.<br><br>FP1: I didn't understand it, so I wrote the PM concentrations during resting and hearth rate. I really didn’t get it.<br><br>R2: That means that we were not clear enough and need to improve this.<br><br>FP1: What the gray sections of the graph show. These are the grey dots, aren't they?<br><br>R3: Mhm<br><br>FP3: Gray values displayed ...<br><br>MP1: Yeah, maybe the [sentence] formulation could have been better.<br><br>R1: Yes, we can also be clearer in the online questionnaire.<br><br>R3: We could just write, where that gray dot is, that it means “information is missing”.<br><br>FP3: Or undetermined activity because there is data...<br><br>R1: It means, we can mark it as “NA”.<br><br>R3: Neznana Aktivnost (NA) haha.<br><br>**51:54**<br><br>R1: Yeah, everything is clear because I know the profession of this participantsbut am not allowed to share with you because it's all anonymized.<br><br>FP2: Why can't it be said?<br><br>R1: We're kidding a little bit. We've had many issues with this anonymization that is hard to imagine. We didn't operate on your names and it

podatke o aktivnosti.<br><br>FP1: o aktivnosti pa so<br><br>MP1: Potem to nič ne pove, pol je premalo, da lahko kar koli..<br><br>R3: Načeloma edina stvar, ki bi jo lahko tu ven sklepali je to, da vidite katera barva in sledite aktivnostim, ki so se takrat dogajale. S tem smo tudi hoteli prikazati, da se bodo pojavljali grafi, ki so prazni pa ker ni podatkov pa tako naprej. Zaradi tega, ker senzorji so delali pa niso delali pa tako naprej.<br><br>R1: Sploh v zimski sezoni vas lahko npr. doleti. Imeli smo tehnične težave s temi senzorji in niso merili časa in lokacije npr. in enostavno tovrstnih podatkov ni. So samo agregirani mogoče za celo obdobje kot neko povprečje pa min in max, ne pa do minute natančno.<br><br>FP1: To smo domakr najbolj tečno za nositi.<br><br>R3: Saj za poletje je boljše.<br><br>FP3: Ampak ja, ta podatek manjka, za prejšnje pa kar prikazujejo sivi deli pa neopredeljena aktivnost. Dve različni stvari sta. Za to mislim. Kaj prikazuje graf za srčni utrip v zimskem obdobju. Prikazuje srčni utrip v zimskem obdobju hehe.<br><br>R1: Ja, mogel bi.<br><br>FP3: Ne vem kaj prikazuje. Kaj lahko razberete iz grafa za srčni utrip, recimo.<br><br>MP1: to je pogled lektorice prevajalke ne?<br><br>Vsi: Hahaha<br><br>R3: To je dobro ja, da lahko za spletno anketo drugače napišemo.<br><br>FP3: Jaz sem par vejic dodala notri, haha.	is easy to loose track on…<br><br>R2: I don't remember exactly now, but there was different ID for household and another one for each individual and then there were 2 seasons…<br><br>R1: And everything was collected based on the generated mails that were generated based on your IDs and that was uploaded into the cloud etc. … Everything is behind secure doors.<br><br>R2: yeah, we have a security closet for this.<br><br>**52:47**<br><br>R1: This is related to the last question: what does the heart rate chart show in the winter? Did you understand that? Probably not.<br><br>MP1: Not really.<br><br>FP2: That there is no data.<br><br>FP1: No data available yes.<br><br>R3: There is no heart rate data, but we have activity data.<br><br>FP1: Activity is there.<br><br>MP1: Then that doesn't say anything, then it is too little to tell anything ..<br><br>R3: In principle, the only thing you can deduce here is the color and follow the activities that were going on at the time. With this we also wanted to show you that there will be graphs that are empty because there is no data… Because the sensors sometimes worked and sometimes didn't etc.<br><br>R1: Especially for the winter season, you may come across this. For examplewe had technical issues with these sensors that they did not measure time and location and so there is simply no information. They can only be aggregated for the whole period in average and also MIN and MAX value, but not up to a minute resolution.<br><br>FP1: This was the device that was the most

R3: Super<br><br>**55:10**<br><br>R1: Ok, gremo dalje. Graf 5 [Printed material, page 5].<br><br>FP2: 4.<br><br>R1: 4.<br><br>R3: Tukaj ni napisanih tistih …<br><br>R1: Ja, A je kdo imel kaškme pripombe pomisleke… Preden gremo na vprašanja.<br><br>**55:38**<br><br>R1: Ok, prav. Koliko dni v tednu in v kateri sezoni so bile koncentracije PM2.5 presežene?<br><br>FP2: 6.<br><br>MP1: 6x pozimi.<br><br>FP1: v zimskem času vse razen 7.<br><br>R3: Ok. A je kdo drugače odgovoril?<br><br>**56:02**<br>R1: In podobno vprašanje za PM10: koliko dni in v kateri sezoni?<br><br>MP2: 1x pozimi<br><br>R1: Ok, to je bil naš namen, da se res vidi razliko, da gre za dve različni mejni vrednosti, ena je označena tako, druga pa drugače in če to razberete je to super.<br><br>MP1: Ampak brez navodil pa to nebi šlo, ne.<br><br>FP2: Mogoče bi bilo boljše, če bi tukaj pri grafih bilo označeno PM1, PM2.5.<br><br>MP1: Jaja, mogoče tukaj ob tej.<br><br>R1: Ja, tudi za to smo se že prej, smo ugotavljali, da po nesreči manjkajo ja.<br><br>MP1: jaja.	annoyign to carry at home.<br><br>R3: It's better for summer.<br><br>FP3: Bu yeah, okay so this information is missing, and the gray sections show unspecified activity for the previous ones. There are two different things. What does the heart rate chart display in the winter? It shows the heart rate in winter, hehe.<br><br>R1: Yeah, it could.<br><br>FP3: I don't know what the graph is showing. [But it could be] “What you can tell from the heart rate chart”, for example.<br><br>MP1: This is a proofreader’s point of view, right?<br><br>All: Hahaha<br><br>R3: That's a good yes, we can write it differently for an online survey.<br><br>FP3: I added a few commas as well, haha.<br><br>R3: Great<br><br>**55:10**<br><br>R1: Okay, let's move on. Graph 5.<br><br>FP2: 4.<br><br>R1: Graph 4.<br><br>R3: Here it is not written the ...<br><br>R1: Yeah, Does anyone still have any comments or before we go to the questions.<br><br>**55:38**<br><br>R1: Okay, right. How many days per week and in which season were PM2.5 concentrations exceeded?<br><br>FP2: 6.<br><br>MP1: 6 times in winter.

R3: To bo pa definitivno zraven ja.<br><br>FP2: V bistvu jaz nisem razumela, kaj je ta prvi graf na levi.<br><br>FP1: Ja, ker ni bilo oznake za koncentracijo za 1, ja.<br><br>R2: Aha ja.<br><br>FP3: PM1<br><br>FP2: PM1 ok.<br><br>R1: Ja, to smo prej že ugotovili, da je v zadnji varianti je izginilo tisto.<br><br>R3: Ja.<br><br>R1: Prej je bilo vsega preveč, pol smo pa par črt zbrisali in očitno še imena.<br><br>R3: Ker v tem primeru pa WHO za PM1 nima smernic. Tako da, a je to smiselno tudi napisat, zakaj tam ni črte?<br><br>MP1: Ja, ne bi bilo slabo.<br><br>R2: Ja, ker potem na unihdveh so.<br><br>**57:24**<br><br>R1: In tudi, ko boste gledali svoje številke, to so 24 urna povprečja. Vi ne smete te vrednosti gledati pri prejšnjem grafu, ko je za vsako minuto podatek. Samo to celo povprečje. Zato je dodana še ta verzija istih rezultatov.<br><br>R3: Mhm.<br><br>**57:44**<br>R1: Aha in koliko je potem mejna vrednost za PM2.5?<br><br>FP1: 25.<br><br>FP2: 25.<br><br>R3: Drži ja.<br><br>**57:51**	FP1: All in winter except 7.<br><br>R3: Okay. Has anyone answered differently?<br><br>**56:02**<br><br>R1: And a similar question for PM10: how many days and in which season?<br><br>MP2: 1x in winter<br><br>R1: Okay, it was our intention, that is is possible to see a difference, that there are two different thresholds, one is labeled that way and the other different way and if you understand, that’s great.<br><br>MP1: But without the guidance, it wouldn't be possible.<br><br>FP2: Maybe it would be better if PM1, PM2.5 is indicated here in the graphs.<br><br>MP1: Yes, maybe here at this one.<br><br>R1: Yeah, well, that's what we were talking about earlier, that we found out that they were accidentally missing yes.<br><br>MP1: Yeah.<br><br>R3: That will definitely be included.<br><br>FP2: Actually, I didn't understand what this first graph on the left is.<br><br>FP1: Yes, because there was no label for concentration of 1, yes.<br><br>R2: Yeah.<br><br>FP3: PM1<br><br>FP2: PM1 ok.<br><br>R1: Yeah, we found out earlier that it disappeared in the last version.<br><br>R3: Yeah.<br><br>R1: Earlier there was too much of everything so we deleted few lines and obviously also the

R1: Nekaj podatkov o koncentracijah PM manjka, za koliko dni in v kateri sezoni?<br><br>FP1: Kurilna sezona, 5. in 6. dan, se pravi 2 dni.<br><br>FP3: 3 dni.<br><br>FP2: Pač tukaj, pozimi je za 7 dni, poleti pa za 6 dni.<br><br>R1: To smo mi meli debato ja.<br><br>R2: Eni ste bili udeleženi vedno, eni manj, eni pa celo več.<br><br>FP2: Ja, ampak koliko dni je, ne vem.<br><br>FP1: Na grafu sta potem 2.<br><br>R1: To je bil smisel tega vprašanja, ki ga pač potem ob reportu ne bo. Zakaj pride do tega, ne? Je da, očitno, ker je, zopet poudarjam, to je avtomatsko generiranje poročil. Če bi človek bil udeležen samo 4 dni, potem bi bili samo 4 dnevi prikazani očitno je bil 6 dni in za zadnja 2 dneva nimamo podatkov o PM.ju. So pa neki drugi podatki, o aktivnosti, ki jo je sam beležil in podobno. In to je ta informacija, ki bi jo človek naj dobil, zato smo se odločili, da damo 1x 7, 1x pa 6, ker so to realni podatki. Se pravi nekdo, mi pričakujemo, da nekdo ki bo to pogledal, bo videl: aha, pozimi sem 7 dni sodeloval in imam vse podatke, očitno za poletje pa sem sodeloval samo 6 dni in samo za prve 4 dni imam podatek o PM. Nosi neko informacijo v bistvu. Nima pa smisla v tem kontekstu tegale vprašanja. To se pa strinjam.<br><br>MP1: Ja.<br><br>**59:27**<br><br>R1: Še kaj v zvezi s tem? Ali gremo na zadnjega? Kakšno generalno priporočilo?<br><br>**59:51**<br><br>FP1: Mislim, da manjka play v razlagi.	names.<br><br>R3: Because in this case, the WHO has no guidelines for PM1. So, would it make sense to write why there is no line there?<br><br>MP1: Yeah, that wouldn't be bad.<br><br>R2: Yes, because they are at the other two.<br><br>**57:24**<br><br>R1: And you should know when you are looking at your results, those are 24 hour averages. You should not look at this [limit] value in the previous graph when there was data displayed for every minute. Just for the average. Therefore, this version [daily averages] of the same results is added.<br><br>R3: Mhm.<br><br>**57:44**<br><br>R1: Yeah, and then what is the threshold for PM2.5?<br><br>FP1: 25.<br><br>FP2: 25.<br><br>R3: True.<br><br>**57:51**<br><br>R1: Some data for PM concentrations are missing, for how many days and in what season?<br><br>FP1: heating season, 5th and 6th day, so 2 days.<br><br>FP3: 3 days.<br><br>FP2: Here, it's for 7 days in winter and 6 days for summer.<br><br>R1: That's what we had a debate about.<br><br>R2: Some of you have attended the whole time, some less and some more.<br><br>FP2: Yeah, but how many days it is, I don't know.

R3: Mhm, drži ja.<br><br>R1: Kaj manjka?<br><br>R3: Play v razlagi.<br><br>R1: Aha, ok.<br><br>MP1: Pa tudi niso po vrstnem redu. Grafi pa navodila.<br><br>R2: Aha prevodi.<br><br>**1:00:33**<br><br>FP1: Other ne more biti na koncu, ker other je po navadi viden tisti other, da bi bilo na koncu.<br><br>R2: Ker other so ostali, ne.<br><br>FP1: Ja, other je ostali, ker niso specificirani in po navadi tudi potem.<br><br>R2: Ostale vire po navadi daš na desno stran. Ni pravilno zaporedje tukaj ja.<br><br>**1:01:01**<br><br>R1: Prav ste potem ugotovili, med katero aktivnostjo poleti so bile povprečne koncentracije PM10 najmanjše?<br><br>FP2: Drugo<br><br>FP3: Jaz spet nisem, zato ker pač mogoče, da bi en tak stavek bil v bistvu tam kjer je prazen stolpec, ne pomeni, da je vrednost nič ampak da ni podatka. Tako da sem pač napisala, če je prazen stolpec to pomeni, da ni podatka in so to other, če ne pa so to vrednosti, ki so pač 0<br><br>R1: Odlično<br><br>R2: Ja, ker je tudi vprašanje tako zastavljeno, da ne veš, če je nič ali ni podatka.<br><br>FP3: Ja, ne vem.<br><br>R1: Ja kul, zelo smiselno.<br><br>R2: Ker lahko bi bili 3 možni odgovori: cook,	FP1: Then there are 2 on the graph.<br><br>R1: That was the point of this question, which will not be included in the report. Why does this happen? Obviously, because, as said earlier, I emphasize, that these are automatically generated reports. If a person was only present for 4 days, then only 4 days would be shown, apparently he was 6 days, and for the last 2 days we have no data on PM. However, there are some other data, about the activity that was recorded etc. And this is the kind of information a person should get, so we decided to give 1x 7 and 1x 6 because this are real data. So, we expect that someone who looks at this will see: aha, I was involved for 7 days in the winter and have all the data, but obviously for the summer I only participated for 6 days and have data only for the first 4 days about PM. Basically, it [data visualization] carries some information. But in the context of the questions, I agree, it doesn't make sense.<br><br>MP1: Yeah.<br><br>**59:27**<br><br>R1: Anything else about that? Do we go for the last one [Printed material, Page 6]? Any general recommendations?<br><br>**59:51**<br><br>FP1: I think the 'play' is missing in the explanation.<br><br>R3: Mhm, true.<br><br>R1: What's missing?<br><br>R3: 'Play' in explanation.<br><br>R1: Yeah, okay.<br><br>MP1: And they also aren't in order. The graph texts and translations.<br><br>R2: Yeah, the translationss.

smoking, clean ali pa other.<br><br>FP1: Aja, jaz sem to tudi čisto narobe.<br><br>FP1: Jaz sem pa napisala tisto, ko ni podatka, ne.<br><br>R2: Ker je 0 ne, si mislila?<br><br>FP3: Točno to ja.<br><br>FP1: Pa še za zimo sem odgovarjala.<br><br>R1: To bi se zdaj pričakovalo kakšnega matematika, ta detajl, ampak odlično!<br><br>MP2: Tukaj pod ”sport” manjka ”in” pol zadnja.. ker v razlagi je ”sport in” in..<br><br>R2: Pa sport out<br><br>R1: Kaj to sploh je ja, kategorija.<br><br>R2: aha, Sport in, ne?<br><br>FP3: Indoors pa outdoors ja.<br><br>R1: Je veliko lažje ja, če že bomo angleščino uporabljali.<br><br>**1:02:47**<br><br>R1: Kaj pa v tem primeru? Lahko delamo direktno primerjavo vrednosti pozimi in poleti?<br><br>MP2: V bistvu lahko ja.<br><br>FP1: Jaz pa sem napisala ne, ker niso iste skale.<br><br>MP1: Ja to pa res.<br><br>FP1: Da bi se vsaj približno videlo, da ja. Če bi bilo na isti skali ja, drugače ne.<br><br>FP2: Ampak veš da so ug na kubični meter, pol lahko primerjaš.<br><br>MP1: Ja.<br><br>FP2: Pa lahko vidiš, da je pozimi bistveno manj, kot pozimi.<br><br>R1: Da, Enote so in številke, in jih lahko	**1:00:33**<br><br>FP1: ‘Other’ means the rest, because they are not classified, and is usually placed at the end.<br><br>R2: Because ‘other’ means “the rest”.<br><br>FP1: Yes, ‘other’ means “the rest”, because they are not specified and they are usually placed in the end.<br><br>R2:Usually the others are on the right. Yeah, it’s not the correct sequence here yes.<br><br>**1:01:01**<br><br>R1: Right then, did you find out during which activity in the summer the average PM10 concentrations were the lowest?<br><br>FP2: Second<br><br>FP3: Again – maybe there should be one sentence regarding the empy columns that ”empty” does not mean that the value is zero but that there is no data. So I just wrote if there is an empty column that means “no data” and these are ‘other’, otherwise this refers to values that are 0.<br><br>R1: Great<br><br>R2: Yes, because the question is made the way that you don't know if it was zero, or was there just no information.<br><br>FP3: Yeah, I don't know.<br><br>R1: Yeah cool, very reasonable.<br><br>R2: Because there could be 3 possible answers: cook, smoking, clean or other.<br><br>FP1: Yeah, I was totally wrong about that.<br><br>FP1: I wrote that is means “no information is available”.<br><br>R2: Because it’s 0, right?

FP1: Ampak, saj to je fora, da vidiš kako je poleti res nizko.<br><br>R2: Ja, da bi isto skalo dali.<br><br>FP3: Ja malo je zavajajoče, ker maš pač zimo, poletje in intuitivno hočeš primerjat.<br><br>**1:03:42**<br><br>R2: Ja, ker nekdo bi rekel uf ne bi od daleč videltistih, 40 ug. Pol pa vidiš, da ni. Poleti je druga vrednost.<br><br>R1: Ja glede te primerjave smo že prej rekli, da bomo dodali en disclaimer, da ne primerjat, tu pa lahko spodbudimo k primerjanju.<br><br>FP1: Ja, ampak, samo je različne...4.<br><br>R1: Bodite pozorni na..<br><br>FP1: Veš, da so tako nižje. Zdaj pa bom jaz vprašala zakaj?<br><br>FP2: A se to pozimi toliko pozna, ker so delci? Kurjenje ali kaj?<br><br>R1: Ja, to je dejansko pričakovano, ampak v to se pa nismo spuščali.<br><br>FP1: Ja ampak tako pravim. Nekdo ki pa ne zna tako grafa brati, bo rekel tam je več, tam je manj ne… če bi meli pa obje enako visoko…<br><br>R2: Ja, če je skala enaka ja. Ker skala je dosti različna.<br><br>FP1: Ja<br><br>FP3: Ja pa mislim, da je večji učinek, če vidiš recimo, da imaš pozimi od 30 do 40, medtem ko poleti pa maš 3 ali 4 ali 5.<br><br>**1:04:37**<br><br>R3: Ker je pa tukaj, mogoče bomo mogli malo bolj jasno napisati. Ker to je bila ideja prejšnjega grafa, da prikažemo te relativne razlike med sezonami. Ta graf pa bi predstavljal relativne	FP3: Exactly.<br><br>FP1: Oh, and I also answered for the winter.<br><br>R1: I would have expected that kind of detail from some mathematician, but great!<br><br>MP2: Here under the “sport” is missing “in”, because at the text it is “sport in”.<br><br>R2: And sport out<br><br>R1: What is it, yeah, category.<br><br>R2: Aha, “Sport in”, right?<br><br>FP3: Indoors and outdoors yes.<br><br>R1: It's much easier, yes, if we use English.<br><br>**1:02:47**<br><br>R1: And how about in this case? Can we do comparison between winter and summer?<br><br>MP2: Basically yes.<br><br>FP1: I wrote no, because scales are not the same.<br><br>MP1: Yeah, that's true.<br><br>FP1: If it would at least look approximately the same. If on the scale was the same scale, then yes, otherwise no.<br><br>FP2: But you know, that they are displayed in µg per cubic meter, so you can compare.<br><br>MP1: Yeah.<br><br>FP2: And you can see that in summer it is much less compared to winter.<br><br>D. Yes, there are units and numbers, so we can<br><br>FP1: But this is the point , now we can see how low it really is in the summer.<br><br>R2: Yeah, perhaps we should use the same scale.<br><br>FP3: Yeah, it's a little misleading because there is winter and summer, and you intuitively want to

razlike med aktivnostmi.<br><br>MP1: Ok ja, pol je to tako boljše.<br><br>FP2: Ok ja, samo pol dajte enega zraven drugega in pol je tako, Aha, lahko primerjam zimo – poletje...<br><br>R3: Razumem ja.<br><br>R1: Ni pa načina, da se tole…<br><br>FP1: Ali pa če daste enega pod drugega.<br><br>R2: Ja mogoče, da se potem ne gleda vzporedno.<br><br>FP1: Ja.<br><br>FP2: Ja.<br><br>MP1: Kaj pa če bi prekrili grafe? Je to možno?<br><br>FP2: Pol se bo izgubilo.<br><br>R1: Lahko se da na isti graf pa se da bara skupaj pa je ena barva poletje, ena pa zima pa se potem vidi.<br><br>R2: Ampak potem ne boš vedel ali je bilo kaj ali ni bilo.<br><br>MP1: Ampak potem se te razlike med aktivnostjo res ne bodo videle.<br><br>FP2: Ker potem tukaj imaš največ 5, ne.<br><br>FP3: Mislim, a je sploh smiselno, da delaš te razlike glede na aktivnost, glede na rezultate a ne? Če bi zdaj vzeli poletje, vidiš da pač kar koli delaš, je lahko razlika samo 4 in ne več.<br><br>R1: V tem konkretnem primeru moramo vedeti, ne vemo pa kako velja za vseh ostalih, skoraj 100 ljudi, ne.<br><br>R2: To je samo kot primer ne, lahko pa da bo en imel čist drugačne vrednosti, mogoče celo bolj primerljive.<br><br>FP2: Mhm.	comparethem.<br><br>**1:03:42**<br><br>R2: Yeah, because someone would say: uf, I would have not noticed those40 ug. But then you see it's not. There is a different value in summer.<br><br>R1: Yeah, we said earlier about comparing that we were going to add a disclaimer not to compare, but here we can encourage for comparison.<br><br>FP1: Yes, but they are different..4..<br><br>R1: Here we need to pay attention to…<br><br>FP1: Of course they are much lower. But now I'm going to ask why?<br><br>FP2: Are the winter concentrations much higher because there are particles? Or is this because of heating or what?<br><br>R1: Yes, this is actually expected, but we didn't go into that.<br><br>FP1: Yeah, but as I say. Someone who does not know how to read the graphs, will say there is more, there is less ... but if the scale would be the same ….<br><br>R2: Yes, if the scale would be the same yes. Because the scale is very different now.<br><br>FP1: Yes<br><br>FP3: Yeah, but I think it's more impactful to see that you have between 30 and 40 in the winter and 3 or 4 or 5 in the summer.<br><br>**1:04:37**<br><br>R3: Maybe we should write this more clearly. Because the idea of the previous graph was to show these relative differences between seasons. While in this graph, however, represents relative differences between

MP2: Kaj pa, da bi bi obrnil pa bi odzgoraj samo poleti bilo, spodaj pa zima pa bi bili 3 stolpci pa 2 vrstici.<br><br>FP2: Lahko bi tudi tako ja.<br><br>R1: Mhm.<br><br>R3: Zanimivo.<br><br>R1: Ja, sicer te bo verjetno spet malo vleklo gledat, ne?<br><br>R2: Ja.<br><br>R1: Mogoče še res eno jasno opozorilo, da so skale različne.<br><br>FP2: Da je point tega grafa, da primerjaš glede na različne aktivnosti, ne na letni čas.<br><br>R3: To ja.<br><br>**1:07:03**<br><br>MP1: Kdo bo pa te grafe bral oz. gledal? Če je to splošna javnost ali so to strokovnjaki? Se od njih pričakuje, da bodo te razlike zaznali?<br><br>R1: Ja, mi delamo neko univerzalno poročilo, enako za vsakega, ne glede na to ali je človek družboslovec, naravoslovec …<br><br>R2: Tudi po izobrazbi smo imeli čisto različne udeležence ne, eni tudi nič angleško ne znajo, recimo.	activities.<br><br>MP1: Ok yeah, then this is better.<br><br>FP2: Ok yeah, just put one next to the other, and I can realisze to compare winter – summer.<br><br>R3: I understand.<br><br>R1: But the way to do this…<br><br>FP1: Or if you put one under the other.<br><br>R2: Yeah, maybe. So it doesn’t look paraller.<br><br>FP1: Yes.<br><br>FP2: Yes.<br><br>MP1: What if you overlap the graphs? Is this possible?<br><br>FP2: but then something mightget lost.<br><br>R1: It is possible to put the bar charts on the same graph and use one color for summer and another one for winter.<br><br>R2: But then you won't know if there was something or not.<br><br>MP1: But then these differences between activities will not really be seen.<br><br>FP2: Because here you have maximum 5 of them here.<br><br>FP3: But does it even make sense to show these differences by activity, by results, right? If you now for example look at the summer, you see that whatever you do, the difference is only 4 and never more.<br><br>R1: This is perhaps the case in this individual, but we don't know how it applies to everyone else, since there is almost 100 people.<br><br>R2: This is just an example, but someone else might have completely different values, perhaps even more comparable ones.

	FP2: Mhm.<br><br>MP2: What if you turn it around, display the summer on the top, and winter on the bottom. So there would be 3 columns and 2 rows.<br><br>FP2: it's possible yes.<br><br>R1: Mhm.<br><br>R3: Interesting.<br><br>R1: Yeah, but then again, you might want to compare them again?<br><br>R2: Yes.<br><br>R1: Maybe we need to put one clear discalimer that the scales are different.<br><br>FP2: Yes, that the point of this graph is to compare by different activities, not by season.<br><br>R3: exactly.<br><br>**1:07:03**<br><br>MP1: Who will read these graphs? General public or experts? Are they expected to perceive these differences?<br><br>R1: Yes, we make one universal report, the same for everyone, whether the person background is in social science, or natural science…<br><br>R2: We had diverse participants, also by education, even some who don't know any English, for example.

Section 7: Discussion Part 4

**1:07:44**<br><br>R1: To je to kar se tiče vizualizacij. Je kdo kaj pogrešal? Vem da si vam je težko to predstavljati. To je v bistvu tisti glavni del informacij, ki jih boste dobili v obliki tega poročila. Ponovno poudarjam, surovi podatki, ki jih je ogromno, so vedno na voljo pa lahko sami,	**1:07:44**<br><br>R1: That's about these visualizations. Did anyone miss anything? I know it might be hard for you to imagine. This is basically the main piece of information that you will receive in the report. Again, the raw data, which are there are plenty, are always available. So you can just ask for

se sami matrate z njimi.<br><br>FP1: Kaj pa zaključek v enih dveh treh stavkih personaliziran za tisto osebo opisno, ker ne bodo znali vsi grafov brati?<br><br>R1: Vključena, zaenkrat, so ena zelo generalna priporočila, razlaga tudi vseh parametrov, kaj pomeni, kaj so viri onesnažil, kako se obnašajo, kaj se da narediti, tudi za tisto, kar bomo operirali z absolutnimi vrednostmi, so ta priporočena območja, rangi… Tako, da ta informacija je noter. Je kar nekaj teksta.<br><br>FP1: Ampak za osebo, ne: spremenite način tega, bolj zračite prostor, manj kurite…to je ključno recimo. Mene bi zanimalo ali imam čisto zdaj noter ali moram bolj zračiti.<br><br>R2: Pa tudi kdaj moraš bolj zračiti…<br><br>FP1: To bi mene, kot uporabnika bolj zanimalo, ne grafi. Grafi so z mojega vidika zanimivi, ker pač smo v takem fochu.<br><br>R2: Saj priporočila bodo. Če boš recimo ti videla trenR1: popoldne imam pa preveč CO_2, OK mogoče se otroci preveč učijo, tudi ne dihajo, prezračiš. Zdaj za vsakega posebej pa ni poanta, da mi delamo. Vi boste dobili. Poanta je, da vi znate brat grafe aha CO_2 mam preveč.<br><br>R3: Po eni strani smo se tudi hoteli izognit malo temu, zaradi tega, ker konec koncev so nizko cenovni senzorji in ti podatki niso zanesljivi.<br><br>**1:09:54**<br><br>R1: Ja, to je del te zgodbe, da operiramo z relativnimi in to barvno skalo. Druga stvar je, da zakonodaja ne pokriva priporočenih ali pa mejnih vrednosti za notranji zrak in večino vseh parametrov je notranji zrak, kar niso PM2.5 delci za tiste podaja realne vrednosti in ta mejna. To pač pri človeku pričakujemo, da če pogledamo ta graf, evo tole bo recimo nekdo dobil. Bi mu moglo biti jasno, da je celo zimo oz. živi v okolju,	them and figure things on your own.<br><br>FP1: What about adding descriptive conclusions in few sentences personalized for that person? Because not everyone will know how to read graphs…<br><br>R1: So far there are very general recommendation included which explains all the parameters, what they mean, what are different pollutants sources, how they behave, what can be done. And also when we operate with absolute values, the recommended values and ranges are displayed... So there is a lot of information included. There is quite a bit of text.<br><br>FP1: But for a person you could adR1: “change the way you do that”, “ventilate more”, “reduce heating the fireplace” ... for example. I would be interested to know if I have good air in my apartment or should I open windows more often.<br><br>R2: And also when you have to open the window, right?<br><br>FP1: That [kind of information] would be more interesting to me as a user, not graphs. But of course from my personal point of view, graphs are interesting because I’m in that kind of job.<br><br>R2: There will be recommendations. For example, if you notice a trenR1: I have too much CO_2 in the afternoon, OK maybe the kids are learning in their room and you need to open their window... Now, there is no point that we do everything for each individual. You will get this information. The point is, you know to read the graphs and notice: aha there is too much CO_2.<br><br>R3: On the one hand, we also wanted to avoid a little bit of this because, after all, these are low-cost sensors and this data is not reliable.<br><br>**1:09:54**<br><br>R1: Yes, it’s part of the story that we operate in

kjer so koncentracije previsoke. To je to.<br><br>FP2: In kaj bi mogel narediti?<br><br>R3: To so pa posebej priporočila. Predpisana splošna priporočila, če nekdo vidi, da je situacija taka in taka, ukrepa tako in tako oz. neka priporočila, kaj lahko naredi. Spet je pa potem zelo specifično. Tudi recimo nam je bilo težko dati priporočilo, ker ne vemo razloga.<br><br>R2: Eni so bili recimo kadilci in tam se je zelo hitro videlo, da so parametri poskočili. Ampak to mora potem tudi posameznik pri sebi vedeti, zaradi česa je do tega prišlo.<br><br>FP2: Ja pa moraš vedeti, da to izhaja iz kajenja.<br><br>FP1: Ti mu moraš to povedati.<br><br>R3: To je recimo, kot eno izmed napisanih, da je eno izmed virov kajenje, kot vir delcev. To je v tekstu nekje. Potem pa oseba vidi, da so povečane koncentracije in reče aha, je potrebno bolj okno odpreti. To je neka logika, ki si mi predstavljamo, da bo nekdo ki bere to sledil takemu priporočilu.<br><br>**1:11:37**<br><br>R1: Čisto konkreten primer, ki ga je R2 omenila: oseba je bila opozorjena, da to ni dobro početi noter, je zamahnila z roko in rekla, saj jaz pa to pri odprtem oknu. Podatki kažejo tudi v obliki tega, da traja 6h, da pridemo na normalno vrednost. In ta informacija je noter, če jo človek hoče videti. Jo pa mi, razen tega dejanskega pogovora, ne bi mogli sami izluščiti niti pod razno. Ker to ne piše v dnevniku aktivnosti.<br><br>R2: Ker mi smo vseeno sedeli s tole žensko in smo gledali rezultate pa zakaj, in potem res vidiš. Pa je rekla: ne, ne, saj sem prezračila. Ampak ne, preden prideš ti dol, je tak zamik. Se ga še vseeno nadihaš.<br><br>FP3: Ne vem, če bi bilo ravno objektivno znanstveno, da zdaj pa vi vsakemu dajete	the relative values and usingcolor scales. Another thing is that the legislation does not cover the recommended or limit values for indoor air and most of all parameters are for indoor air. For PM2.5 particleswe use real values and display the limit value. If we look at this graph, we expect that for someone it will be clearthat winter he lives in an environment where concentrations are too high. That's it.<br><br>FP2: And what could I do?<br><br>R3: We will give specific recommendations. There will be these general recommendations what to do in differentsituationsSo some recommendations will be thereon what can he do. Then again, it can be very specific. For example, it will be difficult for us to make a recommendation if we do not know the reason for [high concentrations].<br><br>R2: For example, some of them were smokers, so the parameters quickly jumped. But then again, the individual should know what caused it.<br><br>FP2: But you have to know that it is due to smoking.<br><br>FP1: You have to tell him that.<br><br>R3: It is, for example, written that one of the sources of partickles is smoking. This is in the text somewhere. Then the person sees that the concentrations are higher and says aha, I have to open the windows. This is a logic that we imagine someone will follow the given recommendations.<br><br>**1:11:37**<br><br>R1: A very specific example that R2 mentioneR1: the person was warned that it is not good to [smoke] inside, she waved her hand and said, since I did it with the window open it’s OK. However, the data showed that it takes up to 6 hours to reach back to normal values. And that information is in there if a person wants to see it.

priporočila, ker to je pač neko mnenje že mogoče.<br><br>R1: Enostavno jih ne moremo.<br><br>FP3: Pač, da so priporočila, pol pa ljudje sami svoje zaključke morajo narediti.<br><br>R2: Ja, ker kako bo vsak posameznik živel, mu mi ne moremo diktirat, pač to je na njegovi, kako se bo odločil, al bo vpošteval...<br><br>R1: Smo dobivali pa že na terenu razno razna vprašanja, ne. Na primer jaz sem par krat bil vprašan: ali sem zdrav, je bilo vprašanje. Kako naj jaz odgovorim na to vprašanje, na primer, ne?<br><br>FP2: Verjetno ali je moje okolje zdravo, bi mogli biti vprašanje ne?<br><br>R1: Saj sem razumel vprašanje, ampak že tu težko dajemo odgovore, ne.<br><br>FP2: To se me zdi, torej, se pravi, če maš v nekem obdobju povečan CO2 pomeni, da treba prezračiti.<br><br>R2: Ja.<br><br>R1: Seveda.<br><br>R2: Ja vi boste dobili kot appendix oz. na konec poročila. Bodo potem vsi parametri ki so bili merjeni obrazloženi, kaj pomenijo, od kje so viri, od kje pridejo pa pol smernice kaj narediti, če je recimo preveč tega. Tako da se bodite<br><br>FP1: Pa zanimivo.<br><br>**1:14:06**<br><br>R3: Kako vas je pa recimo zmotilo, da niste podatkov videli vmes, ko ste to delali? Je bilo to zelo moteče, ste jih zelo hoteli videti?<br><br>FP2: Ne.<br><br>MP1: Ne.<br><br>FP3: Ker mislim, da bi potem tudi to vplivalo na	But we, apart from this actual conversation, would not be able to extract this infor on our own or even classify it under “other”. Because this is not written in the time activity diary.<br><br>R2: Because we sit down and discussed about the results with the woman and were wondering why is like that, and then you really see. But she said, no, no, I was ventilating. But before [the concentrations] get down there is this delay. And you are till breathing this bad air.<br><br>FP3: I do not know if it would be objectively scientific if you were making recommendations for everyone because that is perhaps already just an opinion.<br><br>R1: We just can't.<br><br>FP3: They are recommendations, but at the end the people have to make their own conclusions.<br><br>R2: Yes, because we can not dictated how each individual lives, but it is up to him how he will decide and wether to followthe...<br><br>R1: But we've been getting all sorts of questions during the field work. I've been asked a couple of times: Am I healthy? How can I answer such a question?<br><br>FP2: They should ask: Is my environment healthy?<br><br>R1: I understand the question, but it's hard to answer here.<br><br>FP2: So, if you see increased CO2 in some period, it means that you need to ventilate.<br><br>R2: Yes<br><br>R1: Of course.<br><br>R2: Yes, you will get it as an appendix, at the end of the report. There all the parameters will be explained; what they mean, where the sources come from, where they come from and also guidelines what to do if there is too much of

rezultate.<br><br>R3: Zato jih nismo dali.<br><br>R1: Ja.<br><br>R2: Ja, vrednost taka. Uh, to je skoraj kot holesterol, ko tam vidiš - uf, koliko ga imam.<br><br>R3: Mhm.<br><br>FP1: Je pa res ta stvar bila zelo nerodna, sem šla na Šmarno goro pa mi je dol padla in potem sem jo povsod iskala.<br><br>R1: Smo pa bili čisto navdušeni, ker v enem sorodnem, prejšnjem projektu smo tudi imeli en tak prototip, ki je pa bil pa res velik in se je nam zdelo res wow.<br><br>FP1: Hehe pa sem ga imela na torbici pa sem ga imela na pasu pa sem ga imela tam pa sem ga vedno pozabila.<br><br>R3: Recimo ne, da bi nam uspelo prepoloviti to velikost pa bi nam uspelo narediti, da je to za na zapestje. Bi bilo to boljše ali slabše?<br><br>FP2: To bi bilo veliko boljše.<br><br>MP1: Ja bi bilo boljše.<br><br>FP1: Pa še kakšen dober design za punce, potem pa sploh.<br><br>FP2: Ja, kot smo uro imeli.<br><br>R3: Recimo še pol te velikosti, ampak slaba stran bi pa bila, da bi bilo namesto 7 ur baterije, recimo 4.<br><br>FP3: Aja.<br><br>FP2: Ja, potem pa še polniti in ja…<br><br>R3: Ne, saj to je še tisto, ker je vedo neka stvar.<br><br>FP1: Potem bi ga mogli vedno polniti. Tudi tega smo vedno, še v službi, ker ni zdržal.<br><br>R3: Ja, res je. Ker zdaj to je to. Nekateri so bili	something.<br><br>FP1: Well interesting.<br><br>**1:14:06**<br><br>R3: How much were your bothered that you didn't see the data during the campaign? Was that very disturbing, did you really want to see them?<br><br>FP2: No.<br><br>MP1: No.<br><br>FP3: Because I think that would also affect the results.<br><br>R3: Well this is the reason why we didn’t give them.<br><br>R1: Yes.<br><br>R2: Yeah, seeing such a value. Uh, it's almost like when you see cholesterol - oh, how much cholesterol I have.<br><br>R3: Mhm.<br><br>FP1: But this thing [PPM meter] was really awkward. I went to Šmarna Gora and it fell down and I was looking for it everywhere.<br><br>R1: But we were really excited, because in one related previous project we also had one prototype like this, but it was really big and it seemed really wow.<br><br>FP1: Hehe, I had it on my purse and I had it on my belt and I had it in different places but I keptforgotting it.<br><br>R3: Let's say that we could cut that size in half, and have it as wristwearable. Would that be better or worse?<br><br>FP2: That would be much better.<br><br>MP1: Yeah it would be better.<br><br>FP1: And if there was a good design for girls,

taki ki so bili brez teh komponent, ki se povežejo s spletom. Ti so zdržali 15, 16, 18 h brez problema. Ta pa če je 6 h zdržal, je bil dober dan. Tako da ja.<br><br>FP2: Sigurno bo šlo naprej, to bo boljše<br><br>R1: Ja to je tak prototip narejen na projektu. To ni bilo niti stestirano.<br><br>R2: Homemade ja.<br><br>**1:16:10**<br><br>FP1: ICARUS 2 bo?<br><br>R in R1: Ne v taki obliki.<br><br>R3: Nekaj specifičnega iz tega ven si jaz predstavljam, da zdaj glede na načrte naprej, ki so, bomo pa sigurno imeli še kaj.<br><br>R1: Bolj v smeri neke bolj napredne obdelave podatkov pa zdaj to postaviti v kontekst mesta in dogajanja, na ta način. Imamo pa en soroden projekt CitieS-Health, ki je pa čisto citizen science. Se pravi v tem primeru ni šlo za citizen science ampak crowdsourcing ali pa zbiranje podatkov s pomočjo prostovoljcev. Tisti projekt pa naslavlja ljubiteljsko znanost, tema je hrup in zdravje, kjer pa prosimo ljudi, da si sami zamislijo, kako bi naslovili in raziskali nek problem z našo pomočjo in tudi s pomočjo senzorjev in ostale opreme. In trenutna izkušnja je pa, da ljudje želijo imeti vse servirano. Protokol, kako naj se nečesa lotijo. Nimajo toliko samoiniciative.<br><br>R2: Nimajo ideje, kako bi se lotili.<br><br>R1: Rabijo navodila ne.<br><br>FP1: Mhm.<br><br>**1:17:15**<br><br>FP3: A lahko pokažete še tisto, kot ste rekli, ene primere?	then definitely.<br><br>FP2: Yeah, like the Garmin watch, for example.<br><br>R3: Let's say if it was half the size, but the weakness would be that instead of 7 hours of battery life, it would last only 4.<br><br>FP3: Yeah.<br><br>FP2: Yeah, then charging it and …yeah…<br><br>R3: Well, there is always something.<br><br>FP1: Then you would always charge it. It was the same for this one we had, charging it at work, since it didn't last.<br><br>R3: Yeah, it's true. Because now that's it. Some were without these components that connect to the web. They lasted 15, 16, even 18h without a problem. But for this one, if it lasted 6 hours, that was a good day. So yes.<br><br>FP2: It [development] will certainly go forward. It will be better.<br><br>R1: Yeah, it's a prototype made within a project. It wasn't even tested.<br><br>R2: Homemade yes.<br><br>**1:16:10**<br><br>FP1: Will there be ICARUS 2?<br><br>R and R1: Not in that form.<br><br>R3: I think something specific will come out of this one, I imagine there will be plans ahead.<br><br>R1: More in the direction of some advanced data processing in the context of city and events. In this way. We have one related project called CitieS-Health, which is purely citizen science. That is, in this case, it was not citizen science but crowdsourcing or data collection with the help of volunteers. The other project addresses citizen sciencewithin the topic of noise and health, where we ask people to imagine for themselves

R1: Aja, ja, ja.

FP2: Vidiš bova lahko primerjali, koliko je pri tebi v mestu in koliko je pri meni na vasi.

R2: To potem lahko podebatirate med sabo ja.

R1: Sendwiče se pojejte prosim.

1:17:30

R3: Medtem, ko R1 to išče, bi pa jaz še vprašal: kako se vam je pa to danes zdelo? A je to v redu pristop?

FP1: A smo prvi?

R3: Ja prvič imamo tak focus group.

FP1: Velik si dal razpored..

R1: Ja, ne, tisto smo datum izbrali [Doodle]. To je bilo bolj razumeti da bo op 15. Zdaj pa jutri bo po mojem prepovedan spoh organizirat. Bomo to v obliki spletnega vprašalnika poslali ostalim.

FP1: Mogoče ste malo feedback dobili.

R1: Jaja, to je bil namen.

1:18:06

R3: Se vam ni zdelo preveč te vprašalnike reševat pa take stvari? Je bilo to v redu teh 10 minut, ko ste pisali?

FP2: Ja.

R3: OK. Kul. Je bilo dost debate.

FP1: Saj tudi vidiš kako je zanimivo. Eni so razumeli, eni niso nekaj. Kako različno gledamo.

R2: Ja, ker človek tudi različno razmišlja. Vsak malo drugače.

FP1: Ja, ja.

R3: Saj ni treba vsega pohvaliti. Če imate kakšno idejo, bomo zelo veseli še kakšne kritike.

how to address and investigate a problem with our help and also with the help of sensors and other equipment. And the current experience is that people want to have everything served. A protocol for how things should be handled. They don't have that much self-initiative.

R2: They have no idea how to handle it.

R1: They need directions.

FP1: Mhm.

1:17:15

FP3: Can you show the examples, you mentioned earlier?

R1: Yeah.

FP2: You can see how much [air pollution] you have in the city and how much I have in the village

R2: Then you can talk about it with each other yes.

R1: And please eat the sandwitches.

1:17:30

R3: While R1 is looking for it, I would also ask: What do you think about today? Is this approach ok?

FP1: Are we the first?

R3: Yes, we have such a focus group for the first time.

FP1: You put quite a long time line..

R1: Yes that was just to choose the date [Doodle], so we knew to organize this after 15th. Now I think that from tomorrow onwards it will not be even allowed to organize gatherings [due to COVID 19]. We will send an online questionnaire to the rest of the volunteers.

FP1: Maybe you got some feedback.

	R1: Yes, that was the purpose.<br><br>**1:18:06**<br><br>R3: Was it too much to fill the questionnaires andsuch things? Was it okay for those 10 minutes you were answering?<br><br>FP2: Yes.<br><br>R3: OK. Nice, there was a lot of debate.<br><br>FP1: It was also interestingto see how some of us understood, while others did not. You could see how all of us look things differently.<br><br>R2: Yes, because we all think differently.<br><br>FP1: Yeah, yeah.<br><br>R3: You don't have to praise everything. If you have any ideas, we will be very happy to hear alsosome criticism.

Section 8: Conclusions and socialising

**1:18:39**	**1:18:39**
R1: Ja za občutek. To je pa zdaj tak, ne bo v poročilu, ampak da razumete, boste lažje razumeli, ko boste gledali. Zdaj kaj smo mi naredili. Mi smo stestirali kakšen tip rezultatov dajejo ti instrumenti. Potem smo recimo vzeli oz. R3 konkretno je vzel tole napravo, jo postavil skupaj z tem Grimmom, ki je mednarodno priznan, drag, natančen instrument in dobimo takole korelacijo. In potem rečemo wow, bili smo presenečeni, to je dober rezultat. Mi si upamo zaradi tega vam tudi dati absolutne številke in to je to. Potem smo pa vzeli to napravo, ki pa meri tudi delce in smo naredilo to primerjavo in nekakšen trend se tam nekje v ozadjih vidi ampak v resnici ne. Zato smo ne npr. odločili, da vam tega rezultata sploh ne bomo dali, ker itak imate s sabo tega, ki je bolj relevanten za vas. Podobno smo naredili npr. za CO2, kjer smo pa videli, da tale naprava sicer kaže isti trend, ne pa	R1: Yes for the feeling [Slide 10 in the Powerpoint]. That's it, This won't be in the report, but just for you to understand, it will be easier to understand when you'll look at your report. Now what have we done. We have tested what type of results these instruments produce. Then we took, or more precisely, R3 took thisdevice [shows a sensor device physically], put it together with this Grimm, which is an internationally recognized, expensive, accurate instrument, and we get this kind of correlation. And then we said “wow”, we were surprised, that's a good result. Because of that, we dare to give you absolute numbers, and that's it. Then we took this device [shows another device], which also measures particles, and we made this comparison, and a trend is seen somewhere in the background, but not really. Therefore, we decided not to give you these result at all

istih absolutnih vrednosti. Se pravi, tako kot sem prej rekel, če bi hoteli vam dati dejanske številke, bi mogli priti v vaše gospodinjstvo, ker so te naprave odvisne od temperature, vlage, ostalih pogojev, specifičnih za vaš prostor, porabiti par dni, da bi skalibrirali potem pa bi vam jo šele dali. Tega luksuza nismo meli, zato smo videli, aha pri viru CO2 gor in dol kaže prav, ampak ne pa absolutne številke, in smo vam dali relativno skalo.

1:20:12

Isto smo naredili, no saj to je isto ta zgodba, ki je bila na prejšnji sliki, se pravi v primeru 18 ali koliko enot smo takole postavil, je nek za delce random number generatior, nima smisla tisti podatki, zato jih ne kažemo. Pri CO2 pa zelo jasni enaki trendi, ampak je pa offset za 1000 ppm in to je pač podlaga za našo odločitev. Kar je pa v bistvu bolj spodbudno za ta fitness for purpose ki mi stalno poudrajamo, ne. Odločiti se je treba na začetku v kakšen namen uporabljamo te senzorje. To ni za regulativo, to ni zato, da bi hodili po inšpektoratih, vemo pa, da če prižgemo svečo, vidimo peak. Če uporabljamo dezodorante, vidimo neko dogajanje.

1:21:00

FP1: A dejanjsko se to vidi

R1: Krasen primer je iz Češke: sta bila mož in žena, ki se neverjetno rada parfumirata. In točno se ve. Ženska se zbudi ob 6 zjutraj začne škripat tisto, pride do 1200 ppbjev, kar je ta zgornja meja, ki jo je ta instrument sposoben doseči. Znori instrument, potem malo pade dol, se še mož zbudi in še on začne in spet začnemo vse znova in vsak dan isto.

Vsi: Hehe, wow

R1: In to je podatek, ki ga ti želiš imeti. Zdaj nekdo, ki pač hoče malo več, zdaj vas malo navajam k temu, da nam boste malo težili še za kakšne surove podatke pa se boste še sami igrali,

because you were carrying something that is more relevant to you. We did similar for CO2, where we saw that this device shows the same trend, but not same absolute values [Slide 11]. That is, like I said earlier, if we wanted to give you actual numbers, we would need to cometo your place because these devices depend on the temperature, humidity, other conditions specific to your space, spend a couple of days for calibration and only after then we would give it to you. We didn't have that luxury, so since we saw – aha, at the CO2 trends go up and down correctly, but not theabsolute values, so we gave you [results in] a relative scale.

1:20:12

R1: We did the same thing [Slide 12], and this is the same story that was in the previous picture, that in case 18 or so units which we placed like this, turned out to be some random number generator for the particles, and the data doesn't make sense, so we don't show them. With CO2, the same trends are very clear, but it is offset by 1000 ppm which is the basis for our decision. Which is basically more encouraging for the so called “fitness for purpose”, which we constantly emphasize. It is necessary to decide at the beginning to what purpose these sensors are used for. It's not for regulative purposes, nor to run around at inspectorates, but we know that if we light a candle, we see a peak [Slide 13]. If we use deodorants, we see something is happening.

1:21:00

FP1: Ou, you can actually see it.

R1: We have a nice example from the Czech Republic: both husband and wife who incredibly liked to use perfumes. And it was possible to know exactly. A woman wakes up at 6 in the morning starts to spray and it goes up to 1200 ppbs, which is the upper limit that this instrument is cabable to reach. The instrument goes crazy, then falls down a little, the husband

so npr. to za neke generalne ugotovitve, da po navadi je nekaj teh dnevnih dogodkov, da povzročijo, da se začne nekaj dogajati. To je po navadi, ko začneš kuhati, ali začneš ogrevati stanovanje. In informacija v resnici kaj dobiš je, koliko ur to traja, da se stanje, ne vem, normalizira ali pa če odpremo okno, koliko časa traja, da se stanje normalizira. To sta 2 podobna primera, ampak zopet mi moramo vedeti kaj se je takrat dogajalo. In že tisto vam je bilo tečno za vsako uro natančno popisovati. Včasih pa so ti dogodki dolgi 10 min in jih niste zabeležili. Se pravi in jih nimate ne vi in ne mi in je težko to interpretirat. Potem nasploh lahko damo generalno opazko pozimi - premalo zračenja. Ker mi imamo v Sloveniji, tak koncept od naših mam: prepih pa bomo vsi zboleli, ne vem kaj se bo zgodilo…Ugotavljamo pa tudi to: to nam krasno kažejo podatki o teh lastnostih stanovanja. Ljudje, ki so investirali veliko denarja v neke rekuperacije in sisteme, mislijo, da je stvar popravljena, jaz sem dal veliko denarja in zdaj lahko sedim doma. Ti sistemi vključno s to zgradbo ne delujejo tako kot je treba in niso učinkoviti. In ta instrument ti pokaže, da je treba odpirat okna, ne glede na to kaj imaš.	wakes up and he starts with parfuming and we start all over again and every day the same.<br><br>FPs: Hehe, wow, super.<br><br>R1: And this is the piece of information you want to have. Now someone who wants little more, I am encouraging you a little to require the raw so aou can play with it by yourself. For example, these are now some general observation that there are usually some daily events that causes something to happen. This is usually when you start cooking, or when you start heating your apartment [page 15]. And the information you get out of this boils to the point that how many hours it takes to get back to the normal situation, So if we for example open a window, how long does it take to normalize. These are 2 similar cases, but again we need to know what was happening at the time. And for you, it was already annoying to write down [your activities] accurately for each hour. But sometimes these events are 10 minutes long and you have not recorded them and neither you nor we have them so it is difficult to interpret. Then we canmake a general observation in winter - not enough ventilation. Because in Slovenia we have this concept from our mothers: the draft. We will all get sick, I don't know what is going to happen… But we also find out the following: the data on characteristics of the apartments show us this beautifully. People who have invested a lot of money in some recuperation systems think this is it, I invested a lot of money and now I can sit at home. These systems, in the houses, do not work as they should and are not efficient. And this instrument shows you the need to open windows no matter what you have.
R2: Naravno zračenje.	R2: Natural ventilation
R1: Naravno zračenje, ne. In potem je to en lep primer v eni fancy hiši. Veččlanska družina: ko gredo večer spat, se dvigne koncentracija vse do zjutraj, ko odprejo okno. Se pravi čas in trajanje zračenja, odvisno od lokacije. Kaj smo imeli tu en primeR3: aha, mislim, da je tu tole.	R1: Natural ventilation yes. And then this one is a fine example in a fancy house with a multimembered-family: When they go to bed in the evening, their concentration rises until the morning when they open the window. I.e. the

1:23:27

R3: Aha ta NO2 je tudi zanimiv, kjer lahko vidimo kako se v istem času, mislim, da je ista skala spodaj. Kako so koncentracije CO2 padle, NO2 pa je narastel.

FP3: Ko odpreš okno ne?

R3: Ja. Ampak je še vedno ne, te NO2 številke so še vedno nizke, tako da to ni visoka številka.

R1: Je pa dobro to vedeti, če stanuješ zraven ene zelo prometne ceste. Pač ne boš šel zračiti, ko je rush hour. In jaz potem zdaj kakšnemu rečem: v teh 14 dneh, čeprav vidiš rezultate, je dovolj časa, da vidiš kako živiš in vidiš kaj se dogaja v stanovanju. Ne rabiš tega instrumenta vedno imeti. Po moje je 2 tedna bilo res dovolj.

1:24:13

To je to, gremo naprej.

R1: Ljudje se zgrozijo za tisti del podatkov, ko imamo GPS lokacijo. Če jih potem malo geostatistično obdelamo, potem to zgleda takole: tam si doma, 90% časa. Nekje si v službi, tam je pa potem ena trgovina v okolišu.

Vsi: Hehe

R1: Topla voda, ampak ljudje so to videli hm, potem nam pa ni bilo več všeč. Tale se si ga je nadel na kolo pa se je v Idrijo odpeljal in to je spet krasen primer. Jaz vam dam te podatke pa se lahko sami igrate, ki GIS obvladate. Ampak, ko sem jaz to zrisal sem rekel: aha to je too good to be true. Sem poklical: a je to možno? Jaja, pravi človek. Ko sem se peljal do tega Vršnikevega klanca, je bilo delo na ceste, se je prašilo, potem pa je začel s smeri gozdov pihati lep svež zrak in so se dejansko na eni točki koncentracije obrnile ter podobne stvari. Potem človek lahko gleda: s kolesom v službo pa nazaj? Dopoldne, v rush hour? Tudi to je z soglasjem človeka, ki je to delal in zbiral podatke iz Črnuč na reaktor k nam zjutraj. Se je poskusil malo izogniti med njive, ker

time and duration of aeration depending on the location. What we had here is one example: aha, I think this is it. [Page 16]

1:23:27

R3: And this about NO2 is also interesting where we can see how at the same time, I think it's the same scale below [Page 17]. When CO2 concentrations dropped the NO2 increased.

FP3: When you open the window, right?

R3: Yeah. But these NO2 values are still low, so it's not a high number.

R1: But it's good to know if you live next to a very busy road. You shouldn'topen windows during rush hours. Given this, I then sometimes say: in those 14 days, even though you see the results, it is enoughto see how you live and notice what is happening in the apartment. You don't always need to have this instrument. To my opinion, 2 weeks was really enough.

1:24:13

This is it, let's move on.

R1: People get shocked for that part of the data when we have a GPS location. If we then process them a little geostatistically, then it looks like this [Page 19]: this is your home, you spent 90% of the time there. Your workplace is here, and then there is one shop in your neighbourhood.

All: Hehe

R1: This is a bit rediscovering, hehe, but ones people saw it, they didn't like it anymore. There isguy, who put it on his bicycle and then cycled to Idrija, and it is again a great example [Page 20]. I can give you this kind of information and you can play with it by yourself, those of you who know to use GIS. But when I drew it I said to myself, aha that's too good to be true. I called him: is that possible? Yes, the man said. “As I drove up to Vršnik, there were roadworks, and

pravi da je zelo tečno po cesti pa v bistvu ne pomaga, ker tisti dan pa piha iz te smeri, ne. Potem pa ko gre popoldne nazaj, je pa, saj ne vem, je pa obratno.

R3: Ja to je že oboje ista slika, se mi zdi.

1:26:01

R1: Ne, manjka mi. Ista taka za popoldne pa je čisto drugačna situacija, ker ni rush hour ali pa piha v drugo smer. Tukaj je eden šel na sprehod nekam, na Nanos ali kam. Se tudi vidi, ko hodiš po makadamski cesti, da se prši na okrog. To je spet podatek, ne? Aha, zdaj so pa vrednosti višje. Pa saj to je ta prah, ki je na tleh. Ta pa ni problematičen v resnici ali pa vsaj ne tako kot en prah v nekem drugem okolju. Zopet poudarjam celi čas: vi ste tisti, ki znate osmislit te podatke.

R1: Zelo smo razmišljali za to prostorsko komponento, ker je marsikdo rekel, ja zanimam me. In nismo bili dovolj pametni, da bi res našli en način kako postaviti to v prostor. Izluščiti ene take posamezne dogodke ni problem. Ampak to so eni takšni dogodki, ko so nas ljudje opozorili ali lahko pogledaš, kaj se dogaja takrat in takrat. Če se kdo od vas to spomni, z veseljem naredimo. Ne moremo narediti za vse podatke, ker ne poznamo konteksta. Ko smo pa poskušali agregirati na neko celico, na 500x500m vse povprečiti recimo ne, smo pa dobili tako karto, ki vsak, ki je videl, tudi raziskovalci so mislim, da to karto porazdelitve koncentracije, modelna napoved zraka za Ljubljano. Kar ni ne, informacija ne nosi ničesar. Ne nosi ne podatka koliko meritev je bilo narejenih v tem kvadratu, kaj zakaj je zdaj ta rdeča pika, a je bil človek zdaj tukaj 1 min ali je bil 4 h, kakšno dozo, kakšno izpostavljenost je dobil. Nekako nismo prišli skupaj, da bi ugotovili na kakšen način to zdaj dati. Da ne govorim še pol o drugih izzivih, ki se jih lotevamo. Tu bi pa mogli uporabiti metode strojnega učenja: ko smo v hiši, nam GPS signal razmeče in se ne da dešifrirati ali je bil človek odznotraj ali odzunaj. Nimamo podatka za vsako

dusty, and then the wind started blowing fresh air towards the woods, and in fact at one point the concentrations turned.” Then one can think: Should I cycle to work? In the morning, in rush hour? Again, we are displaying this with the consent of the man who was doing it and collecting information from Črnuče to the Reaktor in the morning [Page 20]. He tried to avoid by cycling through the fields because he says that it is very hectic on the road, but it basically didn’t help, because that day wind blew from this direction. Then when he went back in the afternoon, well, I don't know anymore, but it's the other way around.

R3: Yeah it's both the same picture, I guess.

1:26:01

R1: No, it is misssing. There was a same map for the afternoon, however, with a completely different situation because it was not rush hour or the wind was blowing in the other direction. Here one person went for a walk somewhere, to Nanos or where [Page 20]. It can also be seen how walking on a dirt road lifted particles. This is again information, you see? Yeah, but the values are higher now. Because this is the dust that is on the floor. This one is not really that problematic, or at least not as problematic as dust in some other environment. Again: you are the one who know how to make sense of this information.

R1: We were thinking about this spatial component a lot because many people said, yes, I'm interested. And so far we haven't been smart enough to find a way to really displaythat tially. Isolating out a single event is no problem. But these ones are the ones when people contacted us if we could look at what is happening then and then. If any of you remember something, we're happy to do it. We can't do it for all the data because we don't know the context. However, when we tried to aggregate on a cell, to average everything at 500x500m let's say

minuto. Včasih lahko dešifriramo z drugimi GIS podlagami: a zdaj si na avtocesti, verjetno si se peljal, lahko ugotoviš iz hitrosti, ki je tudi podatek, ni pa nujno. Lahko si pa obtičal na sredini zamaška. Skratka ni enostavno v kontekst spraviti te podatke, zato bomo mogli biti malo bolj, to bo v bistvu R3 delal doktorat med drugim. In se veselimo teh in bomo verjetno zavrteli tudi te številke potem.	[Page 21], we got a map that everyone who saw it, even the researchers, think that this map is concentration distribution, modelled air forecast for Ljubljana. Which it is not, this information carries nothing. It does not bear the data of how many measurements were made in this square, neither what is this red dot, was he here for 1 min or 4 h, what dose, what kind of exposure he got. We Somehow didn't figure out a way to display it. Not to mention the other challenges we face. But we could use machine learning methods here: when we are indoors, the GPS signal is dispersed and cannot be decrypted whether the person was inside or out. We don't have data for every minute. Sometimes we can interpret with other complementary data: Ssince you were on the highway so you were driving, you can tell this from the speed, which is also information, but not necessary. But you could have been stuck in stopper traffic jam. In short, it is not easy to put this information in context. So we need to find new ways, and among others, R3 will work on this for his PhD. And then we can look at the data again.
R3: Ja definitivno.	R3: Yes definitely.
**1:28:31**	**1:28:31**
R1: Pa zdaj, ko ste bili tako pridni, vas jaz lahko spomnim. Nekaj smo pa pozabili, še en senzor je bil, ki ga nismo omenjali. Se kdo spomni?	R1: Now that you have been so diligent, I can remind you. But we forgot something. There was another sensor that we didn't mention. Does anyone remember?
FP3: Zapestnica. Ta rumena.	FP3: Bracelet. The yellow one.
MP1: Ja.	MP1: Yeah.
FP2: Saj je že tukaj pisala.	FP2: It was written here already.
R1: Aja.	R1: Yeah.
R3: Aja, ok.	R3: Yeah, okay.
FP2: Silikonska zapestnica.	FP2: Silicone Bracelet.
R1: V tem poročilu teh rezultatov ne bo. So še v izdelavi, ker je analitika strašno komplicirana in draga. Tako, da tisto dobite, ko bodo, naknadno.	R1: There results will not be in this report. They are still been analysed, because analytics are
FP1: Tisti so potem bolj zanimivi, a ne? Glede uporabe.	
R1: Ja.	
R2:Ste ga nosili nonstop	
FP1: Tisto je bilo vedno.	
R2: Pa pri kajenju…	
R1: Ja, to je pa ta agent-based modelling, ki se pa na podlagi nekih predefiniranih tipičnih skupin ljudi da predvideti kje živijo, kako živijo in kje se gibljejo. In če ima človek ta podatek, potem ga naloži na podatek o npr. modelna napoved	

kvalitete zraka v mestu in potem raba prostora in ostale infrastrukture. In se da izračunati pri kateri aktivnosti, v kateri lokaciji si dobil kakšen del dnevne doze npr. In ugotoviti ali je ta smiselni izračun ali zmodelirani podatek res smiselni, se rabijo podatki kot ste jih vi dejansko zbirali in se naredi ta primerjava in se jih potem modelna sposobnost ugotovi. Tole so spet eni naši izzivi in challengi.

R3: Ker ena izmed stvari, ki jih bomo preračunali, je tudi to kakšno dozo delcev ste dobili. Tega sicer zdaj na koncu nismo vključili, ker so relativno manj zanesljivi pa ravno teh podatkov o mikrolacijah nismo imeli, ampak načeloma pa se gre za to, da, ko vi recimo tečete po cesti, je vaš srčni utrip višji, dihanje je bolj globoko, več zraka pride skozi vas in imate večjo dozo delcev. Tako da se tudi s tem zdaj ukvarjamo pa poskušamo nekaj pridobiti iz teh podatkov.

R1: Dobro, da si to omenil, ker smo pozabljali pa upamo, da boste vi to malo dešifrirali s tega grafa.

R: Aja, ja.

1:30:57

R1: Zakaj je tukaj dodan še ta hearth rate zraven koncentracije delcev? Tu se pričakuje, da se vidi pri kateri aktivnosti ste imeli višji srčni utrip, kar pomeni, da je tudi večje dihanje in če se to prekriva z območji, kjer so večje koncentracije, ste avtomatsko dobili večjo dozo relativno.

MP1: Kaj potem iz tega sledi? V bistvu odsvetujete fizično aktivnost na prostem v bolj onesnaženih območjih.

R1: Če to človek vidi sam zase, bi si jaz upal trdit, da ja.

MP1: Ali pa tudi kolesarjenje na delo recimo.

R2: Ja najbrž ni primerno ne.

MP1: V primerjavi z voznikom avtomobila, to se

terribly complicated and expensive. So you'll get this later.

FP1: Those are more interesting then, aren't they? In terms of use.

R1: Yes.

R2: Yuo carried it around the clock.

FP1: Yeah, we alwayshad it.

R2: And when smoking ...

R1: Yes, this is this agent-based modeling, based on some predefined typical groups of people, it can predict where they live, how they live and where they move [Page 22]. And if they have this information, then it can be combined with information about e.g. model forecast of air quality in the city and landuse and other infrastructures. And it is then possible to calculate during which activity, in which location you got part of your daily dose for example. And in order to determine if this calculation or the modeled data really makes sense, the kind of data you actually collected is needed and this comparison is made and then the modelsare assessed. These are again some of our challenges.

R3: Because one of the things we're going to calculate is the particle dose you got. Although we did not include this in the end, because they are relatively less reliable, and we did not have these microlocation data, but in principle, when you are running on the road, your heart rate is higher, your breathing is deeper, more air comes through your lungs and you have a larger dose of particulate matter. So we also work with that kind of thingsand are trying to get something out of this data.

R1: Good that you mentioned it, because we almost forgot. We hope you will interpret it from this graph.

me zdi absolutno...<br><br>R3: Sigurno imamo zdaj za enkart še premalo podatkov. So pa bile narejene zelo zanimive študije na to temo, kjer so primerjali rekreacijo ljudi v velikih mestih, koliko časa se lahko rekreiraš zunaj, preden pozitivne stvari, ki jih dobiš od rekreacije, so zanemarjene zaradi negativnih stvari zaradi onesnaženja. In v najbolj onesnaženih mestih na svetu je to že po 15 min. Kjer recimo ne vem kakšna Stockholm ali pa Helsinki pa praktično ni, tam pa ni neke časovne omejitve. Za Ljubljano pa ne vem, ni bilo narejene neke študije.<br><br>R1: Jaz mam isto dilemo ne, ker živim v Ljubljani in grem občasno tečt in pomislim na to odkar sem v tovrstnih projektih in se izogibam. Nekaj časa sem konstantno tam ob Večni poti, tam že vohaš. Ne rabiš imeti nekega senzorja in pol namenoma zavijem v gozd rajši.<br><br>FP2. Tam pa se moraš za klope pošpricat.<br><br>Vsi: Hehe<br><br>FP1. Vedno se nekaj najde.<br><br>**1:33:10**<br><br>R1: Res vam hvala, da ste si vzeli čas. Za nas je bilo to zelo koristno. Če boste imeli potem še kakšno dodatno vprašanje, to bo komaj drug teden, ko vam bomo dali ta poročila.<br><br>FP2: Za nas je bilo tudi zanimivo.<br><br>MP1: Res.<br><br>**1:34:41**	R: Yeah, yeah.<br><br>**1:30:57**<br><br>R1: Why is this hearth rate added in addition to particle concentration [Printed material, page 4]? Here, it is expected that you can find out at which activity you had a higher heart rate, which also means greater inhaling and if this overlaps with areas where concentrations are higher, you will automatically receive a higher dose, relatively.<br><br>MP1: So the conclusion of this is? Basically, you are advised against exercising outdoors in more polluted areas, right?<br><br>R1: If a person sees this for himself, I would dare to say yes.<br><br>MP1: Or, like, cycling to work.<br><br>R2: Yeah, probably is not ok.<br><br>MP1: Compared to a car driver, that seems like...<br><br>R3: We certainly still have too little data. However, very interesting studies have been done on this topic, comparing the recreation of people in big cities, how much time you can recreate outside, before the positive effects you get from recreation are exceeded by the negativeeffectscaused by pollution. And in the most polluted cities in the world, this is already after 15 min. Where, for example, I do not know, Stockholm or Helsinki, it’s virtually nonexistent, there is no time limit. For Ljubljana, however, I do not know, I don’t think such study has been done.<br><br>R1: I have the same dilemma because I live in Ljubljana and when I occasionally run I think about it ever since I am in projects like this and I avoid it. For some time, I was constantly running along the Večna pot, and you can smell it already. You don't need to have a sensor. And because of this I rather choose to run in the

	woods.<br><br>FP2: But in the wood you have ticks you have to spray against<br><br>All: Hehe<br><br>FP1: There is always something to be found.<br><br>**1:33:10**<br><br>R1: Thank you very much for taking the time. This was very helpful for us. If you have any further questions about report, just ask. And I guess within fourthnight you will receive the report.<br><br>FP2: It was also interesting for us.<br><br>MP1: Yes.<br><br>**1:34:41**

4. List of suggestions from the participants and actions taken based on them

- Question about Task 3: "What the gray sections of the graph show. What are graph sections? Are these parts of the graphs? (showing on the graphs)"
  - ➔ "NA" was changed to "neznana aktivnosti// unknown activity"
- Comments on Task 4: "How do I know which are $PM_{2.5}$, and which PM_{10}." "Maybe it would be better if PM_1, $PM_{2.5}$ is indicated here in the graphs"
  - ➔ additional chart titles for PM_1, $PM_{2.5}$ and PM_{10} were introduced (they were left out unintentionally)
- "In the graph about meteorological parameters it would be better to have dates under each graph rather than just under the one on the bottom" "It would be clearer to me if it was below each graph. Otherwise you must almost have a ruler to read it."
  - ➔ date was added under each graph.
- "Yeah, even though when you wrote yellow for the summer, it looks more orange to me."
  - ➔ was not changed, as the point was the contrast between winter(blue) and summer (yellow)
- "Otherwise I see you have dates that in summer they are for every day and in winter for two days. I would unify that."
  - ➔ every 2 day was used to mark a line in the chart.
- "Maybe, there, the grid lines could be a little darker to be more clearer"
  - ➔ the gridlines at every second day were made darker.
- "The scale could be on both sides."

- ➔ Y-axis was added on the left of both winter and summer charts. And the y-axis fine lines were made darker
- "I just got the feeling it was summer. Maybe it's because this color is red."
  - ➔ A disclaimer sentence was addeR1: "Poletnih in zimskih grafov ni mogoce direktno primerjati, ker so prikazane vrednosti relativne.// The summer and winter graphs can not directly be compared, because relative values are displayed"
- A participant noticed CO_2 should be in subscript.
  - ➔ the use of accented letters (č, š and ž in Slovene) was difficult to implement in R, but eventually managed
- "I was a little confused here. NA means no data, or missing data, but there is a lot of these dots. It just confused me. I didn't know what to think. Let's say when we had a question - how much was the maximum $PM_{2.5}$ concentration: if I look at $PM_{2.5}$, it shows me 120 but it's grey"
  - ➔ "NA" was changed to "neznana aktivnosti//unknown activity"
- Dots displaying different activities in chart 3 hard to distinguish "I didn't see that." "But will the numbers actually be so small?" "I wrote that there was no sport activity because I didn't see the color", "Sport is marked orange", "Sport is marked pink"..
  - ➔ made the dots bigger, and use more distinguishable colour variations. The final report was in PDF form, which enabled the participant to zoom in.
- About 4. graph "Actually, I didn't understand what this first graph on the left is."
  - ➔ Added to the reporR2: "PM_1 does not have a limit value…"
- Debate about number of days displayed "[why] in here, it's for 7 days in winter and 6 days for summer"
  - ➔ Final report displays 8 days regardless of num of days participated
- Graph 5 misunderstandings "I think the 'play' is missing in the explanation." "And they also aren't in order. The graph texts and translations.", "'Other' means the rest, because they are not classified, and is usually placed at the end", "Again – maybe there should be one sentence regarding the empy columns that "empty" does not mean that the value is zero but that there is no data. So I just wrote if there is an empty column that means "no data" and these are 'other', otherwise this refers to values that are 0", ""Yeah, but as I say . Someone who does not know how to read the graphs, will say there is more, there is less ... but if the scale would be the same …."
  - ➔ translated activities to Slovene, placed "other" at the ends, and added sentence "Aktivnost brez stolpca pomeni, da te aktivnosti tekom raziskave niste opravljali"//Activity without a histogram pillar means that you did not undertake those activities during your participation" and Grafi omogocajo neposredno primerjavo koncentracij med posameznimi aktivnostmi in med sezonama. Bodite pozorni na y osi (navpicna os), saj so vrednosti prikazane na razlicnih lestvicah.// The graphs enable direct comparission of activities between the two seasons. However, pay close attention to the y-axis (vertical) and x-axis (horizontal), as the values are displayed in different scales.
- Additional suggestions for the report to contain: "What about adding descriptive conclusions in few sentences personalized for that person? Because not everyone will know how to read graphs…" "But for a person you could adR1: "change the way you do that", "ventilate more",

“reduce heating the fireplace” ... for example. I would be interested to know if I have good air in my apartment or should I open windows more often.”

- ➔ An appendix in the report was added (already considered to add this) where all parameters are explained, their characters, sources, health effects as well as suggestions to reduce ones exposure in additional to general guidance on how to improve indoor air at home.
